# Stereoselective Access to Diverse Alkaloid-Like Scaffolds via an Oxidation/Double-Mannich Reaction Sequence

**DOI:** 10.1021/acs.orglett.4c01924

**Published:** 2024-06-21

**Authors:** Charles P. Mikan, Joseph O. Watson, Ryan Walton, Paul G. Waddell, Jonathan P. Knowles

**Affiliations:** † Department of Applied Sciences, Northumbria University, Ellison Place, Newcastle upon Tyne NE1 8ST, United Kingdom; ‡ School of Natural and Environmental Sciences, Newcastle University, Newcastle upon Tyne NE1 7RU, United Kingdom

## Abstract

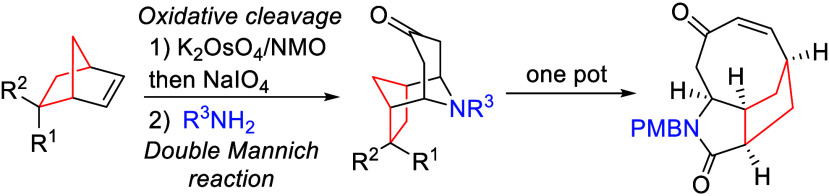

Sequential oxidative cleavage and double-Mannich reactions enable the stereoselective conversion of simple norbornenes into complex alkaloid-like structures. The products undergo a wide range of derivatization reactions, including regioselective enol triflate formation/cross-coupling sequences and highly efficient conversion to an unusual tricyclic 8,5,5-fused lactam. Overall, the process represents a formal one-atom aza-ring expansion with concomitant bridging annulation, making it of interest for the broader derivatization of alkene feedstocks.

Access to novel small-molecule scaffolds is of great importance to organic chemists and the field of medicinal chemistry in particular.^1−3^ Recently, there has been particular focus on forming scaffolds with high three-dimensionality as a result of the improved success of such compounds within clinical trials.^4,5^ Rigid three-dimensional (3D) scaffolds are of particular interest, with bridged bi- and polycyclic architectures being of particular importance as a result of their frequently potent bioactivity^6,7^ and associated current synthetic interest.^8−12^ Ideally, such systems would also offer the ability to perform orthogonal functionalization to enable facile library synthesis.^13^

Natural products have historically proven to be a useful starting point for drug discovery, with up to 75% of drugs being derived from or inspired by molecules originating in nature.^14^ The alkaloid family in particular has proven an excellent source of central nervous system (CNS)-active compounds, which is an area of medicinal chemistry that continues to offer significant challenges.^15−17^ However, synthetic access to alkaloid structures is frequently limited by complex syntheses, with each route typically offering access to only a single-ring system. Broader access to alkaloid-like chemical space is therefore highly desirable,^18,19^ and indeed access to such analogues has seen considerable recent interest.^20−24^ Biomimetic syntheses offer attractive approaches to such systems,^25,26^ with arguably the first example of this being Robinson’s synthesis of tropinone **2** (Scheme 1A), which achieved this via a one-step double-Mannich reaction.^27,28^ However, such an approach has yet to be extended more broadly, despite potentially enabling a general conversion of easily accessed dialdehydes into complex natural product-like scaffolds. We considered that stereochemically rich 1,4-dialdehydes of type **3**, which are readily available from Diels–Alder cycloaddition and oxidative cleavage sequences, might be attractive substrates in this respect. Indeed, such systems have seen use in reductive amination-based diversification to form systems of type **4** (Scheme 1B);^29,30^ however, their extension to double-Mannich reactions to form system **5** remains essentially unexplored.^31^ Overall, such an approach would represent a one-atom ring expansion of simple alkenes, forming systems that appear attractive as scaffolds for derivatization into compound libraries.

**Scheme 1:**
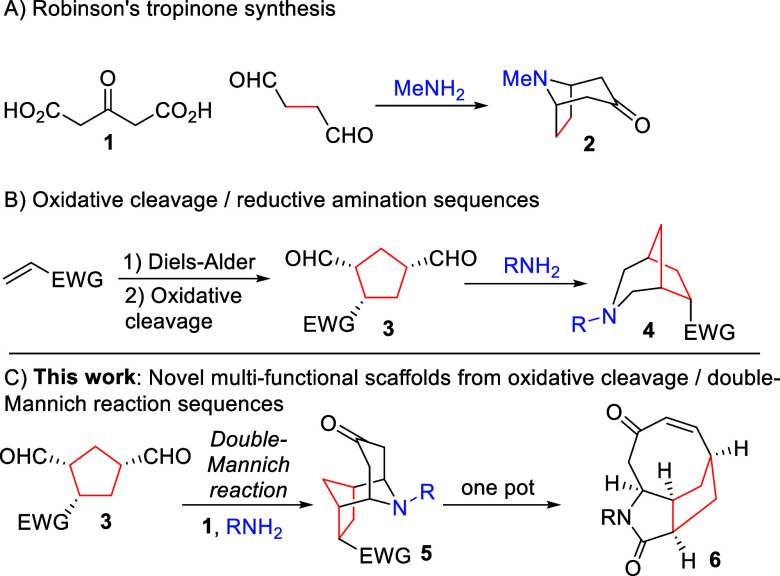
Previous and Current Access to 3D Amine Scaffolds from Dialdehyde Building Blocks

Herein, we show that a double-Mannich process involving dialdehyde **3** allows the stereoselective formation of tricyclic system **5** and that this undergoes efficient rearrangement to tricyclic lactam **6** (Scheme 1C).^32^

Our starting point was *tert*-butyl system **9a**, where we envisaged that the bulky ester would offer stereocontrol within the key enolate addition step, which in contrast to a reductive amination reaction forms two additional stereocenters.

Oxidative cleavage was attempted via both one- and two-step approaches; however, one-step approaches using either ozone or OsO_4_ in the presence of sodium periodate led to considerable loss of mass, apparently as a result of decomposition of the product. Gratifyingly, a two-step approach proved to be more successful, in which stable intermediate diol was directly reacted with sodium periodate to form dialdehyde **7**. Optimization permitted Os(VI) precatalyst loadings as low as 0.3 mol % with a quantitative yield over two steps. The resulting aldehyde was highly sensitive and thus used directly without purification.

While conditions for performing double-Mannich reactions on succinaldehyde are well-developed,^27^ such reactions are typically performed under aqueous conditions, which limits the solubility of hydrophobic species, such as compound **7**. We initially focused on the use of benzylamine as the amine partner (Table 1), which showed that, while use of water alone is possible (entry 1), scale-up became challenging as a result of a lack of homogeneity. We therefore undertook the small screen of organic co-solvents. Surprisingly, yields for all systems were high, and all reactions proceeded with high levels of stereoselectivity. However, several systems were far from homogeneous, leading to considerable issues with stirring. Moving to water–dioxane systems reduced this issue, resulting in a far greater ability to perform reaction scale-up. In all cases, essentially, a single diastereomer of product **8** was observed. The stereochemistry of the product was confirmed by single-crystal X-ray diffraction (XRD), demonstrating that attack of acetone-derived enolate on the intermediate iminium ion occurs from the opposite face to the bulky ester moiety.

**Table 1:**
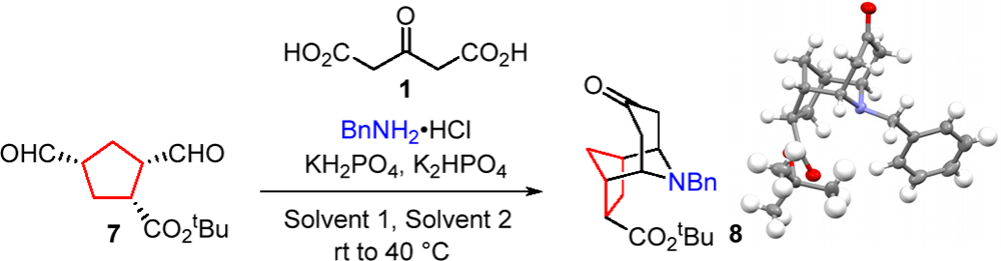
Optimization of the Double-Mannich Reaction

entry	solvent 1	solvent 2	yield (%)^a^^,^^b^
1	H_2_O	none	72^c^
2	H_2_O	THF	65^c^
3	THF	none	61^d^
4	H_2_O	1,4-dioxane	68
5	H_2_O	MeCN	69^c^
6	H_2_O	DMF	69

a All yields are given over three steps.

b Reactions were performed on a 1.3 mmol scale at a concentration of 0.1 M.

c The reaction scale was limited by precipitation of organic materials.

d The scale was limited by poor solubility of inorganic bases.

With optimal reaction conditions identified, we next studied the scope of the reaction. As shown in Scheme 2, the reaction tolerated variation of both norbornene and amine components, with the formation of *N*-PMB and *O*-benzyl systems **10b** and **10c** being particularly efficient. Indeed, the reactions forming compounds **10a**–**10c** proved scalable to multigram quantities with minimal reduction in the yield. However, use of simple methyl ester-functionalized norbornene led to lower yields of compound **10d**, in part because of the formation of more complex reaction mixtures, together with their greater water solubility, reducing product isolation in the intermediate oxidation steps. The inclusion of heterocyclic moieties was found to be tolerated across all three steps, as shown by the formation of pyridine **10e** and furan **10f**, albeit with some reduction in the yield. Importantly, both *endo* and *exo* norbornene stereoisomers were found to give opposite product diastereomers **10g** and **10h**, demonstrating the stereoretention of the process. Use of simple alkyl amines also proved viable, as shown in the formation of methyl amine system **10i**. Use of other activating moieties in the norbornene core again proved possible, with nitrile **10j** being formed, albeit in a reduced yield, again relating to low mass recoveries within the oxidation sequence. Overall, the process thus represents a direct and stereocontrolled transformation of simple norbornenes in complex polycyclic scaffolds.

**Scheme 2:**
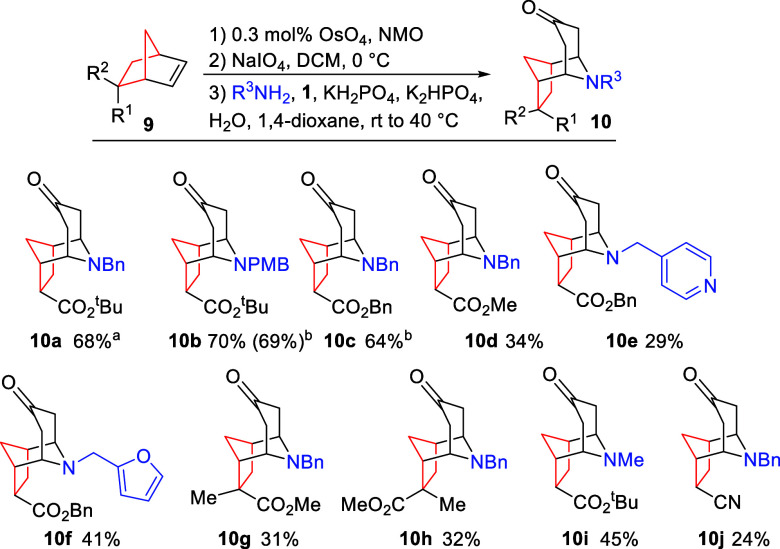
Scope of the Oxidative Cleavage/Double-Mannich Reaction Sequence Performed on a 5 g scale. Performed on a 3 g scale. All of the yields are isolated yields over three steps.

We next explored the derivatization of the products to enable a broad library synthesis. As shown in Scheme 3, deprotection of the *N*-benzyl moiety of compound **10a** is easily achieved, enabling efficient amide formation to form system **11**. The ester moiety was then easily functionalized, with a one-pot acid-promoted deprotection/amidation sequence forming diamide **12** in a good yield. Further, the ketone moiety of compound **10b** was stereoselectively reduced with NaBH_4_, with the incoming hydride being delivered from the same face as the amine. The resulting alcohol was then efficiently acetylated, providing a single diastereomer of compound **13**. The resulting compound underwent clean debenzylation via hydrogenolysis to form secondary amine **14**, which enabled direct access to the free amino acid via *tert*-butyl ester deprotection using trifluoroacetic acid (TFA) or conversion to the corresponding amide, as shown through the formation of compound **15**. In combination, these sequences demonstrate the potential of scaffold **10** to reliably form 3D compound libraries via standard amide formation processes as well as the potential to be incorporated into peptides as an unnatural amino acid residue.

**Scheme 3:**
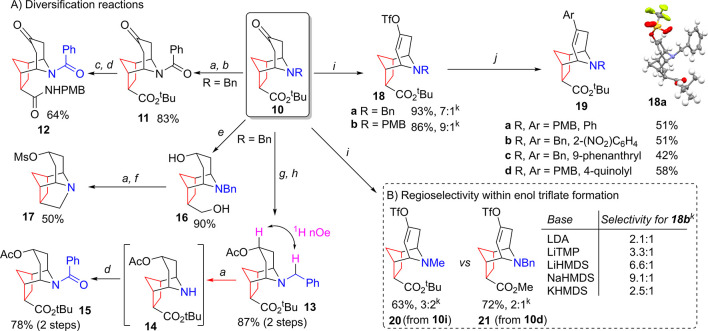
(A) Derivatization of Scaffold **10** and (B) Regioselectivity within Enol Triflate Formation Pd/C (10%), HCO_2_NH_4_, MeOH, and 60 °C. PhCOCl, ^i^Pr_2_NEt, DMAP, CH_2_Cl_2_, and 0 °C to room temperature (rt). TFA, CH_2_Cl_2_, and 0 °C and then oxalyl chloride, CH_2_Cl_2_, and 0 °C. PMBNH_2_, ^i^Pr_2_NEt, DMAP, CH_2_Cl_2_, and 0 °C to rt. LiAlH_4_, tetrahydrofuran (THF), and 0–60 °C. MsCl, ^i^Pr_2_NEt, and CH_2_Cl_2_. NaBH_4_, MeOH, H_2_O, and rt. Ac_2_O, ^i^Pr_2_NEt, DMAP, CH_2_Cl_2_, and 0 °C to rt. NaHMDS, THF, and −78 °C and then PhNTf_2_ and to rt. ArB(OH)_2_, Pd(PPh_3_)_4_, Na_2_CO_3_, LiCl, 1,4-dioxane, and 85 °C. Determined by ^1^H NMR analysis of the crude reaction product.

Having explored amidations and esterifications, we next investigated whether cyclization of the amine and ester moieties, which are clearly close in space, was possible. Gratifyingly, following reduction of both carbonyl functionalities with LiAlH_4_, direct hydrogenolysis and double mesylation of the crude diol allowed for cyclization to tetracycle **17**, which retains a single unreacted mesylate moiety. The ketone moiety also appeared to offer access to standard Pd-catalyzed diversification routes^33−35^ via conversion to the corresponding enol triflate. Pleasingly, despite concerns regarding the formation of regioisomeric mixtures during enol triflate formation, we observed some selectivity in the initial reactions. This was optimized though variation of the base (Scheme 3B), with NaHMDS proving optimal, giving access to both *N*-benzyl and *N*-PMB systems **18** with good levels of regiocontrol. This selectivity is surprising given the remote position of the ester moiety, which represents the sole point of asymmetry in the system. We therefore undertook variation of the substrate to probe this further. Interestingly, while switching from *N*-PMB to *N*-Bn was found to have relatively little impact (**18a** versus **18b**), moving to *N*-Me system **10i** led to a large decrease in selectivity in the formation of compound **20** (Scheme 3). Further, moving from *tert*-butyl ester **10a** to the simple methyl ester **10d** again gave a large decrease in the level of regiocontrol in the formation of compound **21**. Such observations are consistent with a relay of stereochemical information across the molecule via the nitrogen substituent, requiring bulk within both the N and ester substituents (see the Supporting Information for full details). This is also consistent with the behavior seen for compounds **10c** and **10j**, which gave low selectivity (<3:1) in addition to a significantly reduced yield.

Cross-coupling of enol triflates **18** also proved successful, with both *N*-benzyl and *N*-PMB systems undergoing Suzuki cross-coupling with a range of coupling partners, as shown by the formation of compound **19**. Such Pd-catalyzed processes offer reliable C–C bond formation and, in combination with the aforementioned functionalization and selective enol triflate formation, underline the versatility of such systems to function as tricyclic 3D scaffolds.

The previously observed cyclization to form mesylate **17** led us to consider whether such bond formation might permit an overall rearrangement within these scaffolds. To this end, we took PMB amine **10b** and performed ester deprotection followed by activation of the resulting carboxylic acid with thionyl chloride. Gratifyingly, this led to C–N formation with concurrent cleavage of the adjacent C–N bond (see the Supporting Information for the full mechanism), giving rise to a mixture of chloride **22** and alkene **23**. While chloride **22** could be isolated in moderate yield, inclusion of a 1,8-diazabicyclo[5.4.0]undec-7-ene (DBU)-mediated elimination as the final stage of this one-pot process allowed for the isolation of only alkene **23** in excellent yield in a single operation. This product again represents a novel and rigid scaffold, possessing an unusual eight-membered ring within a tricyclic framework.

Facile derivatization of this system also proved possible, as shown in Scheme 4. The addition of allymagnesium bromide proceeded in excellent yield to give a tertiary alcohol **24** as a 6:1 mixture of diastereomers. Somewhat counterintuitively, the addition preferentially occurs to the *endo* rather than *exo* face of the molecule; however, this can be rationalized by the relative flexibility of the eight-membered ring and the preference for orientation of the ketone carbonyl *anti* to the carbonyl of the amide. Reduction also proved possible, with transfer hydrogenation of alkene to form compound **25** being especially facile. This is consistent with alkene having limited conjugation with the adjacent carbonyl, which is apparent from both the alkenic chemical shifts within the ^1^H nuclear magnetic resonance (NMR) spectrum and the low reactivity of alkene in standard cycloaddition processes. Conversion of the ketone moiety to the corresponding enol triflate again proved to be facile, giving diene **26** in a good yield. This was found to undergo efficient Suzuki cross-coupling to form compound **27** as well as quantitative reduction via transfer hydrogenation to form parent system **28**.

**Scheme 4:**
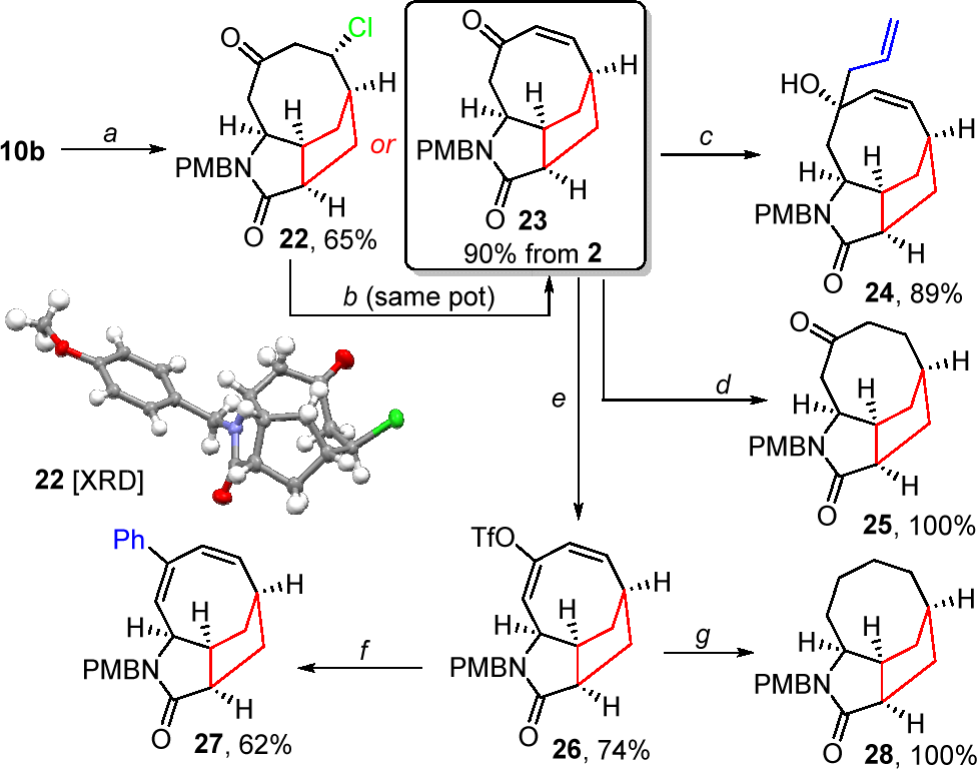
Direct Conversion to [8,5,5]-Fused System **23** TFA, CH_2_Cl_2_, and 0 °C to rt and then oxalyl chloride, CH_2_Cl_2_, and 0 °C to rt. DBU, MeCN, and 80 °C. AllylMgBr, THF, and 0 °C to rt. Pd/C (10%), HCO_2_NH_4_, MeOH, and 60 °C. NaHMDS, THF, and −78 °C and then PhNTf_2_ and to rt. ArB(OH)_2_, Pd(PPh_3_)_4_, Na_2_CO_3_, LiCl, 1,4-dioxane, and 85 °C. Pd/C (10%), HCO_2_NH_4_, MeOH, and 60 °C.

In conclusion, successive alkene oxidative cleavage and double-Mannich reaction sequences enable the stereoselective transformation of simple norbornenes into complex tricyclic alkaloid-like scaffolds. This represents a one-atom ring expansion with simultaneous annulation and permits direct and scalable synthesis of scaffolds that show 3-fold orthogonal reactivity. The compounds also undergo surprisingly regioselective enol triflate formation, with subsequent cross-coupling adding an additional scope for diversification. Further, the scaffold formed is readily converted into system **23**, which possesses an unusual 8,5,5-tricyclic architecture and undergoes similarly broad diversification. Given the high levels of diastereocontrol and that methods for performing asymmetric Diels–Alder reactions are well-established,^36−38^ the methodology also offers access to complex, enantioenriched scaffolds. Use of such oxidative cleavage/double-Mannich sequences does not need to be limited to norbornene-derived alkenes, and studies of the scope of this one-atom ring expansion/annulation methodology are underway.

## Data Availability

The data underlying this study are available in the published article and its Supporting Information.

## Supporting Information Available

Figures, tables, experimental details, and characterization data for all new compounds (PDF)
