# Supplementary material for: Stereoselective Access to Diverse Alkaloid-Like Scaffolds via an Oxidation/Double-Mannich Reaction Sequence
Source: Org Lett. 2024 Jun 21;26(26):5549–53. doi: 10.1021/acs.orglett.4c01924 (PMC11232018; doi:10.1021/acs.orglett.4c01924)
Supplement: Supplementary file 1 — ol4c01924_si_001.pdf [file ol4c01924_si_001.pdf]

Supporting Information

Stereoselective Access to Diverse Alkaloid-like Scaffolds via an Oxidation/Double-Mannich Reaction Sequence

Charles P. Mikan,[a] Joseph O. Watson,[a] Ryan Walton,[a] Paul G. Waddell[b] and Jonathan P. Knowles*[a]

jonathan.p.knowles@northumbria.ac.uk

[a] Department of Applied Sciences, Northumbria University, Ellison Place, Newcastle upon Tyne, UK

[b] School of Natural and Environmental Sciences, Newcastle University, Newcastle upon Tyne, UK

Table of contents

1. **Figures S1 and S2** 2
2. **General Experimental details** 3
3. **Synthesis of norbornene substrates** 3
4. **General procedures** 5
5. **Synthesis of compounds 10** 6
6. **Derivatization of scaffold 10 and synthesis of compounds 23-28** 12
7. **Crystallographic studies** 28
8. **References** 34
9. **NMR spectra** 35

1. Figures S1 and S2

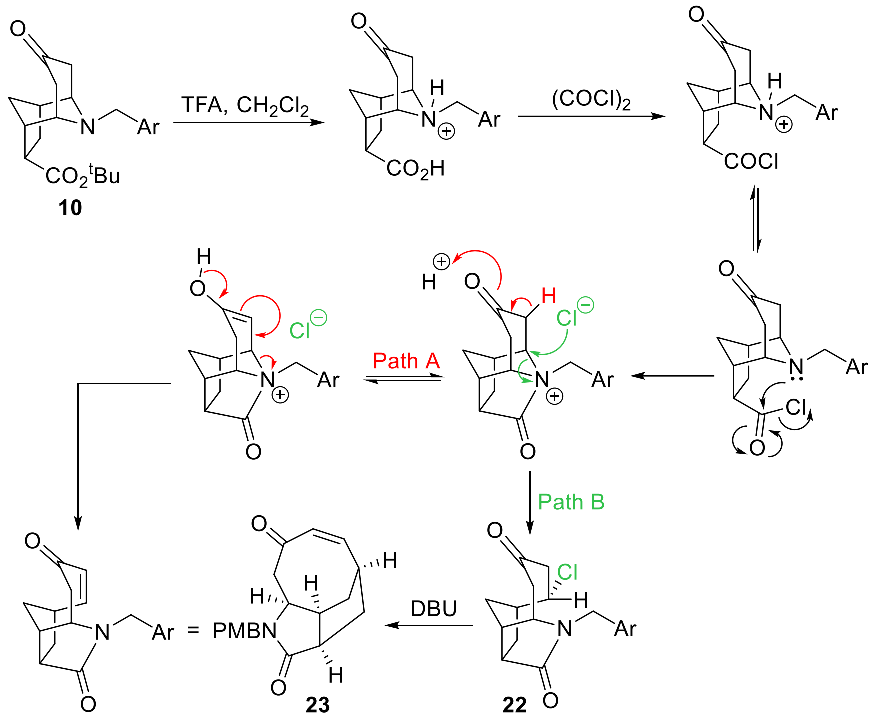

Figure S1: Proposed mechanism for the conversion of system **10** into **22** and **23**.

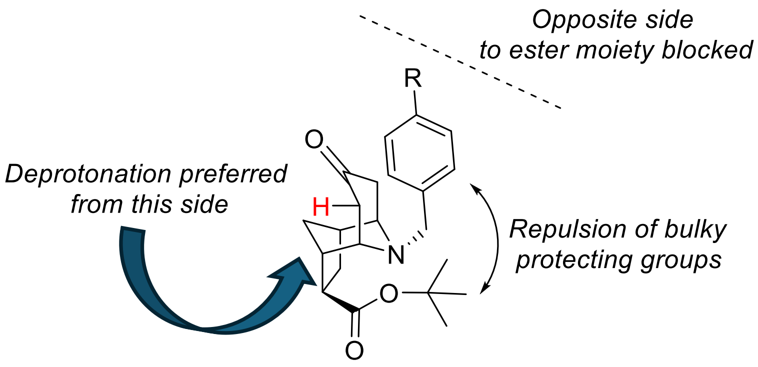

Figure S2: Proposed rationale of the regioselectivity observed in the formation of enol triflates **xx**.

2. General experimental details.

All palladium-catalysed processes were carried out using Schlenk technique under argon using commercially available anhydrous dioxane unless stated otherwise. Chemicals were obtained from commercial sources unless otherwise stated. THF and toluene were dried over 3 Å activated molecular sieves for 3 days prior to use. Where reactions required heating, this was achieved using an oil bath. Column chromatography was performed using 40-60 mesh silica powder. NMR spectroscopic analysis was performed using Jeol ECS 400 MHz instrument. Chemical shifts are reported in δ ppm. ^{13}C NMR are referenced to solvent as internal standard ($CDCl_3$ or DMSO). Data are reported as follows: chemical shift, multiplicity (s = singlet, d = doublet, t = triplet, q = quartet, p = pentent, dd = doublet of doublets, dt = doublet of triplets, td = triplet of doublets, tt = triplet of triplets, ddt = doublet of doublet of triplets, ddd = doublet of doublet of doublets, m = multiplet, app = apparent), coupling constants (Hz), and integration. Structural assignments were made with additional information from NOESY, COSY and gHSQC experiments. Mass spectrometry analysis was performed using electrospray ionisation with Orbitrap detection.

3. Synthesis of norbornene substrates.

Compound **S1**

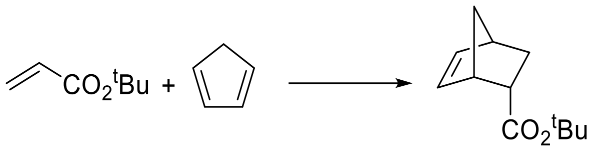

Anhydrous $ZnCl_2$ (1.20 g, 8.80 mmol) was dissolved with stirring in acetone (10 mL). Et_2O (90 mL) was added and the stirred solution cooled to 0 °C under nitrogen. *Tert*-butyl acrylate (6.80 mL, 46 mmol) was added, followed by freshly distilled cyclopentadiene (10.6 g, 161 mmol) dropwise. The reaction was allowed to warm to rt. After 6 days the reaction was washed with sat. aq. $NaHCO_3$ (60 mL). The aqueous phase was extracted with Et_2O (30 mL) and the combined organic phase dried ($MgSO_4$) and evaporated to give a clear oil. Purification by silica gel chromatography (Et_2O/petrol, 1% to 3% as eluent) afforded the title compound (7.43 g, 83%) as a clear oil. All spectral data was as previously reported.[1]

Compound **S2**

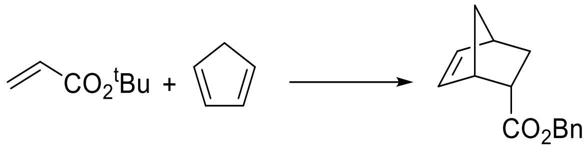

$ZnCl_2$ (0.89 g, 7 mmol) was dissolved in acetone (75 mL), Et_2O (67.5 mL) was added and the reaction was cooled to 0°C before the addition of benzyl acrylate (5 mL, 33 mmol). Freshly cracked cyclopentadiene (12 mL, 114 mmol) was added dropwise. The reaction was warmed to rt and stirred for 24h. The reaction was quenched using $NaHCO_3$ (60mL), the organic phase was dried over $MgSO_4$ and solvent removed under pressure. Purification by silica gel chromatography (2:98 to 4:96 Et_2O/petrol as eluent) afforded the title compound (6.283g, 83%) as a clear oil. All spectral data was in accord with that reported.[2]

Compound **S3**

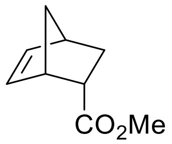

To a vigorously stirred solution of methyl acrylate (5.0 mL, 55 mmol) in water (55 mL) was added cyclopentadiene (6.8 mL, 82 mmol) dropwise. The mixture was stirred for 17 h. The mixture was extracted with DCM (2 × 50 mL), dried ($MgSO_4$) and evaporated to give crude clear oil (*endo*/*exo* 67:33). Purification by silica gel chromatography (Et_2O/petrol, 0:100 to 1:4 as eluent) afforded the *endo* product as a clear oil (2.4 g containing 5 mol% *exo*, 29%) and a mixture of *endo*/*exo* product (67:33) as a clear oil (4.8 g, 46%). Data was in accord with that reported.[3]

Compound **S4**

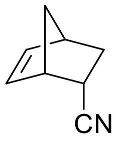

To stirred acrylonitrile (5.0 mL, 76 mmol) at 0 °C, cyclopentadiene (8.1 mL, 66 mmol) was added dropwise over 2 min. The mixture was allowed to reach rt and stirred for 3 days. The reaction was evaporated to give a crude oil (*endo*/*exo*; 61:39). Purification by silica gel chromatography (Et_2O/petrol, 0:100 to 3:7) afforded the products as a clear oils: *exo* (1.6 g, 17%); *endo* (3.0 g, 33%) and mixed *endo*/*exo* (3.0 g, 33 %). All spectral data was in accord with that reported.[4]

Compounds **S5** and **S6**

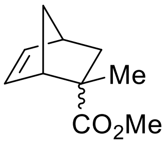

To a sealed tube, methyl methacrylate (2.0 mL, 19 mmol), cyclopentadiene (3.0 mL, 37 mmol) and toluene (1 mL) were sequentially added. The tube was sealed, heated to 100 °C and stirred for 27 h. The reaction cooled to rt and evaporated to give a crude oil (*endo*/*exo*; 1:2). Purification by silica gel chromatography (Et_2O/petrol, 0:100 to 8:92) afforded the products as a clear oils: *endo*-**S5** (300 mg, 10%); *exo*- **S6** (748 mg, 24%). All spectral data was in accord with that reported.[5,6]

4. General Procedures.

General procedure 1: Dihydroxylation/oxidative cleavage sequences.

To a solution of substrate (1 equiv.), NMO.H_2O (2 equiv.) and ^{t}BuOH (n µL) in dioxane (0.22 M) was added a solution of K_2OsO_4 (0.4 mol%) in water (14 M). Further water (0.5 M) was added and the reaction stirred at rt for 48 h. The reaction was quenched with aq. sodium metabisulfite (10% w/v), extracted and the combined organic phase was dried ($MgSO_4$) and evaporated. The crude mixture was filtered through a silica plug (EtOAc/petrol as eluent), the filtrate evaporated and the residue redissolved in DCM (n mL). The mixture was cooled to 0°C , water (n mL) was added, followed by sodium metaperiodate (n mmol). The reaction was warmed to rt, stirred for 2 h and the phases separated. The aqueous phase was extracted with DCM and the combined organic phase dried ($MgSO_4$) and evaporated to give the crude dialdehyde which was used immediately.

General procedure 2: Double-Mannich reactions.

To a solution of K_2HPO_4 (1.7 equiv.), KH_2PO_4 (0.7 equiv.), and acetone-1,3-diacid (2.3 equiv.) in H_2O (0.1 M) was added benzylamine hydrochloride solution (2 equiv. as a 2 M aq. solution). Substrate (1 equiv.) in dioxane (0.5 M) was added and the reaction stirred at rt for 24 h. The reaction was heated to 45 °C for a further 24 h, cooled and the mixture extracted with DCM. The combined organic phase was dried ($MgSO_4$) and evaporated. Purification by silica gel chromatography afforded the title compound.

5. Syntheses of compounds 10

Compound **10a**

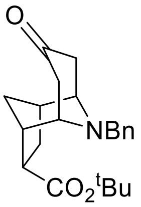

From norbornene **S1** (3.56 g, 18.4 mmol) following General Procedure 1 to give crude dialdehyde (4.19 g, 100%). General Procedure 2 then afforded the title compound as a white solid (4.84 g, 68%) after silica gel chromatography (EtOAc/petrol, 1:4 as eluent). Mp. 122 – 123 °C. ν_{max} /cm^{-1} (film) 2936, 1703, 1455, 1366 and 1158; ^{1}H NMR ($CDCl_3$, 400 MHz): δ 1.27 (dt, 1H, J = 13.4, 4.4 Hz, $CHCH\mathit{H}CH$), 1.50 (s, 9H, $C\mathit{Me}_3$), 1.68 (dd, 1H, J = 13.3, 2.7 Hz, $CHC\mathit{H}HCH$), 1.77 (td, 1H, J =12.2, 6.2 Hz, $CH\mathit{H}CHCO_2{}^tBu$), 1.96 – 2.04 (m, 1H, $C\mathit{H}CH_2CHCO_2{}^tBu$), 2.12 (d, 1H, J = 17.9 Hz, $CH\mathit{H}CHCHCH_2CO_2{}^tBu$), 2.22 (ddd, 1H, J = 12.6, 5.0, 2.5 Hz, $C\mathit{H}HCHCO_2{}^tBu$), 2.33 (d, 1H, J = 18.2 Hz, $CH\mathit{H}CHCHCHCO_2{}^tBu$), 2.44 (app. q, 1H, J = 4.7 Hz, $C\mathit{H}CHCO_2{}^tBu$), 2.52 (dd, 1H, J = 18.2, 6.7 Hz, $C\mathit{H}HCHCHCH_2CO_2{}^tBu$), 2.71 (dd, 1H, J = 18.2, 7.0 Hz, $C\mathit{H}HCHCHCHCO_2{}^tBu$), 2.74 – 2.83 (m, 2H, $NC\mathit{H}CHCH_2CHCO_2tBu$ and $C\mathit{H}CO_2tBu$), 3.63 (app. t, 1H, J = 5.4 Hz, $NC\mathit{H}CHCHCO_2{}^tBu$), 3.64 – 3.73 (AB-q, 2H, $NC\mathit{H}_2Ph$), 7.23 – 7.29 (m, 1H, $C\mathit{H}_{Ar}$), 7.33 (app. t, 2H, J = 7.4 Hz, 2 × $C\mathit{H}_{Ar}$), 7.45 (app. t, 2H, J = 7.4 Hz, 2 × $C\mathit{H}_{Ar}$). $^{13}C\{^1H\}$ NMR ($CDCl_3$, 101 MHz): δ 28.4 ($C\mathit{Me}_3$), 29.1 ($\mathit{C}H_2CHCHCO_2{}^tBu$), 31.0 ($\mathit{C}H_2CHCO_2{}^tBu$), 38.6 ($\mathit{C}H_2CHCHCH_2CHCO_2{}^tBu$), 39.2 ($\mathit{C}H_2CHCHCHCO_2{}^tBu$), 40.4 ($\mathit{C}HCH_2CHCO_2{}^tBu$), 44.1 ($\mathit{C}HCHCO_2{}^tBu$), 47.7 ($\mathit{C}HCO_2{}^tBu$), 54.7 ($N\mathit{C}HCHCH_2CHCO_2{}^tBu$), 56.3 ($N\mathit{C}H_2Ph$), 56.7 ($N\mathit{C}HCHCHCO_2{}^tBu$), 80.1 ($\mathit{C}Me_3$), 127.5 ($\mathit{C}H_{Ar}$), 128.6 (2 × $\mathit{C}H_{Ar}$), 129.3 (2 × $\mathit{C}H_{Ar}$), 138.1 (C_{qAr}), 173.6 ($\mathit{C}O_2{}^tBu$), 211.0 (ketone). HRMS (ESI$^+$-Orbitrap) m/z: $[M+H]^+$ Calcd for $C_{22}H_{30}NO_3$ 356.2226; Found 356.2228.

Compound 10b

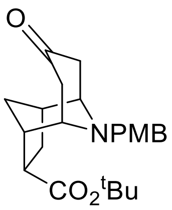

From norbornene **S1** (3.00 g, 15.5 mmol) following General Procedure 1 to give crude dialdehyde (3.66 g, 100%). Following General Procedure 2 afforded the title compound **9a** as a white solid (2.94 g, 69%) after silica gel chromatography (EtOAc/petrol, 15 :85 to 3:7 as eluent). Mp. 138 – 139 °C. ^{1}H NMR ($CDCl_3$, 400 MHz): δ 1.28 (dt, 1H, J = 14.1, 4.3 Hz, $CHCH\mathit{H}CH$), 1.51 (s, 9H, $C\mathit{Me}_3$), 1.68 (app. d, 1H,

app. J = 13.3 Hz, CHC*H*HCH), 1.76 (td, 1H, J = 12.1, 6.1 Hz, CH*H*CHCO$_2$^{t}Bu), 1.97 – 2.04 (m, 1H, C*H*CH$_2$CHCO$_2$^{t}Bu), 2.12 (d, 1H, J 17.7 Hz, CH*H*CHCHCH$_2$CO$_2$^{t}Bu) 2.20 (dd, 1H, J = 13.6, 4.0 Hz, C*H*HCHCO$_2$^{t}Bu), 2.28 – 2.36 (m, 1H, CH*H*CHCHCHCO$_2$^{t}Bu), 2.40 – 2.46 (m, 1H, C*H*CHCO$_2$^{t}Bu), 2.51 (dd, 1H, J = 17.8, 1.8 Hz, C*H*HCHCHCH$_2$CO$_2$^{t}Bu), 2.69 (dd, 1H, J = 17.8, 7.0 Hz, C*H*HCHCHCHCO$_2$^{t}Bu), 2.74 – 2.82 (m, 2H, NC*H*CHCH$_2$CHCO$_2$tBu and C*H*CO$_2$tBu), 3.57 – 3.67 (m, 3H, NC*H*CHCHCO$_2$^{t}Bu and NC*H*$_2$Ar), 3.79 (s, 3H, OMe), 6.89 (d, 2H, J = 8.2 Hz, 2 × C*H*$_{Ar}$), 7.36 (d, 2H, J = 8.3 Hz, 2 × C*H*$_{Ar}$). ^{13}C{^{1}H} NMR (CDCl$_3$, 101 MHz): δ 28.5, 29.2, 31.0, 38.6, 39.2, 40.4, 44.2, 47.8, 54.5, 55.4, 57.7, 80.0, 113.9, 130.1, 130.3, 159.0, 173.6, 211.1. HRMS (ESI$^+$-Orbitrap) m/z: [M+H]$^+$ Calcd for $C_{23}H_{32}NO_4$ 386.2332; Found 386.2335.

Compound **10c**

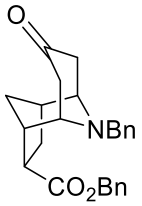

From norbornene **S2** (4.00 g, 17.5 mmol) following General Procedures 1 and 2 to afford the title compound **9c** as a white solid (3.17 g, 64%) after silica gel chromatography (EtOAc/petrol, 15:85 as eluent). Mp. 102 – 104 °C. ν_{max} /cm^{-1} (film) 2940, 1724, 1698, 1496, 1454 and 1199; ^{1}H NMR (CDCl$_3$, 400 MHz): δ 1.28 (dt, 1H, J = 13.2, 4.0 Hz, CHCH*H*CH), 1.69 (dd, 1H, J = 13.3, 2.5 Hz, CHC*H*HCH), 1.87 (td, 1H, J = 12.1, 6.1 Hz, CH*H*CHCO$_2$Bn), 2.03 (dd, 1H, J = 6.3, 4.0 Hz, C*H*CH$_2$CHCO$_2$Bn), 2.15 (d, 1H, J = 18.0 Hz, CH*H*CHCHCH$_2$CO$_2$Bn), 2.23 – 2.34 (m, 2H, C*H*HCHCO$_2$Bn and CH*H*CHCHCHCO$_2$Bn), 2.48 (app. q, 1H, J = 4.8 Hz, C*H*HCHCHCH$_2$CO$_2$Bn), 2.56 (dd, 1H, J 18.1, 6.7 Hz, C*H*HCHCHCHCO$_2$Bn), 2.64 (dd, 1H, J = 18.2, 7.0 Hz, C*H*HCHCHCHCO$_2$Bn), 2.80 – 2.90 (m, 2H, NC*H*CHCH$_2$CHCO$_2$Bn and C*H*CO$_2$Bn), 3.41 (d, 1H, J = 12.7 Hz, NCH*H*Ph), 3.49 (t, 1H, J = 5.9 Hz, NC*H*CHCHCO$_2$Bn), 3.58 (d, 1H, J = 12.8, NC*H*HPh), 5.08 (s, 2H), 7.22 – 7.38 (m, 10H, 2 × Ph). ^{13}C{^{1}H} NMR (CDCl$_3$, 101 MHz): δ 29.0, 31.3, 38.8, 40.4, 43.7, 46.6, 55.4, 56.1, 66.2, 127.5, 128.3, 128.5, 128.6, 128.7, 129.4, 136.3, 138.0, 174.0, 210.7. HRMS (ESI$^+$-Orbitrap) m/z: [M+H]$^+$ Calcd for $C_{25}H_{29}NO_3$ 390.2069; Found 390.2069.

Compound (±)-10d

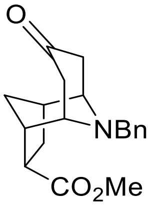

From norbornene **S3** (1.2 g, 7.9 mmol) following General Procedures 1 and 2 afforded the title compound (0.85 g, 34% over 3 steps) as a clear oil after silica gel chromatography (EtOAc/petrol, 1:9 to 3:7 as eluent). ν_{max} /cm^{-1} (neat) 2982, 2948, 2920, 2890, 1714, 1701, 1497, 1438. ^{1}H NMR ($CDCl_3$, 400 MHz): δ 1.29 (dt, 1H, J = 13.3, 4.1 Hz, CHC*H*HCH), 1.70 (dd, 1H, J = 13.2, 2.6 Hz, $MeCO_2$CHCH*H*), 1.86 (td, 1H, J = 12.1, 6.1 Hz, CHCH*H*CH), 2.07 (app. q, 1H, J = 5.1 Hz, $CHCH_2$C*H*), 2.18 (d, 1H, J = 18.0 Hz, CH*H*C=O), 2.25 (ddd, 1H, J = 12.5, 4.9, 2.6 Hz, CHCH*H*CH), 2.32 (d, 1H, J = 18.3 Hz, CH*H*C=O), 2.46 (app. q, 1H, J = 4.9 Hz, MeO_2C*H*), 2.59 (dd, 1H, J = 18.1, 6.6 Hz, CH*H*C=O), 2.70 (dd, 1H, J = 18.2, 7.0 Hz, CH*H*C=O), 2.81 (dt, 1H, J = 11.1, 5.2 Hz, MeO_2CCHC*H*), 2.89 (app. t, 1H, J = 5.7 Hz, CH_2CHC*H*N), 3.53 (app. t, 1H, J = 5.9 Hz, MeO_2CCHC*H*N), 3.58 – 3.72 (m, 5H, NCH_2Ph and CO_2*Me*), 7.21 – 7.36 (m, 5H, 5 × CH_{Ar}). ^{13}C{^{1}H} NMR ($CDCl_3$, 101 MHz): δ 29.0 (MeO_2CCHCH_2CH), 31.3 (CHCH_2CH), 38.8 and 38.9 (CH_2COCH_2), 40.5 (MeO_2CCH), 46.4 (MeO_2CCHCHCHN), 51.4 (CO_2*Me*), 55.3 (MeO_2CCHCHCHN), 56.4 (CH_2CHCHN), 56.5 (NCH_2Ph), 127.5 (CH_{Ar}) 129.4 (CH_{Ar}), 138.0 (C_{qAr}), 174.5 (CO_2Me) and 210.8 (CH_2COCH_2). HRMS (ESI$^+$-Orbitrap) m/z: $[M+H]^+$ Calcd for $C_{19}H_{24}NO_3$ 314.1756; Found 314.1751.

Compound (±)-10e

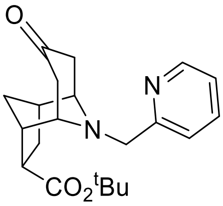

From norbornene **S1** (0.43 g, 1.9 mmol) following General Procedures 1 and 2. Purification by silica gel chromatography (EtOAc/petrol containing 1% Et_3N, 3:7 to 1:1 as eluent) afforded the title compound as a white solid (192 mg, 29% over three steps). Mpt. 131 – 140 °C. ν_{max} /cm^{-1} (neat) 2974, 2936, 2890, 1701, 1590, 1477, 1434 and 1365. ^{1}H NMR ($CDCl_3$, 400 MHz): δ 1.28 (dt, 1H, J = 13.7, 4.3 Hz, CHCH*H*CH), 1.48 (s, 9H, $CO_2C(Me)_3$), 1.70 (dd, 1H, J = 13.4, 2.6 Hz, CHCH*H*CH), 1.79 (td, 1H, J = 12.1, 6.0 Hz, CH*H*CHCO$_2{}^t$Bu), 2.03 (br s, 1H, CH(CO_2)CHCH_2C*H*), 2.13 (dt, 1H, J = 16.5, 3.7 Hz, CH_2COCH*H*), 2.23 (ddd, 1H, J = 12.8, 4.9, 2.5 Hz, C*H*HCHCO$_2{}^t$Bu), 2.34 (d, 1H, J = 18.0 Hz, CH*H*COCH_2), 2.45 (app. q, 1H, J = 5.0 Hz, C*H*(CO_2)), 2.65 – 2.77 (m, 3H, CH*H*COCH*H* and

CH_2CHCHN), 2.81 (dt, 1H, J = 11.1, 5.2 Hz, CH(CO_2)C*H*CH_2), 3.59 (app. t, 1H, J = 5.8 Hz, CH(CO_2)CHC*H*N), 3.73 (d, 1H, J = 13.6 Hz, NCH*H*Pyr), 3.98 (d, 1H, J = 13.5 Hz, NCH*H*Pyr), 7.16 (t, 1H, J = 6.3 Hz, CH_{Ar}), 7.74 (td, 1H, J = 7.8, 1.9 Hz, CH_{Ar}), 7.84 (d, 1H, J = 7.8 Hz, CH_{Ar}), 8.42 (d, 1H, J = 5.0 Hz, CH_{Ar}). $^{13}C\{^1H\}$ NMR ($CDCl_3$, 101 MHz): δ 28.4 ($CO_2C(Me)_3$), 29.1 (CHCH*H*CH), 31.0 (*C*H_2CH(CO_2)), 38.7 (*C*H_2CO*C*H_2), 39.4 (*C*H_2CO*C*H_2), 40.4 (CH(CO_2)CH_2*C*HCH_2), 47.7 (CH(CO_2)*C*H), 55.7 (CH_2CH*C*HN), 57.2 (CH(CO_2)CH*C*HN), 58.7 (N*C*H_2Pyr), 80.1 (CO_2*C*(Me)$_3$), 122.5 (*C*H_{Ar}), 124.0 (*C*H_{Ar}), 137.0 (*C*H_{Ar}), 149.0 (*C*H_{Ar}), 158.8 (C_{qAr}), 173.6 (*C*O_2), 210.5 (CH_2*C*OCH_2). HRMS (ESI^+-Orbitrap) m/z: $[M+H]^+$ Calcd for $C_{21}H_{28}N_2O_3$ 357.2179; Found 357.2174.

Compound (±)-10f

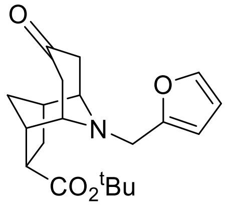

From norbornene **S1** (0.43 g, 1.9 mmol) following General Procedures 1 and 2. Purification by silica gel chromatography (EtOAc/petrol, 1:9 to 3:7 as eluent) afforded the product as a white solid (264 mg, 41% over three steps). Mp. 118 – 120 °C. ν_{max}/cm^{-1} (neat) 2975, 2935, 2889, 1701, 1479, 1455 and 1364. ^1H NMR ($CDCl_3$, 400 MHz): δ 1.26 (dt, 1H, J = 13.0, 4.0 Hz, CHCH*H*CH), 1.48 (s, 9H, $CO_2C(Me)_3$), 1.66 (dd, 1H, J = 13.8, 2.5 Hz, CHC*H*HCH), 1.79 (td, 1H, J = 12.0, 6.1 Hz, CH*H*CHC$O_2{}^t$Bu), 2.03 (app. q, 1H, J = 5.0 Hz, CH(CO_2)CHCH_2C*H*), 2.13 – 2.22 (m, 2H, C*H*HCHC$O_2{}^t$Bu and CH_2COCH*H*), 2.39 (app. q, 1H, J = 5.0 Hz, C*H*CO_2), 2.58 (m, 2H), 2.75 (dt, 1H, J = 11.1, 5.2 Hz, CH(CO_2)C*H*CH_2), 2.88 (app. t, 1H, J = 5.5 Hz, CH(CO_2)C*H*), 3.53 – 3.64 (m, 2H CH(CO_2)CHC*H*N and NCH*H*Fur), 3.83 (d, 1H, J = 14.1 Hz, NCH*H*Fur), 6.31 – 6.36 (m, 2H, 2 × CH_{Ar}), 7.33 (1H, d, J = 1.7 Hz, CH_{Ar}). $^{13}C\{^1H\}$ NMR ($CDCl_3$, 101 MHz): δ 28.3 (C(*Me*)$_3$, 28.9 (CH*C*HHCH), 31.3 (*C*H_2CH(CO_2)), 38.8, (*C*H_2CO*C*H_2), 39.1 (*C*H_2CO*C*H_2), 40.5 (CH(CO_2)CH_2*C*HCH_2), 43.7 (CH(CO_2)*C*H), 47.6 (CH(CO_2)*C*H), 49.0 (N*C*H_2Fur), 56.0 ((CH(CO_2)CH*C*HN and CH_2CH*C*HN – *overlap of two environments*), 80.0 (CO_2*C*(Me)$_3$), 108.6 (*C*H_{Ar}), 110.6 (*C*H_{Ar}), 141.8 (*C*H_{Ar}), 152.3 (C_{qAr}), 173.4 (*C*$O_2{}^t$Bu), 210.8 (CH_2*C*OCH_2). HRMS (ESI^+-Orbitrap) m/z: $[M+H]^+$ Calcd for $C_{20}H_{27}NO_4$ 346.2019; Found 346.2013.

Compound (±)-10g

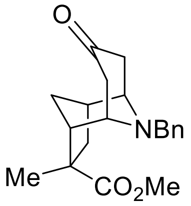

From *endo*-norbornene **S5** (101 mg, 0.61 mmol) following General Procedures 1 and 2 afforded the title compound as a clear oil (62 mg, 31% over 3 steps) after silica gel chromatography (EtOAc/petrol, 2:98 to 1:4 as eluent). ν_{max}/cm^{-1} (neat) 2932, 1725, 1702, 1495, 1455, 1365, 1278. ^{1}H NMR ($CDCl_3$, 400 MHz): δ 1.28 (s, 3H, *Me*), 1.41 (dd, 1H, J = 12.7, 5.9 Hz, CHCH*H*CH), 1.48 – 1.63 (m, 2H, (Me)$MeO_2CC_qCH_2$), 2.02 – 2.09 (m, 1H, C*H*CH_2C*H*), 2.13 (d, 1H, J = 18.0 Hz, CH_2COCH*H*), 2.30 (d, 1H, J = 18.2 Hz, CH*H*$COCH_2$), 2.49 – 2.57 (m, 2H, CHCH*H*CH and CH_2COCH*H*), 2.68 (dd, 1H, J = 18.2, 7.0 Hz, CH*H*$COCH_2$), 2.83 (t, 1H, J = 5.7 Hz, (Me)MeO_2CC_qCH), 3.61 – 3.70 (m, 6H, NCH_2Ph and CO_2Me and (Me)C_qCHC*H*N), 7.24 – 7.43 (m, 5H, 5 × CH_{Ar}). ^{13}C{^{1}H} NMR ($CDCl_3$, 101 MHz): δ 26.3 ((Me)C_qCCH_2), 29.0 (*Me*CCO_2Me), 38.6, (*C*H_2CO*C*H_2) 38.9 (*C*H_2CO*C*H_2), 40.2 (CH*C*H_2CH), 41.4 (CHCH_2*C*H), 50.4 ((Me)C_q*C*H), 51.7 (CO_2Me), 55.9 ((Me)C_qCH*C*HN), 56.2 (N*C*H_2Ph), 127.5 (*C*H_{Ar}), 128.6 (*C*H_{Ar}), 129.2 (*C*H_{Ar}), 137.9 (C_{qAr}), 178.2 (*C*O_2Me), 210.9 (CH_2*CO*CH_2). HRMS (ESI$^+$-Orbitrap) m/z: [M+H]$^+$ Calcd for $C_{20}H_{25}NO_3$ 328.1914; Found 328.1908.

Compound (±)-10h

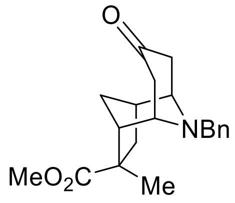

From *exo*-norbornene **S6** (106 mg, 0.64 mmol) following General Procedures 1 and 2 afforded the title compound as a clear oil (67 mg, 32% over 3 steps) after silica gel chromatography (EtOAc/petrol, 2:98 to 1:4 as eluent). ν_{max}/cm^{-1} (neat) 2946, 1724, 1702, 1454, 1434, 1364, 1265. ^{1}H NMR ($CDCl_3$, 400 MHz): δ 1.15 – 1.25 (m, 1H, (Me)C_qCH*H*), 1.48 – 1.69 (m, 5H, *Me*, (Me)C_qCH*H* and CHCH*H*CH), 2.07 – 2.13 (m, 1H, CHCH_2C*H*), 2.21 (d, 1H, J = 18.4 Hz, CH_2COCH*H*), 2.27 – 2.36 (m, 2H, CHCH*H*CH and CH*H*$COCH_2$) 2.43 (dd, 1H, J = 12.8, 6.5 Hz, CH*H*$COCH_2$), 2.59 (dd, 1H, J = 18.3, 6.8 Hz, CH_2COCH*H*), 3.31 (t, 1H, J = 5.6 Hz, (Me)C_qCHC*H*N), 3.63 – 3.77 (m, 5H, NCH_2Ph and CO_2Me), 7.19 – 7.50 (5H, m, 5 × CH_{Ar}). ^{13}C{^{1}H} NMR ($CDCl_3$, 101 MHz): δ 19.6 (*Me*), 28.2 ((Me)C_qCH_2), 38.5 (*C*H_2COCH_2), 39.2 (*C*H_2COCH_2 and CH*C*H_2CH), 41.4 (MeC_qCHCH_2*C*H), CH_2CH*C*HN), 52.3 (CO_2Me); 54.6 (MeC_q*C*H), 55.2 (MeC_qCH*C*HN), 56.0 (N*C*H_2Ph), 127.7 (*C*H_{Ar}),

128.6 (CH_{Ar}), 129.4 (CH_{Ar}), 137.8 (C_{qAr}), 179.7 (CO_2Me), 210.8 (CH_2COCH_2). HRMS (ESI$^+$-Orbitrap) m/z: $[M+H]^+$ Calcd for for $C_{20}H_{25}NO_3$ 328.1914; Found 329.1907.

Compound (±)-10i

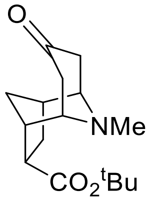

From norbornene **S1** (0.43 g, 1.9 mmol) following General Procedures 1 and 2. Purification by silica gel chromatography (Et_2O/DCM containing 1% Et_3N, 1:4 as eluent) afforded the product as a white solid (235 mg, 45% over three steps). Mpt. 85 – 88 °C ν_{max}/cm^{-1} (neat) 2972, 2933, 2892, 2810, 2776, 1700, and 1366. ^{1}H NMR ($CDCl_3$, 400 MHz): δ 1.22 (dt, 1H, J = 13.3, 4.2 Hz, CHCH*H*CH), 1.46 (s, 9H, $CO_2C(Me)_3$), 1.61 (dd, 1H, J = 13.8, 2.6 Hz, CHCH*H*CH), 1.79 (td, 1H, J = 12.0, 6.1 Hz, CH*H*CH(CO_2)), 2.07 (app. q, 1H, J = 5.0 Hz, CH(CO_2)CHCH_2C*H*), 2.14 – 2.21 (m, 2H, CH*H*CH(CO_2) and CH_2COCH*H*), 2.22 (d, 1H, J = 5.7 Hz, CH_2COCH*H*), 2.34 (m, 4H, N*Me* and C*H*(CO_2)), 2.59 (m, 2H, CH*H*COCH*H*), 2.71 (dt, 1H, J = 11.0, 5.1 Hz, CH(CO_2)C*H*), 2.95 (app. t, 1H, J = 5.6 Hz, CH_2CHC*H*N). ^{13}C{^{1}H} NMR ($CDCl_3$, 101 MHz): δ 28.3 (CH*C*HHCH), 31.9 (*C*HHCH(CO_2)), 38.2, 38.9 (*C*H_2CO*C*H_2), 39.8 (N*Me*), 40.8 (CH(CO_2)CH_2*C*HCH_2), 43.5 (*C*H(CO_2), 47.5 (CH(CO_2)*C*H), 55.9 (CH(CO_2)CH*C*HN), 60.8 (CH_2CH*C*HN), 79.8 (CO_2*C*(Me)$_3$), 173.6 (*C*$O_2{}^t$Bu), 211.0 (CH_2*C*OCH_2). HRMS (ESI$^+$-Orbitrap) m/z: $[M+H]^+$ Calcd for $C_{16}H_{25}NO_3$ 280.1914; Found 280.1906.

Compound (±)-10j

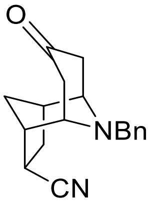

From norbornene **S4** (198 mg, 1.7 mmol) following General Procedures 1 and 2 afforded the title compound as a clear oil (72 mg, 24% over 3 steps) after silica gel chromatography ($CHCl_3$/petrol, 9:1 to 100:0 then Et_2O/$CHCl_3$, 1:99 to 5:95 as eluent). ν_{max}/cm^{-1} (neat) 3028, 2941, 2896, 2858, 2831, 2234, 1700, 1495, 1445). ^{1}H NMR ($CDCl_3$, 400 MHz): δ 1.22 (dt, 1H, J = 13.9, 4.2 Hz, CHCH*H*CH), 1.74 (d, 1H, J = 13.9 Hz, CHCH*H*CH), 1.90 – 2.03 (m, 2H, (NC)CHCHCH_2C*H*), 2.20 (d, 1H, J = 17.9 Hz, CH*H*COCH_2), 2.46 (app. q, 1H, J = 5.0 Hz, (NC)C*H*), 2.64 (dd, 1H, J = 17.9, 6.8 Hz, CH*H*COCH_2),

2.74 – 2.87 (m, 3H, NCCHCH*H* and CH_2CHC*H*N and CH*H*COCH_2), 3.61 (t, 1H, J = 5.8 Hz, NCCHCHC*H*N), 3.72 (d, 1H, J = 12.4 Hz, NCH*H*Ph), 3.89 (d, 1H, J = 12.4 Hz, NCH*H*Ph), 7.22 – 7.56 (m, 5H, 5 × C*H*$_{Ar}$). $^{13}C\{^1H\}$ NMR ($CDCl_3$, 101 MHz): δ 28.1 (NCCHCH_2), 30.6 (NCCHCH), 33.9 (CHCH_2CH), 38.4 (CH_2COCH_2), 38.9 (CH_2COCH_2), 40.5 (CHCH_2CH), 44.1 (NCCH), 53.9 (CH_2CHCHN), 55.9 (NCH_2Ph), 57.2 (NCCHCHCHN), 122.6 (*C*N), 127.2 (*C*H$_{Ar}$), 128.6 (*C*H$_{Ar}$), 129.8 (*C*H$_{Ar}$), 127.8 (*C*$_{qAr}$), 209.8 (CH_2*C*O). HRMS (ESI$^+$-Orbitrap) m/z: $[M+H]^+$ Calcd for $C_{18}H_{21}N_2O$ 281.1654; Found 281.1647.

6. Derivatization of scaffold 10 and synthesis of compound 23

Compound (±)-11

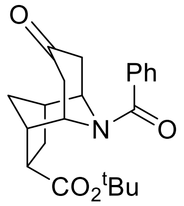

A stirred suspension of substrate **10a** (200 mg, 0.563 mmol) in MeOH (10 mL) was degassed by sparging with nitrogen and the mixture heated to 60 °C. Ammonium formate (172 mg, 2.73 mmol) and Pd/C (10 wt%, 70 mg, 12 mol%) were added and the reaction stirred at reflux. After 90 min the reaction was cooled, diluted with DCM (25 mL) and filtered through Celite. Evaporation gave a clear oil which was dissolved in DCM (10 mL) and cooled to 0 °C under nitrogen. DMAP (10 mg, 0.082 mmol) and $^iPr_2NEt$ (0.30 mL, 1.7 mmol) were added to the stirred solution followed by the dropwise addition of benzoyl chloride (0.13 mL, 1.1 mmol). The reaction was allowed to warm to rt. After 15 h the reaction was quenched by the addition of sat. aq. $NaHCO_3$ (25 mL) and extracted with DCM (2 × 20 mL). The combined organic phase was dried ($MgSO_4$) and evaporated to give the crude product as a yellow oil. Purification by silica gel chromatography (EtOAc/petrol, 3:7 to 1:1 as eluent) afforded the title compound (172 mg, 83% over two steps) as a white solid. Mp. 153 – 156 °C. ν_{max} /cm^{-1} 2975, 2892, 1701, 1636, 1601, 1578, 1405, 1156, 1134. ^1H NMR ($CDCl_3$, 400 MHz): δ 1.43 (dt, 1H, J = 13.7, 4.5 Hz, CH*H*), 1.48 (s, 9H, C*Me*$_3$), 1.85 (dd, 1H, J = 13.3, 2.6 Hz, C*H*H), 1.93 (ddd, 1H, J = 6.9, 6.2, 5.8 Hz, CH*H*), 2.19 (app. q, 1H, J = 4.9 Hz, CH), 2.78 (dd, 1H, J = 17.7, 6.6 Hz, CH*H*), 2.32 – 2.40 (m, 2H, C*H*H and C*H*H), 2.52 (d, 1H, J = 17.9 Hz, 1H, CH*H*), 2.63 (app. q, 1H, J = 4.9 Hz, CH), 2.87 – 2.99 (m, 2H, C*H*H and CH), 3.91 (t, 1H, J = 5.6 Hz, CH), 5.00 (t, 1H, J = 5.8 Hz, CH), 7.39 – 7.44 (m, 3H), 7.49 – 7.53 (m, 2H). $^{13}C\{^1H\}$ NMR ($CDCl_3$, 101 MHz): δ 28.4, 29.6, 29.9, 39.3, 42.9, 43.7, 45.4, 47.2, 47.4, 56.2, 81.5, 126.6, 128.9, 129.8, 135.7, 171.2, 171.9, 208.3. HRMS (ESI$^+$-Orbitrap) m/z: $[M+H]^+$ Calcd for $C_{22}H_{28}NO_4$ 370.2018; Found 370.2020.

Compound (±)-12

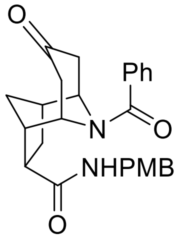

To a stirred solution of substrate **11** (160 mg, 0.43 mmol) in DCM (4 mL) at 0 °C under nitrogen was added TFA (1 mL, 13 mmol) dropwise. The reaction was warmed to rt, stirred for 3 h and evaporated. The resulting white solid was redissolved in DCM (6 mL) and cooled to 0 °C under nitrogen. Oxalyl chloride (2 mL, 23 mmol) was added dropwise and the reaction warmed to rt. After stirring for a further 40 min the reaction was evaporated. The resulting yellow solid was redissolved in DCM (4 mL) and cooled to 0 °C under nitrogen. $PMBNH_2$ (0.084 mL, 0.64 mmol), DMAP (10 mg, 0.082 mmol) and $^{i}Pr_2NEt$ (0.20 mL, 1.1 mmol) were added and the reaction allowed to warm to rt. After 14 h the reaction was quenched by the addition of 1 M HCl (20 mL) and extracted with DCM (2 × 20 mL). The combined organic phase was dried ($MgSO_4$) and evaporated to give a fluffy yellow solid. Purification by silica gel chromatography (EtOH/NH_3 in DCM, 1% to 5% as eluent) afforded the title compound (128 mg, 64%) as a clear viscous oil. ν_{max} /cm^{-1} 3313, 2951, 1704, 1616, 1511, 1423, 1362, 1245. ^{1}H NMR ($CDCl_3$, 400 MHz) *mixture of rotamers*: δ 1.44 (d, 1H (*minor*), J = 13.5 Hz), 1.79 – 1.96 (m, 2H (*major and minor*)), 2.05 (ddd, 1H (*minor*), J = 21.4, 12.3, 6.2 Hz), 2.19 (app. q, 1H (*major*), J = 4.8 Hz), 2.32 – 2.64 (m, 4H (*major and minor*)), 2.76 – 2.86 (m, 1H (*major and minor*)), 2.90 (dt, 1H (*major*), J = 11.5, 5.5 Hz), 3.75 (s, 3H ((*minor*)), 3.76 (s, 3H (*major*)), 3.90 (app. t, 1H (*major*), J = 4.8 Hz), 4.18 – 4.31 (m, 1H (*major and minor*)), 4.35 – 4.48 (m, 2H (*major and minor*)), 4.95 (app. t, 1H (*minor*), J = 5.8 Hz), 5.09 (t, 1H (*major*), J = 5.9 Hz), 5.67 – 5.75 (m, 1H (*major and minor*)), 6.78 – 6.86 (m, 2H (*major and minor*)), 7.03 – 7.11 (m, 2H (*minor*)), 7.26 – 7.31 (m, 2H (*major*)), 7.37 – 7.43 (m, 4H (*major and minor*)), 7.51 – 7.56 (m, 1H (*major and minor*)). $^{13}C\{^{1}H\}$ NMR ($CDCl_3$, 101 MHz) *mixture of rotamers* δ: 28.9 (*minor*), 29.5 (*minor*), 29.6 (*major*), 30.0 (*major*), 39.2 (*minor*), 39.4 (*major*), 43.32 (*minor*), 43.4 (*minor*), 43.5 (*major*), 43.6 (*major*), 43.8 (*major*), 44.5 (*minor*), 45.1 (*minor*), 45.4 (*major*), 46.6 (*major*), 47.1 (*major*), 47.4 (*minor*), 50.9 (*minor*), 51.9 (*minor*), 55.4 (*major*), 56.0 (*major*), 114.2 (*major*), 114.2 (*minor*), 126.5 (*major*), 127.0 (*minor*), 128.4 (*minor*), 128.8 (*major*), 129.4, 129.6, 129.9, 130.2, 130.6, 135.8, 159.1 (*major*), 159.2 (*minor*), 170.7 (*major*), 170.8 (*major and minor*), 171.4 (*minor*), 208.1 (*major and minor*). HRMS (ESI^{+}-Orbitrap) m/z: $[M+H]^{+}$ Calcd for $C_{26}H_{29}N_2O_4$ 433.2127; Found 433.2125.

Compound (±)-13

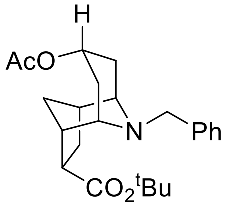

To a stirred solution of substrate **10a** (200 mg, 0.563 mmol) in MeOH (3.3 mL) at rt was added sodium borohydride (149 mg, 3.94 mmol) in water (5.6 mL) followed by further MeOH (13.2 mL). The reaction was stirred for 24 h. The mixture was added to sat. aq. $NaHCO_3$ (40 mL) and extracted with DCM (3 × 40 mL). Drying ($MgSO_4$) and evaporation gave a white solid which was redissolved in DCM (10 mL). $^iPr_2NEt$ (0.40 mL, 2.2 mmol) and DMAP (10 mg, 0.082 mmol) were added and the mixture cooled to 0 °C followed by the dropwise addition of Ac_2O (0.20 mL, 2.1 mmol). The reaction was allowed to warm to rt over 1 h, stirred for a further 1 h and poured onto sat. aq. $NaHCO_3$ (30 mL). The mixture was extracted with DCM (2 × 30 mL), dried ($MgSO_4$) and evaporated to give an orange oil. Purification by silica gel chromatography (Et_2O/petrol, 5:95 to 1:9 as eluent) afforded the title compound (195 mg, 87% over two steps) as a clear oil. Mp. 148 – 151 °C. ν_{max} /cm^{-1} (film) 2934, 1732, 1366 and 1233. ^{1}H NMR ($CDCl_3$, 400 MHz): δ 1.11 – 1.21 (m, 2H, 2 × CH*H*), 1.37 (dd, 1H, J = 15.6, 4.4 Hz, CH*H*), 1.48 (s, 9H, C*Me*$_3$), 1.71 (td, 1H, J = 12.1, 6.0 Hz, CH(OH)CH*H*), 1.85 – 1.92 (m, 1H), 2.05 – 2.12 (m, 4H, *Me*CO_2 and C*H*H), 2.19 – 2.30 (m, 2H, 2 × C*H*H), 2.35 (app. q, 1H, J = 5.1 Hz, NCHC*H*), 2.44 – 2.54 (m, 2H, C*H*H and NC*H*), 2.72 (dt, 1H, J = 11.0, 5.1 Hz, C*H*CO_2tBu), 3.27 – 3.37 (m, 2H, NC*H*CHCH($CO_2{}^t$Bu) and NC*H*HPh), 3.55 (d, 1H, J = 13.0 Hz, NCH*H*Ph), 5.36 (tt, 1H, J = 10.1, 4.3 Hz, AcOC*H*), 7.19 – 7.24 (m, 1H, CH_{Ar}), 7.31 (t, 2H, J = 7.5 Hz, 2 × CH_{Ar}), 7.42 (d, 2H, J = 7.5 Hz, 2 × CH_{Ar}). ^{13}C{^{1}H} NMR ($CDCl_3$, 101 MHz): δ 21.7 (*Me*CO_2), 27.4 (CH_2), 27.7 (CH_2), 28.4 (CMe_3), 28.5 (CH_2), 30.0 (CH_2), 40.6 (NCH*C*H), 44.6 (NCH*C*H), 46.6 ($CHCO_2{}^t$Bu), 52.3 (NCH), 54.0 (NCH), 58.1 (N*C*H_2Ph), 64.3 (AcO*C*H), 79.7 (*C*Me_3), 127.1 (*C*H_{Ar}), 128.3 (2 × *C*H_{Ar}), 129.3 (2 × *C*H_{Ar}), 138.9 (C_{qAr}), 170.8 (Me*C*O_2), 174.1 (*C*$O_2{}^t$Bu). HRMS (ESI$^+$-Orbitrap) m/z: [M+H]$^+$ Calcd for $C_{24}H_{34}NO_4$ 400.2488; Found 400.2485.

Compound (±)-15

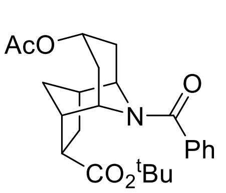

A stirred suspension of substrate **13** (175 mg, 0.422 mmol) in MeOH (8 mL) was degassed by sparging with nitrogen and the mixture heated to 60 °C. Ammonium formate (130 mg, 2.1 mmol) and Pd/C (10

wt%, 55 mg, 12 mol%) were added and the reaction stirred at reflux. After 80 min the reaction was cooled, diluted with DCM (25 mL) and filtered through Celite. Evaporation gave a white semi-solid which was dissolved in DCM (10 mL) and cooled to 0 °C under nitrogen. DMAP (10 mg, 0.082 mmol) and $^{i}Pr_2NEt$ (0.225 mL, 1.26 mmol) were added to the stirred solution followed by the dropwise addition of benzoyl chloride (0.10 mL, mmol). The reaction was allowed to warm to rt. After 15 h the reaction was quenched by the addition of sat. aq. $NaHCO_3$ (25 mL) and extracted with DCM (2 × 25 mL). The combined organic phase was dried ($MgSO_4$) and evaporated to give the crude product as a yellow oil. Purification by silica gel chromatography (EtOAc/petrol, 1:9 to 2:3 as eluent) afforded the title compound (140 mg, 78% over two steps) as a white solid. Mp. 85 - 87 °C. ν_{max} /cm^{-1} (film) 2976, 2940, 1723, 1639, 1421, 1366 and 1239. ^{1}H NMR ($CDCl_3$, 400 MHz): δ 1.31 – 1.39 (m, 1H, CHCH*H*CH), 1.41 – 1.49 (m, 10H, C*Me*$_3$ and CH(OAc)CH*H*), 1.58 – 1.69 (m, 1H, CH(OH)CH*H*), 1.78 – 1.89 (m, 1H, CH*H*CH($CO_2{}^tBu$)), 1.97 – 2.12 (m, 5H), 2.30 (dt, 1H, J = 14.0, 9.4 Hz, CH(OH)C*H*H), 2.39 (dd, 1H, J = 12.8, 2.5 Hz, CHC*H*HCH), 2.51 (app. q, 1H, J = 4.9 Hz, C*H*CH($CO_2{}^tBu$)), 2.61 (dt, 1H, J = 13.7, 9.1 Hz, CH(OH)C*H*H), 2.87 (dt, 1H, J = 11.4, 5.5 Hz, C*H*$CO_2{}^tBu$), 3.59 – 3.66 (m, 1H, NC*H*), 4.83 (app. pent, 1H, J = 8.9 Hz, C*H*(OH)), 4.91 (dd, 1H, J = 8.8, 4.2 Hz, NC*H*), 7.31 – 7.47 (m, 5H). ^{13}C{^{1}H} NMR ($CDCl_3$, 101 MHz): δ 21.4 (Me), 28.5 (C*Me*$_3$), 28.5 (*C*H$CO_2{}^tBu$), 28.7 (CH*C*H$_2$CH), 29.4 (CH(OH)*C*H$_2$), 31.5 (CH(OH)*C*H$_2$), 39.3 (NCH*C*H), 43.2 (NCH*C*H), 44.7 (NCH), 46.2 (*C*HCO_2tBu), 54.2 (NCH), 65.8 (*C*H(OH), 81.2 (*C*Me$_3$), 126.2 (CH_{Ar}), 128.8 (CH_{Ar}), 129.1 (CH_{Ar}), 136.6 (C_{qAr}), 170.3, 170.9, 172.2. HRMS (ESI$^+$-Orbitrap) m/z: $[M+H]^+$ Calcd for $C_{24}H_{32}NO_5$ 414.2281; Found 414.2278.

Compound (±)-16

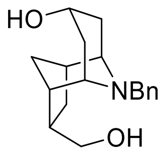

To a stirred solution of substrate **10a** (100 mg, 0.28 mmol) in anhydrous THF (3 mL) under nitrogen was added $LiAlH_4$ (250 mg, 6.6 mmol), the reaction stirred for 30 min and heated to 60 °C. After 90 min the reaction was cooled to 0 °C, diluted with Et_2O (10 mL) and quenched by the sequential addition of water, 15% aq. NaOH and further water. Magnesium sulphate (excess) was added and the reaction stirred at rt. After 10 min the reaction was filtered through Celite, washing with DCM. The filtrate was evaporated to afford the crude title compound (72 mg, 90%) as a white semi-solid which was used directly. Partial analytical data on crude material: ν_{max} /cm^{-1} (film) 3347 (br), 2927, 2876, 1452 and 1130. ^{1}H NMR ($CDCl_3$, 400 MHz): δ 1.14 (dt, 1H, J = 12.1, 4.2 Hz, CHCH*H*CH), 1.22 (dd, 1H, J = 15.4, 3.9 Hz, CH(OH)CH*H*), 1.39 (dd, 1H, J = 15.4, 3.8 Hz, CH(OH)CH*H*), 1.62 – 1.72 (m, 2H, CH_2(OH)CHC*H*$_2$), 1.94 – 2.17 (m, 3H, (HO)CH_2C*H*C*H*CH_2C*H*), 2.30 – 2.42 (m, 2H, CHC*H*HCH and

CH(OH)C*H*H), 2.49 – 2.63 (m, 2H, CH(OH)C*H*H and NCH), 3.18 (dd, 1H, J = 8.8, 4.5 Hz, NCH), 3.25 (d, 1H, J = 12.6 Hz, CH*H*Ph), 3.60 (d, 1H, J =12.6 Hz, NC*H*HPh), 3.85 – 3.94 (m, 1H, CH*H*OH), 3.97 (dd, 1H, J = 10.9, 2.6 Hz, C*H*HOH), 4.50 (ddd, 1H, J = 9.9, 4.8, 4.0 Hz, C*H*OH), 5.88 (br s, 1H, OH), 7.22 – 7.37 (m, 5H, Ph). $^{13}C\{^1H\}$ NMR ($CDCl_3$, 101 MHz): δ 28.1 ((HO)CH_2CH*C*H), 28.7 (CH*C*H_2CH), 30.0 (C*H*H), 31.9 (CH(OH)*C*H_2), 32.1 (CH(OH)*C*H_2), 40.7 (NCH*C*H), 42.3 ((HO)CH_2*C*H), 46.2 (NCH*C*H), 53.4 (NCH), 54.7 (NCH) 57.3 (NCH_2), 60.2 (*C*H(OH), 62.6 (*C*H_2OH), 127.6, 128.5, 128.7, 129.3, 129.4, 129.5, 137.0. HRMS (ESI$^+$-Orbitrap) m/z: $[M+H]^+$ Calcd for $C_{18}H_{26}NO_2$ 288.1964; Found 288.1970.

Compound (±)-17

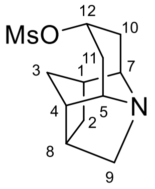

To a stirred solution of crude substrate **16** (72 mg, <0.25 mmol) in degassed MeOH (5 mL) at rt under nitrogen was added 10% Pd/C (35 mg, 13 mol%) followed by ammonium formate (79 mg, 1.3 mmol) and the reaction heated to reflux. After 150 minutes the mixture was cooled to rt, diluted with DCM (3 mL) and filtered through Celite, washing with DCM, to give a clear solution. Evaporation afforded the title crude secondary amine as a white semi-solid. To a stirred solution of 50% of this crude product (0.12 mmol) in DCM (3 mL) at 0 °C under nitrogen was added $^iPr_2NEt$ (0.068 mL, 0.39 mmol) followed by MsCl (0.022 mL, 0.028 mmol) dropwise. After 45 min the reaction was warmed to rt and stirred for a further 2 h. An aliquot (1 mL) was removed, leaving 67% of the reaction mixture (0.08 mmol). After a further 1 h the remainder was diluted with DCM (10 mL) and washed with 15% aq. NaOH/sat. aq. $NaHCO_3$ (1:1, 10 mL). The aqueous phase was extracted with DCM (5 mL) and the combined organic phase dried ($MgSO_4$) and evaporated to give a white semi-solid. Purification by silica gel chromatography (EtOH/aq. NH_3 in DCM, 0% to 20% as eluent) afforded the title compound (10 mg, 50%). ν_{max} /cm^{-1} (film) 2939, 1457, 1351 and 1172. ^1H NMR ($CDCl_3$, 400 MHz): δ 1.41 – 1.46 (m, 1H, C_3H*H*), 1.49 (ddd, 1H, J = 13.9, 10.0, 2.3 Hz, C_{10}H*H*), 1.69 – 1.86 (m, 3H, C_8*H* and C_2*H*$_2$), 2.04 (ddd, 1H, J = 14.1, 10.5, 3.0 Hz, C_{11}H*H*), 2.33 – 2.44 (m, 2H, C_1*H* and C_3H*H*), 2.51 – 2.63 (m, 3H, C_3*H*H, C_1*H* and C_4*H*) 2.77 (ddd, 1H, J = 11.3, 5.1, 2.6 Hz, C_9HH), 2.88 (d, 1H, J = 11.2 Hz, C_9*H*H), 3.00 (s, 3H, SO_2*Me*), 3.04 – 3.14 (m, 2H, C_5H and C_7H), 5.17 (tt, 1H, J = 10.1, 7.5 Hz, CHOMs). $^{13}C\{^1H\}$ NMR ($CDCl_3$, 101 MHz): δ 29.8 ($C_{11}H_2$), 31.5 (C_3H_2), 33.7 ($C_{10}H_2$), 37.1 (C_4H), 38.2 (SO_2Me), 41.3 (C_1H), 41.7 (C_2H_2), 41.8 (C_8H), 56.9 (C_7H), 62.9 (C_5H), 63.2 (C_9H_2), 75.5 (C_{12}HOMs). HRMS (ESI$^+$-Orbitrap) m/z: $[M+H]^+$ Calcd for $C_{12}H_{20}NO_3S$ 258.1164; Found 258.1170.

Compound (±)-18a

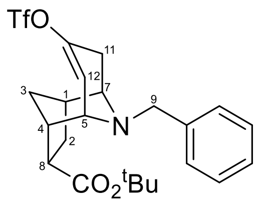

To a stirred solution of substrate **10a** (100 mg, 0.282 mmol) in dry THF (4 mL) at -78 °C under argon was added NaHMDS (0.48 mL of a 2.0 M solution in THF, 0.96 mmol) dropwise over 5 min. The reaction was stirred for 45 min and $PhNTf_2$ (400 mg, 1.12 mmol) added in one portion. The reaction was allowed to warm to rt over 19 h and was partitioned between Et_2O (25 mL) and sat. aq. $NaHCO_3$ (20 mL). The phases were separated and the organic phase dried ($MgSO_4$) and evaporated to give the crude product as an orange oil. Purification by silica gel chromatography (Et_2O/petrol, 5:95 to 1:4 as eluent) afforded the title compound (128 mg, 93%) as a clear oil. ν_{max} /cm^{-1} (neat) 2977, 2938, 2886, 1724, 1417, 1211, 1140. ^{1}H NMR ($CDCl_3$, 400 MHz): δ 1.23 (dt, 1H, J = 12.4, 4.3 Hz, $C_3\mathit{H}$H), 1.51 (s, 9H, C*Me*$_3$), 1.74 (td, 1H, J = 12.1, 6.1 Hz, $C_2\mathit{H}$H), 1.90 (dd, 1H, J = 18.2, 1.8 Hz, $C_{11}\mathit{H}$H), 1.94 – 2.01 (m, 1H, $C_1\mathit{H}$), 2.13 (dd, 1H, J = 10.2, 3.2 Hz, $C_3H\mathit{H}$), 2.20 (ddd, 1H, J = 12.5, 4.7, 2.8 Hz, $C_2H\mathit{H}$), 2.42 (app. q, 1H, J = 4.8 Hz, $C_4\mathit{H}$), 2.50 (dd, 1H, J = 18.2, 6.9 Hz, $C_{11}H\mathit{H}$), 2.67 – 2.72 (m, 1H, $C_7\mathit{H}$), 2.90 (dt, 1H, J = 11.0, 5.1 Hz, $C_8\mathit{H}$), 3.42 – 3.52 (m, 2H, $C_9\mathit{HH}$), 3.76 – 3.81 (m, 1H, $C_5\mathit{H}$), 5.81 (d, 1H, J = 5.5 Hz, $C_{12}\mathit{H}$), 7.26 (t, 1H, J = 6.4 Hz, $C_{16}\mathit{H}$), 7.30 (app. t, 2H, J = 7.4 Hz, 2 × $C\mathit{H}_{meta}$), 7.42 (d, 2H, J = 7.5 Hz, 2 × $C\mathit{H}_{ortho}$). ^{13}C{^{1}H} NMR ($CDCl_3$, 101 MHz): δ 28.0 (C_{11}), 28.4 (C*Me*$_3$), 29.4 (C_3), 31.4 (C_2), 41.4 (C_4), 41.8 (C_1), 48.0 (C_8), 54.1 (C_7), 56.9 (C_9), 57.7 (C_5), 80.1 (C_{22}), 118.6 (C_{12}), 118.7 (q, *J* 320, CF_3) 127.3 (C_{16}), 128.4 ($C_{15/17}$), 129.5 ($C_{14/18}$), 138.2 (C_{10}), 149.4 (C_{13}), 173.7 (C_{19}). HRMS (ESI$^+$-Orbitrap) m/z: [M+H]$^+$ Calcd for $C_{23}H_{29}NO_5F_3$ 488.1720; Found 488.1713.

Compound (±)-18b

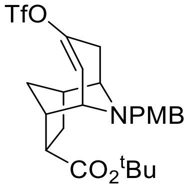

To a stirred solution of substrate **10b** (108 mg, 0.281 mmol) in dry THF (4 mL) at -78 °C under argon was added NaHMDS (0.50 mL of a 2 M solution in THF, 1 mmol) dropwise over 5 min. The reaction was stirred for 1 h and $PhNTf_2$ (400 mg, 1.12 mmol) added in one portion. The reaction was allowed to warm to rt over 19 h and was partitioned between Et_2O (25 mL) and sat. aq. $NaHCO_3$ (20 mL). The phases were separated and the organic phase dried ($MgSO_4$) and evaporated to give the crude product as an orange solid. Purification by silica gel chromatography (Et_2O/petrol, 5:95 to 15:85 as eluent)

afforded the title compound (124 mg, 86%, 95:5 ratio of regioisomers) as a clear oil. ν_{max} /cm^{-1} (neat); 2933, 2838, 1722, 1513, 1415, 1243, 1207. ^{1}H NMR ($CDCl_3$, 400 MHz): δ 1.22 (dt, 1H, J = 11.7, 3.5 Hz, CHCH*H*CH), 1.50 (s, 9H, $CO_2C(Me)_3$), 1.73 (td, 1H, J = 12.0, 6.0 Hz, ^{t}BuO$_2$CCHCH*H*CH), 1.89 (dd, 1H, J = 18.2, 1.9 Hz, CH=COTfCH*H*), 1.97 (app. q, 1H, J = 5.2 Hz, ^{t}BuO$_2$CCHCH$_2$C*H*), 2.05 – 2.21 (m, 2H, CHCH*H*CH and ^{t}BuO$_2$CCHCH*H*), 2.41 (app. q, 1H, J = 4.8 Hz, ^{t}BuO$_2$CC*H*), 2.48 (dd, 1H, J = 18.2, 6.9 Hz, CH=COTfCH*H*), 2.69 (app. t, 1H, J = 4.7 Hz, NC*H*CH$_2$COTf), 2.89 (dt, 1H, J = 10.9, 5.0 Hz, ^{t}BuO$_2$CCHC*H*CH$_2$), 3.40 (AB-q, 1H, NC*H*$_2$Ph), 3.73 – 3.82 (m, 4H, ArO*Me* and NC*H*CH=COTf), 5.78 (d, 1H, J = 5.6 Hz, C*H*=COTf), 6.86 (d, 2H, J = 8.5 Hz, 2 × CH_{Ar}), 7.33 (d, 2H, J = 8.6 Hz, 2 × CH_{Ar}). ^{13}C{^{1}H} NMR ($CDCl_3$, 101 MHz): δ 28.0 (CH=COTf*C*H$_2$), 28.5 (C(*Me*)$_3$), 29.5 (^{t}BuO$_2$CCH*C*H$_2$), 31.4 (CH*C*H$_2$CH), 41.4 (^{t}BuO$_2$C*C*H), 48.0 (^{t}BuO$_2$CCH*C*HCH$_2$), 53.9 (N*C*HCH$_2$COTf), 55.3 (ArO*Me*), 56.2 (N*C*H$_2$Ph), 57.6 (N*C*HCH=COTf), 80.1 (CO$_2$*C*(Me)$_3$), 113.8 (CH_{Ar}), 118.6 (*C*H=COTfCH$_2$), 120.3 (q, *J* = 320, SO$_2$*C*F$_3$), 130.2 (C_{qAr}), 130.5 (CH_{Ar}), 149.4 (CH=*C*OTf), 159.9 (C_{qAr}), 173.7 (*C*O$_2$^{t}Bu). HRMS (ESI$^+$-Orbitrap) [M+H]$^+$ Calcd for $C_{24}H_{30}NO_6F_3$ 518.1825; Found 518.1821.

Compound (±)-20

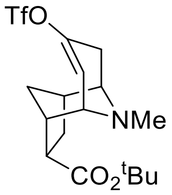

To a stirred solution of substrate **10i** (102 mg, 0.37 mmol) in dry THF (5.2 mL) at -78 °C under argon, NaHMDS (650 µL of 2M NaHMDS in THF, 1.3 mmol) was added dropwise over 5 min and stirred for 1 h. PhNTf$_2$ (522 mg, 1.5 mmol) was added in one portion. The mixture was stirred further for 4 h, quenched with saturated $NaHCO_3$ (25 mL) and extracted with Et_2O (2 × 30 mL). The combined organic phase was dried ($MgSO_4$) and evaporated to give an orange oily solid (34:66 alkene regioselectivity within the crude ^{1}H NMR spectrum). Purification by silica gel chromatography (Et_2O/petrol containing 1% Et_3N, 1:99 to 15:85 as eluent) afforded the product as a clear oil (96 mg, 63%). (2:1 mixture of regioisomers); ν_{max} /cm^{-1} (neat) 2976, 2936, 2882, 1718, 1675, 1415, 1207. Major isomer: ^{1}H NMR ($CDCl_3$, 400 MHz): δ 1.14 – 1.22 (m, 1H, CHCH*H*CH), 1.46 (s, 9H, $CO_2C(Me)_3$), 1.77 (td, 1H, J = 12.0, 6.0 Hz, O$_2$CCHCH*H*), 1.93 – 2.09 (m, 3H, O$_2$CCHCH$_2$C*H* and CHCH*H*CH and CH*H*COTf), 2.12 – 2.18 (m, 4H, N*Me* and O$_2$CCHCH*H*), 2.29 – 2.34 (m, 1H, O$_2$CC*H*), 3.57 (app. t, 1H, J = 4.8 Hz, NC*H*CH=), 5.66 – 5.73 (m, 1H, C*H*=COTf). ^{13}C{^{1}H} NMR ($CDCl_3$, 101 MHz): δ 28.1 (CH*C*H$_2$CH), 28.4 (C(*Me*$_3$), 28.6 (CH*C*H$_2$CH), 32.3 (O$_2$CH*C*H$_2$), 40.4 (N*Me*), 40.5 (O$_2$C*C*H), 42.3 (O$_2$CCHCH$_2$*C*H), 47.7 (O$_2$CCH*C*H), 57.0 (N*C*HCH=), 60.0 (CH*C*HN), 79.8 (*C*(Me)$_3$), 118.3 (*C*H=COTf), (118.6, q, *J*

320, CF_3), 148.7 (CH=COTf), 173.8 ($CO_2{}^tBu$). HRMS (ESI^+-Orbitrap) $[M+H]^+$ Calcd for $C_{17}H_{25}O_5F_3S$ 412.1406; Found 412.1399.

Compound (±)-19a

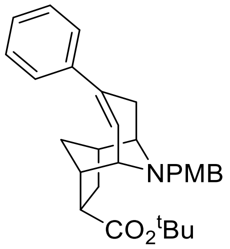

To a dry Schlenk tube were added Na_2CO_3 (70 mg, 0.66 mmol) and LiCl (18.9 mg, 0.50 mmol), followed by water (380 µL), and the mixture stirred for 5 min. A solution of enol triflate **18b** (66.3 mg, 0.13 mmol) in degassed dioxane (1.7 mL) was added followed by phenyl boronic acid (26.8 mg, 0.22 mmol) and $Pd(PPh_3)_4$ (15.1 mg, 0.013 mmol). The reaction was heated to 85 °C, stirred for 16 h and cooled to rt. The reaction was diluted with water (15 mL) and extracted with DCM (2 ×15 mL). The combined organic phase was dried ($MgSO_4$) and evaporated to give an oil. Purification by silica gel chromatography (Et_2O/petrol, 1:99 to 1:9 as eluent) afforded the product as a clear oil (31 mg, 51%). ν_{max} /cm^{-1} (neat) 2974, 2932, 2873, 2832, 1720, 1611, 1493, 1394. ^1H NMR ($CDCl_3$, 400 MHz): δ 1.14 (dt, 1H, J = 11.8, 4.1 Hz, CHCH*H*CH), 1.52 (s, 9H, C(*Me*)$_3$, 1.74 (dt, 1H, 1H, J = 11.8, 6.0 Hz, $^tBuO_2CCHCHH$), 1.94 – 2.01 (m, 2H, $^tBuO_2CCHCHCH_2CH$ and CH=CPhCH*H*), 2.11 (d, 1H, J = 11.9 Hz, CHCH*H*CH), 2.24 (dt, 1H, J = 12.1, 3.5 Hz, $^tBuO_2CCHCHH$), 2.42 (app. q, 1H, J = 4.8 Hz, $^tBuO_2CCH$), 2.55 (dd, 1H, J = 18.2, 7.0 Hz, CH=CPhCH*H*), 2.67 (t, 1H, J = 5.9 Hz, CH_2CHCHN), 2.87 (dt, 1H, J = 10.9, 5.0 Hz, $^tBuO_2CCHCHCHCHN$), 3.41 (s, 2H, NCH_2Ph), 3.68 (t, 1H, J = 5.2 Hz, $^tBuO_2CCHCHCHCHN$), 3.79 (s, 3H, ArO*Me*), 6.22 (d, 1H, J = 5.3 Hz, C*H*=CPh), 6.85 (d, 2H, J = 8.5 Hz, 2 × CH_{Ar}), 7.26 – 7.39 (m, 5H, 5 × CH_{Ar}), 7.49 (d, 2H, J = 7.8 Hz, 2 × CH_{Ar}). $^{13}C\{^1H\}$ NMR ($CDCl_3$, 101 MHz): δ 26.4 (CH=PhCH_2) 28.5 ($CO_2C(Me)_3$), 30.1 (^tBu--O_2CCHCH_2), 31.4 ($CHCH_2CH$) 42.0 ($^tBuO_2CCH$), 42.2 ($^tBuO_2CCHCHCH_2CH$), 47.7 ($^tBuO_2CCHCH$), 53.3 (CH_2CHCHN), 55.3 (ArO*Me*), 56.7 (NCH_2Ph), 58.0 ($^tBuO_2CCHCHCHN$), 79.7 ($CO_2C(Me)_3$), 113.5 (CH_{Ar}), 124.6 (*C*H=CPhCH$_2$), 124.9 (CH_{Ar}), 127.3 (CH_{Ar}), 128.5 (CH_{Ar}) 130.5 (CH_{Ar}), 131.6 (C_{qAr}), 137.7 (C_{qAr}), 140.4 (CH=*C*PhCH$_2$), 158.6 (C_{qAr}), 174.2 ($CO_2{}^tBu$). HRMS (ESI^+-Orbitrap) m/z: $[M+H]^+$ Calcd for $C_{29}H_{35}NO_3$ 446.2697; Found 446.2688.

Compound (±)-19b

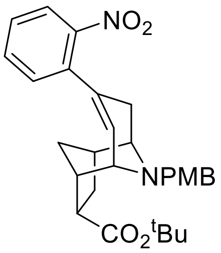

To a Schlenk tube under argon were added LiCl (22 mg, 0.52 mmol), Na_2CO_3 (81 mg, 0.76 mmol) and water (0.39 mL), the resulting mixture was stirred for 5 min and a solution of enol triflate **18b** (80 mg, 0.15 mmol) in 1,4-dioxane (2 mL) added. The mixture was degassed by sparging with argon and 2-nitrophenyl boronic acid (43 mg, 0.26 mmol) and $Pd(PPh_3)_4$ (17.6 mg, 0.015 mmol) added. The stirred mixture was heated to 85 °C for 14 h, cooled to rt and partitioned between water (15 mL) and DCM (15 mL). The phases were separated and the organic phase was dried ($MgSO_4$) and evaporated to give the crude product as a black oil. Purification by silica gel chromatography (Et_2O/petrol, 1:9 to 3:7 as eluent) afforded the title compound (39 mg, 51%) as a yellow semi-solid. ν_{max} /cm^{-1} (film) 2933, 1719, 1610, 1525, 1512, 1465, 1363 and 1247. *Major*: ^{1}H NMR ($CDCl_3$, 400 MHz): δ 1.16 – 1.27 (m, 1H, CHCH*H*CH), 1.51 (s, 9H, C*Me*$_3$), 1.70 – 1.82 (m, 2H, NCHCH*H* and CH*H*CHCO$_2{}^t$Bu), 1.99 (d, 1H, J = 5.0 Hz, C*H*CHCHCO$_2{}^t$Bu), 2.18 – 2.34 (m, 2H, CHC*H*HCH and C*H*HCHCO$_2{}^t$Bu), 2.38 – 2.50 (m, 2H, NCHC*H*H and C*H*CHCO$_2{}^t$Bu), 2.60 (app. t, 1H, J = 5.8 Hz, NC*H*CH$_2$), 2.87 (dt, 1H, J = 10.8, 5.0 Hz, C*H*CO$_2$tBu), 3.44 (d, 1H, J = 12.9 Hz, NCH*H*Ar), 3.57 – 3.71 (m, 2H, NC*H*CH= and NC*H*HAr), 3.79 (s, 3H, OMe), 5.81 (d, 1H, J = 5.0, =C*H*), 6.86 (d, 2H, J = 8.2 Hz, 2 × CH$_{Ar\ (PMB)}$), 7.35 – 7.46 (m 4H, 4 × CH$_{Ar}$), 7.56 (t, 1H, J = 7.6 Hz, CH$_{Ar}$), 7.80 (d, 1H, J = 8.2 Hz, CH$_{Ar}$). ^{13}C{^{1}H} NMR ($CDCl_3$, 101 MHz): δ 28.3 (NCH*C*H$_2$), 28.5 (C*Me*$_3$), 30.0 (CH*C*H$_2$CH), 31.5 (*C*H2CHCO$_2{}^t$Bu), 41.8 (*C*HCHCO$_2{}^t$Bu), 42.4 (*C*HCHCHCO$_2{}^t$Bu), 47.8 (*C*HCO$_2{}^t$Bu), 53.0 (N*C*HCH$_2$), 55.3 (OMe), 56.2 (N*C*H$_2$Ar), 57.4 (N*C*H-CH=), 79.7 (*C*Me$_3$), 113.6 (2 × CH$_{Ar(PMB)}$), 124.1 (CH$_{Ar}$), 128.0 (CH$_{Ar}$), 128.9 (CH$_{Ar}$), 130.6 (2 × CH$_{Ar(PMB)}$), 131.1 (C$_{qAr}$), 131.5 (C$_{qAr}$), 132.7 (C$_{qAr}$), 137.4 (CHAr), 137.7 (CH=*C*$_q$-Ar), 148.7 (C$_{qAr}$), 158.6 (*C*$_q$OMe), 174.2 (ester). HRMS (ESI$^+$-Orbitrap) m/z: [M+H]$^+$ Calcd for $C_{29}H_{35}N_2O_5$ 491.2546; Found 491.2541.

Compound (±)-19c

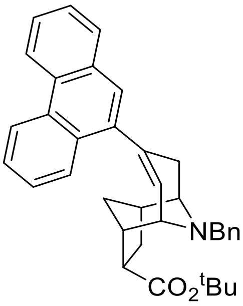

To a dry Schlenk tube were added Na_2CO_3 (145 mg, 1.4 mmol) and LiCl (44 mg, 0.90 mmol), followed by water (380 µL), and the mixture stirred for 5 min. A solution of enol triflate **18a** (133 mg, 0.27 mmol) in dioxane (3.5 mL, degassed via argon sparge for 1.5 min prior to use) was added followed by 9-phenanthacenyl boronic acid (107 mg, 0.5 mmol) and $Pd(PPh_3)_4$ (26 mg, 0.032 mmol). The reaction was heated to 85 °C, stirred for 15 h and cooled to rt. The reaction was diluted with water (30 mL) and extracted with DCM (2 × 30 mL). The combined organic phase was dried ($MgSO_4$) and evaporated to give a crude oil. Purification by silica gel chromatography (Et_2O/petrol, 1:99 to 1:9 then $CHCl_3$/petrol, 1:1 to 1:0 as eluent) afforded the product as a clear oil (62 mg, 42%). ν_{max} /cm^{-1} (neat) 3061, 2975, 2930, 2871, 2826, 2252, 1718, 1159, 1138. ^{1}H NMR ($CDCl_3$, 400 MHz): δ 1.29 (dt, 1H, J = 11.8, 4.3 Hz, CH*CH**H*CH), 1.49 (s, 9H, $CO_2C(Me)_3$, 1.75 (td, 1H, J = 12.0 Hz, 5.9 Hz, ^{t}BuO$_2$CCHCH*H*), 1.96 (d, 1H, 17.5 Hz, CH=CPhCH*H*), 2.03 (app. q, 1H, J = 5.0 Hz, CHCH$_2$C*H*), 2.25 (dt, 1H, J = 13.1, 3.1 Hz, ^{t}BuO$_2$CCHC*H*), 2.45 – 2.64 (m, 4H, ^{t}BuO$_2$CC*H* and CH=CPhC*H*$_2$), 2.89 (dt, 1H, J = 10.9, 5.1 Hz, CHCH*H*CH), 3.66 (d, 1H, 13.1 Hz, NCH*H*Ph), 3.72 (app. t, 1H, J = 5.0 Hz, ^{t}BuO$_2$CCHCHCHC*H*N), 3.85 (d, 1H, J = 13.1 Hz NCH*H*Ph), 5.89 (d, 1H, J = 5.0 Hz, C*H*=CPh), 7.16 – 7.22 (m, 2H, 2 × CH_{Ar}), 7.29 (t, 2H, J = 7.5 Hz, 2 × CH_{Ar}), 7.46 – 7.65 (m, 7H, 7 × CH_{Ar}), 7.83 (d, 1H, J = 7.7 Hz, CH_{Ar}), 8.23 – 8.28 (m, 1H, CH_{Ar}), 8.62 (d, 1H, J = 7.9 Hz, CH_{Ar}), 8.67 – 8.72 (m, 1H, CH_{Ar}). ^{13}C{^{1}H} NMR ($CDCl_3$, 101 MHz): δ 28.5 ($CO_2C(Me)_3$ 30.3 (CH=CPh*C*H$_2$), 30.4 (CH*C*H$_2$CH), 31.4 (^{t}BuO$_2$CCH*C*H$_2$), 42.0 (^{t}BuO$_2$C*C*H), 42.4 (^{t}BuO$_2$CCHCHCH$_2$*C*H), 47.7 (^{t}BuO$_2$CCH*C*H), 55.3 (CH$_2$CH*C*HN), 57.8 (^{t}BuO$_2$CCHCH*C*HN), 57.9 (N*C*H$_2$Ph), 79.7 (CO$_2$*C*(Me)$_3$), 122.6 (*C*H_{Ar}), 123.6 (*C*H_{Ar}), 125.8 (*C*H_{Ar}), 126.5 and 126.6 (2 × *C*H_{Ar}), 126.8 (*C*H_{Ar}) , 127.0 (*C*H_{Ar}), 128.1 (*C*H=CPhCH$_2$), 128.3 and 128.5 (2 × *C*H_{Ar}), 129.5 (*C*H_{Ar}), 129.8 (C_{qAr}), 130.7 and 130.8 (2 × C_{qAr}), 131.8 (C_{qAr}), 139.3 and 139.4 (2 × C_{qAr}), 140.1 (CH=*C*Ph), 174.2 (*C*O$_2$^{t}Bu). HRMS (ESI$^+$-Orbitrap) m/z: $[M+H]^+$ Calcd for $C_{36}H_{37}NO_2$ 516.2904; Found 516.2898.

Compound (±)-19d

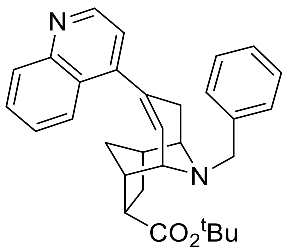

To a Schlenk tube under argon was added Na_2CO_3 (69 mg, 0.66 mmol), LiCl (19 mg, 0.45 mmol) and H_2O (0.33 mL), the mixture stirred for 5 min and a solution of enol triflate **18a** (68 mg, 0.13 mmol) in dioxane (1.7 mL) added. The stirred mixture was degassed by sparging with argon, quinoline-4-boronic acid (38 mg, 0.22 mmol) and $Pd(PPh_3)_4$ (15 mg, 0.013 mmol) added and the stirred mixture heated to 85 °C under argon. After 13 h the reaction was cooled to rt and partitioned between EtOAc (20 mL) and aq. $NaHCO_3$ (15 mL). The phases were separated, the aqueous phase extracted with EtOAc (10 mL) and the combined organic phase dried ($MgSO_4$) and evaporated to give the crude product as a yellow oil. Purification by silica gel chromatography (EtOAc/petrol, 1:9 to 2:3 as eluent) afforded the title compound (35 mg, 58%) as a clear oil. ν_{max} /cm^{-1} (film) 2975, 2934, 2873, 1721, 1578, 1505, 1458, 1365 and 1160. ^{1}H NMR ($CDCl_3$, 400 MHz): δ 1.30 – 1.38 (m, 1H, C$_3$H*H*), 1.54 (s, 9H, C*Me*$_3$), 1.81 (td, 1H, J = 11.9, 6.0 Hz, C$_2$H*H*), 1.98 (dd, 1H, J = 18.6, 2.2 Hz, C$_{11}$H*H*), 2.12 – 2.05 (m, 1H, C$_1$H), 2.30 (ddd, 1H, J = 12.6, 5.0, 2.7 Hz, C$_2$*H*H), 2.41 (d, 1H, J = 11.3 Hz, C$_3$*H*H), 2.52 (dd, 1H, J = 4,7, 4.3 Hz, 1H, C$_4$H), 2.58 (dd, 1H, J = 18.9, 6.9 Hz, C$_{11}$*H*H), 2.69 (t, 1H, J = 5.8 Hz, C$_7$*H*), 2.94 (dt, 1H, J = 11.0, 5.2 Hz, C$_8$H), 3.68 (d, 1H, J = 13.0 Hz, C$_9$H*H*), 3.76 (d, 1H, J = 13.3 Hz, C$_9$*H*H), 3.77 – 3.82 (m, 1H, C$_5$*H*), 5.97 (d, 1H, J = 5.2 Hz, C$_{12}$*H*), 7.22 – 7.28 (m, 1H, CH$_{16}$) 7.29 – 7.37 (m, 3H, CH$_{15/17}$ and CH$_{20}$), 7.51 (d, 2H, J = 7.5 Hz, C*H*$_{14/18}$), 7.59 (t, 1H, J = 7.7 Hz, C$_{27}$*H*), 7.73 (t, 1H, J = 7.8 Hz, C$_{26}$*H*), 8.15 (d, 1H, J = 8.4 Hz, C$_{25}$*H*), 8.24 (d, 1H, J = 8.1 Hz, C$_{28}$*H*), 8.88 (d, 1H, J = 4.4 Hz, C$_{21}$*H*). ^{13}C{^{1}H} NMR ($CDCl_3$, 101 MHz): δ 28.5 (C*Me*$_3$), 29.3 (C$_{11}$), 30.2 (C$_3$), 31.4 (C$_2$), 41.7 (C$_4$), 42.4 (C$_1$), 47.7 (C$_8$), 53.2 (C$_7$), 57.8 (C$_9$), 57.8 (C$_5$), 79.9 (C$_{32}$), 119.7 (C$_{20}$), 125.6 (C$_{28}$), 126.5 (C$_{24}$), 126.6 (C$_{27}$), 127.2 (C$_{16}$), 128.4 (C$_{15/17}$), 129.4 (C$_{26}$), 129.5 (C$_{14/18}$), 130.1 (C$_{25}$), 130.2 (C$_{12}$), 136.6 (C$_{19}$), 139.0 (C$_{10}$), 148.9 (C$_{23}$), 149.6 (C$_{13}$), 150.1 (C$_{21}$), 174.1 (C$_{29}$). HRMS (ESI$^+$-Orbitrap) m/z: [M+H]$^+$ Calcd for $C_{31}H_{35}N_2O_2$ 467.2699; Found 467.2704.

Compound (±)-22

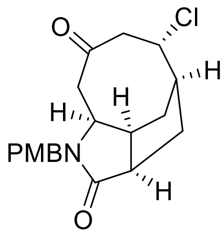

To a stirred solution of substrate **10b** (250 mg, 0.65 mmol) in DCM (8 mL) at 0 °C was added TFA (2 mL, 26 mmol) dropwise. The reaction was allowed to warm to rt. After 6 days the reaction was evaporated and the residue dissolved in DCM (10 mL) at 0 C under argon. Oxalyl chloride (2 mL, 23 mmol) was added dropwise and the reaction allowed to warm to rt. After 2 h the mixture was evaporated and purified by silica gel chromatography (Et_2O/DCM, 10% to 40% as eluent) to afford the title compound (147 mg, 65%) as a light yellow solid. ν_{max} /cm^{-1} (neat) 2939, 2836, 1676, 1612, 1512, 1450, 1245. ^{1}H NMR ($CDCl_3$, 400 MHz): δ 1.92 – 2.01 (m, 2H, CH*H*CHC=O and CHCH*H*CH), 2.21 (d, 1H, J = 15.9 Hz, CHC*H*HCH), 2.46 (ddd, 1H, J = 15.4, 12.3, 9.8 Hz, C*H*HCHC=O), 2.67 – 2.89 (m, 5H, CH_2C(O)CH_2 and C*H*CHCl), 3.13 (app. q, 1H, J = 10.4 Hz, C*H*CH_2CHCHCl), 3.25 (td, 1H, J = 12.0, 3.0 Hz, C*H*C(O)), 3.71 (d, 1H, J = 14.8 Hz, NCH*H*), 3.77 (s, 3H, OMe), 3.84 (ddd, 1H, J = 10.7, 5.5, 2.4 Hz, NC*H*), 4.19 (ddd, 1H, J = 7.1, 4.6, 2.3 Hz, C*H*Cl), 4.98 (d, 1H, J = 14.7 Hz, NC*H*H), 6.83 (d, 2H, J = 8.6 Hz, 2 × CH_{Ar}), 7.15 (d, 2H, J = 8.7 Hz, 2 × CH_{Ar}). ^{13}C{^{1}H} NMR ($CDCl_3$, 101 MHz): δ 30.4 (CH*C*H_2CH), 34.8 (*C*H_2CHC(O)), 38.3 (NCHCH), 41.8 (NCH*C*H_2), 44.3 (N*C*H_2), 46.3 (*C*H_2CHCl), 47.6 (CHClC*H*), 47.9 (*C*HCl), 55.0 (N*C*H), 55.4 (OMe), 114.3 (2 × CH_{Ar}), 127.2 (C_{qAr}), 129.8 (2 × CH_{Ar}), 159.4 (*C*OMe), 176.6 (amide), 205.7 (ketone). HRMS (ESI$^+$-Orbitrap) m/z: [M+H]$^+$ Calcd for $C_{19}H_{22}NO_3Cl$ 348.1367; Found 348.1362.

Compound (±)-23

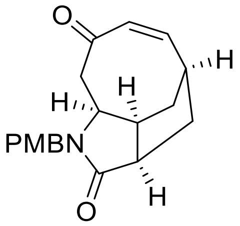

To a stirred solution of compound **10b** (500 mg, 1.3 mmol) in DCM (16 mL) at 0 °C, TFA (4.0 mL, 53 mmol) was added dropwise upon which solution turned red. The mixture was allowed to warm to rt, stirred further for 6 days and evaporated. The reaction was evaporated to give a red oil which was used without further purification. The red oil was redissolved in DCM (4 mL) under argon and cooled to 0 °C. Oxalyl chloride (4.0 mL, 47 mmol) was added dropwise. The mixture was stirred at 0 °C for 1 h, warmed to rt and stirred further for 1 h. The mixture was evaporated to give an orange solid (ratio of

chloride:alkene, 4:1) which was used without further purification. The solid was redissolved in MeCN (8 mL) at rt with stirring and DBU (240 µL, 1.6 mmol) added dropwise. The mixture was heated to 75 °C, stirred for 2.5 h and evaporated to give a crude brown oil. This was passed through a silica plug (Et_2O/DCM, 1:9 to 3:7 as eluent) to afford the product as a white solid (363 mg, 90% over 3 steps). ^{1}H NMR ($CDCl_3$, 400 MHz): δ 1.82 – 1.92 (m, 1H, CHCH*H*CH), 1.99 (ddd, 1H, J = 13.2, 9.0, 7.3 Hz, CH*H*CHCO), 2.11 (dd, 1H, J = 15.1, 2.2 Hz, CHC*H*HCH), 2.30 (dd, 1H, J = 13.2, 2.2 Hz, C*H*HCHCO), 2.68 – 2.93 (m, 3H, CH_2C=O and =CH-C*H*), 2.99 – 3.13 (m, 2H, NCHC*H* and C*H*-C=O), 3.56 (d, 1H, J = 14.9 Hz, NCH*H*), 3.78 (s, 3H, OMe), 3.90 (ddd, 1H, J = 9.6, 4.4, 2.7 Hz, NC*H*), 4.93 (d, 1H, J = 15.0 Hz, NC*H*H), 5.57 (d, 1H, J = 13.2 Hz, =C*H*-C=O), 5.86 (dd, 1H, J = 13.0, 1.4 Hz, =C*H*-CH), 6.83 (d, 2H, J = 8.6 Hz, 2 × CH_{Ar}), 7.13 (d, 1H, J = 8.6 Hz, 2 × CH_{Ar}). ^{13}C{^{1}H} NMR ($CDCl_3$, 101 MHz): δ 31.9 (CH*C*H_2CH), 35.6 (*C*H2CHC=O), 36.9 (NCH*C*H), 41.2 (=CH-*C*H), 42.0 (*C*H_2-C=O), 44.3 (N*C*H_2), 46.9 (*C*H-C=O), 55.3 (N*C*H), 55.4 (OMe), 114.2 (2 × CH_{Ar}), 127.2 (C_{qAr}), 128.4 (=*C*H-C=O), 129.8 (2 × CH_{Ar}), 141.1 (=*C*H-CH), 159.3 (*C*OMe), 177.1 (amide), 204.3 (ketone). HRMS (ESI$^+$) m/z: $[M+H]^+$ Calcd for $C_{19}H_{21}NO_3$ 312.1594; found 312.1593.

Compound (±)-24

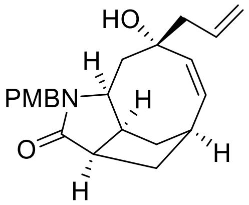

To a solution of compound **23** (95 mg, 0.30 mmol) in dry THF under argon at -78 °C was added allylmagnesium bromide (550 µL of a 1 M solution in ether, 0.55 mmol) dropwise. The stirred mixture was allowed to warm to 0 °C over 4 h. The reaction was quenched with saturated NH_4Cl (15 mL) and extracted with EtOAc (3 × 20 mL). The combined organic phase was dried ($MgSO_4$) and evaporated to give an oily solid. Purification by silica gel chromatography (EtOAc/petrol containing 1% Et_3N, 1:1 to 8:2 as eluent) afforded the product as a clear oil (94.5 mg, 89%, diastereomeric ratio 87:13). ν_{max} /cm^{-1} (neat) 3403, 2997, 2938, 1655, 1512, 1462, 1244. ^{1}H NMR ($CDCl_3$, 400 MHz): δ 1.64 – 1.76 (m, 1H, -CHCH*H*CH), 1.87 (ddd, 1H, J = 14.5 Hz, COCHCH*H*), 2.08 – 2.31 (m, 4H, COCHCH*H* and NCHC*H*$_2$ and CHCH*H*CH), 2.40 (dd, 1H, J = 13.4, 8.1 Hz, CH*H*CH=CH_2), 2.53 (dd, 1H, J = 13.4, 6.6 Hz, CH*H*CH=CH_2), 2.70 (app. q, 1H, J = 6.0 Hz, COCHCH_2C*H*), 2.83 – 2.98 (m, 2H, COC*H*C*H*), 3.76 (s, 3H, O*Me*), 3.83 (dd, 1H, J = 10.0, 6.2 Hz, NC*H*CH_2) 4.13 (d, 1H, J = 14.8 Hz, NCH*H*Ph), 5.01 (d, 1H, J = 14.7 Hz, NCH*H*Ph), 5.14 – 5.32 (m, 4H, C*H*=C*H*COH and CH=C*H*$_2$), 5.87 (ddt, 1H, J = 17.1, 10.3, 7.0 Hz, C*H*=CH_2), 6.82 (d, 2H, J = 8.3 Hz, 2 × C*H*$_{Ar}$), 7.17 (d, 2H, J = 8.3, 2 × C*H*$_{Ar}$). ^{13}C{^{1}H} NMR ($CDCl_3$, 101 MHz): δ 32.1 (CH*C*H_2CH), 36.7 (COCH*C*H_2CH), 37.4 (COCH*C*H), 39.9 (NCH*C*H_2COH), 40.1 (CH_2*C*HCH_2), 43.9 N*C*H_2Ph), 46.6 (NCO*C*H), 51.4 (*C*H_2CH=CH_2), 55.3

(ArO*Me*), 56.6 (N*C*HCOH), 72.3 ((allyl-)C_qOH), 114.0 (CH_{Ar}), 120.5 (CH=CH_2), 128.9 (C_{qAr}), 129.8 (CH_{Ar}), 131.1 (*C*H=CHC$_q$OH), 133.4 (*C*H=CH_2), 134.8 (CH=*C*HC$_q$OH), 158.9 (C_{qAr}), 177.5 (N*C*O). HRMS (ESI^+-Orbitrap) m/z: $[M+H]^+$ Calcd for $C_{22}H_{27}NO_3$ 354.2070; Found 354.2065.

Compound (±)-25

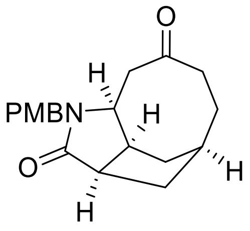

To a stirred solution of solution of compound **23** (45 mg, 0.14 mmol) in degassed MeOH (2.1 mL) at rt under argon was added ammonium formate (39 mg, 0.60 mmol) followed by 10% Pd/C (19 mg, 0.018 mmol, 13 mol%). The mixture was heated to reflux, stirred for 1.5 h and cooled to rt. The mixture was diluted with DCM (3 mL), filtered through Celite and the solids washed with DCM to give a clear filtrate. Evaporation afforded the product as clear oil (45 mg, 100%). ν_{max} /cm^{-1} (neat) 2934, 2873, 2838, 1671, 1612, 1512, 1449, 1245. ^{1}H NMR ($CDCl_3$, 400 MHz): δ 1.59 – 1.69 (m, 2H, 2 × CH*H*), 1.84 – 2.05 (m, 3H, CH_2 and CH*H*), 2.14 (ddd, 1H, J = 12.1, 7.2, 2.5 Hz, CH_2COCH*H*CH_2), 2.34 – 2.45 (m, 2H, CHCH*H*CH and OCNCHCH$_2$COCH*H*), 2.48 – 2.55 (m, 2H, CH_2C*H*$CH_2$$CH_2$CO), 2.75 (dd, 1H, J = 18.7, 4.3 Hz, OCNCHCH*H*CO), 3.07 (app. q, 1H, J = 10.4 Hz, NCOC*H*CHCH$_2$), 3.21 (td, 1H, 11.6, 2.8 Hz, NCOCHC*H*CH$_2$), 3.74 – 3.89 (m, 5H, O*Me* and NCH*H*Ph and OCNC*H*CH$_2$CO 5.08 (d, 1H, 14.8 Hz, NCH*H*Ph), 6.83 (d, 2H, J = 7.9 Hz, 2 × CH_{Ar}), 7.17 (d, 2H, J = 8.0 Hz, 2 × CH_{Ar}). ^{13}C{^{1}H} NMR ($CDCl_3$, 101 MHz): δ 33.9 (COCH$_2$*C*H$_2$), 34.1 (CH*C*H$_2$CH), 36.5 (CH*C*H$_2$CH), 37.9 (OCNCHCH$_2$*C*HCH$_2$), 38.3 (NCHCH$_2$CO*C*H$_2$CH$_2$), 38.8 (NCO*C*HCH$_2$, 41.5 (OCNCH*C*H$_2$COCH$_2$CH$_2$), 44.0 (N*C*H$_2$Ph), 48.3, NCOCH*C*HCH$_2$), 55.3 (ArO*Me*), 55.7 (OCN*C*HCH$_2$CO), 114.2 (CH_{Ar}), 127.4 (C_{qAr}), 159.2 (C_{qAr}), 177.2 (N*C*O), 214.0 (CH$_2$*C*O). HRMS (ESI^+-Orbitrap) m/z: $[M+H]^+$ Calcd for $C_{19}H_{23}NO_3$ 314.1757; Found 314.1362.

Compound (±)-26

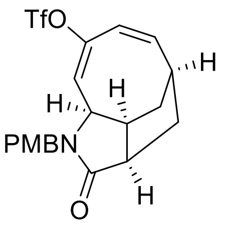

To a stirred solution of compound **23** (72 mg, 0.23 mmol) in dry THF (3.3 mL) at -78 °C under agon, NaHMDS (140 µL of 2 M NaHMDS in THF, 0.28 mmol) was added dropwise. The mixture was stirred for 1 h, $PhNTf_2$ (293 mg, 0.80 mmol) was added, and the mixture stirred further for 1 h. The reaction was partitioned between Et_2O (15 mL) and saturated $NaHCO_3$ (15 mL). The phases were separated, and the aq. phase extracted with Et_2O (15 mL). The combined organic phase was dried ($MgSO_4$) and evaporated to give a crude orange solid. Purification by silica gel chromatography (petrol/DCM 1:9 then Et_2O/DCM, 0:100 to 3:7 as eluent) afforded the product as a white solid (76 mg, 74%). ν_{max} /cm^{-1} (neat) 2939, 2915, 2839, 1677, 1512, 1413, 1242, 1204. ^{1}H NMR ($CDCl_3$, 400 MHz): δ 1.70 (ddd, 1H, J = 14.7, 8.8, 6.2 Hz, NCOCHCHCHCHC*H*), 2.04 (ddd, 1H, J = 13.3, 9.7, 7.6 Hz, NCOCHCH*H*CH), 2.39 (dd, 1H, J = 13.2, 2.3 Hz, NCOCHCH*H*), 2.75 – 2.85 (m, 2H, CHCH*H*CH and CH_2C*H*CH=CH_2), 2.91 (app. q, 1H, J = 9.4 Hz, NCOCHC*H*), 3.07 (app. t, 1H, J = 9.6 Hz, $NCOCHCH_2$), 3.42 (d, 1H, J = 14.5 Hz, NCH*H*Ph), 3.78 (s, 3H, O*Me*), 4.15 (dd, 1H, J = 10.3, 8.3 Hz, OCNC*H*CH=COTfCH), 4.96 (d, 1H, J = 14.4 Hz, NCH*H*Ph), 5.50 (dd, 1H, 12.4, 2.1 Hz, CH_2CHC*H*=CHOTf), 5.61 (d, 1H, J = 8.2 Hz, NCHC*H*=COTf), 5.83 (dd, 1H, J = 12.7, 8.3 Hz, CH_2CHC*H*=CHOTf), 6.84 (d, 2H, J = 8.4 Hz, 2 × CH_{Ar}), 7.13 (d, 2H, J = 8.4 Hz, 2 × CH_{Ar}). ^{13}C{^{1}H} NMR ($CDCl_3$, 101 MHz): δ 31.7 (NCOCH*C*H_2CH), 35.6 (NCOCH*C*H), 41.1 ($NCOCHCH_2$), 42.7 (NCOCHCH_2*C*H_2), 44.4 (N*C*H_2), 48.3 (NCO*C*HCHCH=) 55.4 (ArO*Me*), 55.9 (OCN*C*HCH=), 114.2 (*C*H_{Ar}), 118.0 (CH_2CH=*C*HOTf), 118.4 (q, *J* = 320, SO_2*C*F_3), 121.1 (NCH*C*H=CHOTf), 127.3 (*C*$_{qAr}$), 130.1 (*C*H_{Ar}), 142.8 (CH_2CHCH=*C*HOTf), 149.6 (CH=*C*OTfCH=), 159.3 (*C*$_{qAr}$), 176.7 (N*C*O). HRMS (ESI$^+$-Orbitrap) m/z: $[M+H]^+$ Calcd for $C_{20}H_{21}NO_5F_3S$ 444.1093; Found 444.1086.

Compound (±)-27

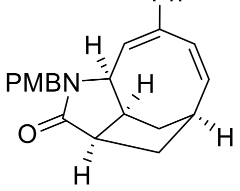

To a dry Schlenk tube were added Na_2CO_3 (91 mg, 0.86 mmol) and LiCl (24.7 mg, 0.58 mmol), followed by water (0.5 mL), and the mixture stirred for 5 min. A solution of compound **26** (75 mg, 0.17

mmol) in dioxane (2.2 mL, degassed via argon sparge for 1.5 min prior to use) was added followed by phenyl boronic acid (35 mg, 0.29 mmol) and $Pd(PPh_3)_4$ (19.7 mg, 0.017 mmol). The reaction was heated to 85 °C, stirred for 16 h and cooled to rt. The reaction was diluted with water (20 mL) and extracted with DCM (2 × 20 mL). The combined organic phase was dried ($MgSO_4$) and evaporated to give a crude oil. Purification by silica gel chromatography (EtOAc/petrol, 1:9 to 1:1 as eluent) afforded the product as a clear oil (39 mg, 62%). ν_{max} /cm^{-1} (neat) 3403, 2997, 2938, 2836, 1655, 1512, 1462, 1244. ^{1}H NMR ($CDCl_3$, 400 MHz): δ 1.59 (ddd, 1H, J = 14.1, 8.7, 5.9 Hz, NCOCHCH*H*CH), 1.97 (ddd, 1H, J = 12.8, 9.6, 7.0 Hz, NCOCHCH*H*CH), 2.40 (dd, 1H, J = 12.9, 2.3 Hz, NCOHCH*H*CH), 2.69 – 2.78 (m, 2H, CHCH*H*CH and CH_2C*H*CH=C), 2.89 (app. q, 1H, J = 9.2 Hz, NCOCHC*H*CH_2), 3.06 (t, 1H, J = 9.4 Hz, NCOC*H*CH_2), 3.39 (d, 1H, J = 14.5 Hz, NCH*H*Ph), 3.80 (s, 3H, ArO*Me*), 4.27 (dd, 1H, J = 10.8, 7.5 Hz, OCNC*H*CH=CPh), 4.98 (d, 1H, J = 14.3 Hz, NCH*H*Ph), 5.65 – 5.79 (m, 3H, C*H*=CPhC*H*=C*H*), 6.87 (d, 2H, J = 8.7 Hz, 2 × CH_{Ar}), 7.16 (d, 2H, J = 8.5 Hz, 2 × CH_{Ar}), 7.28 – 7.41 (m, 5H, 5 × CH_{Ar}). ^{13}C{^{1}H} NMR ($CDCl_3$, 101 MHz): δ 32.3 (CH*C*H_2), 35.7 (NCOCH*C*H), 41.5 (NCOCH*C*H_2), 42.4 (NCOCHCHCH_2*C*H), 44.4 (N*C*H_2Ph), 48.1 (NCO*C*H), 55.3 (O*Me*), 58.1 (OCN*C*HCH=), 124.4, 124.5 (CH_2CH*C*H=*C*H), 126.6 (*C*H_{Ar}), 127.9, 127.9 (*C*H_{Ar} and C_{qAr}), 128.0 (*C*H_{Ar}), 128.4 (*C*H_{Ar}), 130.1 (*C*H_{Ar}), 136.6 (OCNCH*C*H=CPh), 141.0 (C_{qAr}), 142.8 (CH=*C*PhCH), 159.0 (C_{qAr}), 177.3 (N*C*O). HRMS (ESI$^+$-Orbitrap) m/z: [M+H]$^+$ Calcd for $C_{25}H_{25}NO_2$ 372.1965; Found 372.1961.

Compound (±)-28

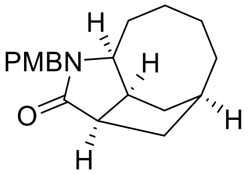

To a stirred solution of enol triflate **26** (70 mg, 0.16 mmol) in degassed MeOH (2.4 mL) at rt were added 10% Pd/C (22 mg, 0.021 mmol, 13% mol) followed by ammonium formate (55 mg, 0.85 mmol). The mixture was heated to reflux and stirred for 1 h. The mixture was cooled to rt, diluted with DCM (3 mL) and filtered through Celite. The solids were washed with DCM to give a clear filtrate. The filtrate was evaporated to give an oil. The crude product was put back under hydrogenation with degassed MeOH (2.4 mL) at reflux, with Pd/C (22 mg, 0.021 mmol, 13% mol) and ammonium formate (31.2 mg, 0.48 mmol). After refluxing for 1 h the mixture was cooled to rt, diluted with EtOAc (10 mL), filtered through Celite and the solids washed with EtOAc. The filtrate was evaporated to afford the title compound **28** (64 mg, 100%) as a clear oil. ν_{max} /cm^{-1} (neat) 2923, 2863, 1669, 1611, 1511, 1454, 1439, 1304, 1241. ^{1}H NMR ($CDCl_3$, 400 MHz): δ 0.70 (dt, 1H, J = 15.4, 9.7 Hz, CH*H*), 1.01 – 1.29 (m, 2H, 2 × CH*H*), 1.44 – 1.60 (m, 2H, 2 × CH*H*), 1.63 – 1.76 (m, 2H, 2 × CH*H*), 1.78 – 2.04 (m,

5H, CH_2 and 3 × CH*H*), 2.10 (tt, 1H, J = 9.8, 6.1 Hz, NCOCHCHCH$_2$C*H*), 2.74 (app. q, 1H, J = 10.1 Hz, NCOCHC*H*CH$_2$), 2.98 (app. t, 1H, J = 10.2 Hz, NCOC*H*CH), 3.52 (d, 1H, J = 14.5 Hz, NCH*H*Ar), 3.72 – 3.80 (m, 4H, ArO*Me* and OCNC*H*CH$_2$CH$_2$), 5.15 (d, 1H, J = 14.5 Hz, NCH*H*Ph), 6.82 (d, 2H, J = 8.4 Hz, 2 × CH_{Ar}), 7.16 (d, 2H, J = 8.4 Hz, 2 × CH_{Ar}). ^{13}C{^{1}H} NMR (CDCl$_3$, 101 MHz): δ 24.2 (*C*H$_2$), 25.7 (*C*H$_2$), 27.4 (*C*H$_2$), 31.8 (*C*H$_2$), 32.2 (*C*H$_2$), 36.4 (NCOCHCHCH$_2$*C*H), 36.5 (NCOCH*C*HCH$_2$CH), 37.4 (*C*H$_2$), 44.4 (N*C*H$_2$Ar), 47.0 (NCO*C*HCHCH$_2$), 55.4 (ArO*Me*), 56.9 (OCN*C*HCH$_2$CH$_2$), 114.0 (*C*H$_{Ar}$), 128.6 (*C*$_{qAr}$), 129.7 (*C*H$_{Ar}$), 159.1 (*C*$_{qAr}$), 178.6 (N*C*O). HRMS (ESI$^+$-Orbitrap) m/z: [M+H]$^+$ Calcd for $C_{19}H_{26}NO_2$ 300.1964; Found 300.1960.

7. Crystallographic studies

Single crystal diffraction data were collected at 150 K on a XtaLAB Synergy HyPix-Arc 100 diffractometer using copper radiation ($\lambda_{CuK\alpha}$ = 1.54184 Å) equipped with an Oxford Cryosystems CryostreamPlus open-flow N_2 cooling device. Intensities were corrected for absorption using a multifaceted crystal model created by indexing the faces of the crystal for which data were collected.[7] Cell refinement, data collection and data reduction were undertaken via the software CrysAlisPro.[8]

All structures were solved using XT[9] and refined by XL[10] using the Olex2 interface.[11] All non-hydrogen atoms were refined anisotropically and hydrogen atoms were positioned with idealised geometry. The displacement parameters of the hydrogen atoms were constrained using a riding model with $U_{(H)}$ set to be an appropriate multiple of the U_{eq} value of the parent atom.

Crystal structure determination of 8/10a ($C_{22}H_{29}NO_3$) (thermal ellipsoids are set at a 50% probability level)

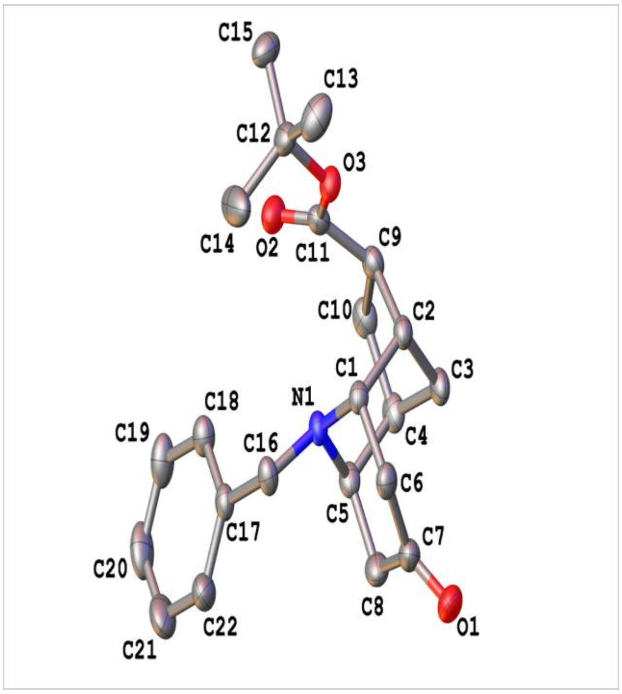

Table 1: Crystal data and structure refinement for 8/10a.

Identification code	jkpw002_fa
Empirical formula	$C_{22}H_{29}NO_3$
Formula weight	355.46
Temperature/K	150.0(2)
Crystal system	triclinic
Space group	P-1
a/Å	11.2357(2)
b/Å	11.72440(10)
c/Å	15.6163(3)
α/°	94.3220(10)
β/°	109.485(2)
γ/°	94.0820(10)
Volume/Å^3	1923.65(6)
Z	4
ρ_{calc}g/cm^3	1.227
μ/mm^{-1}	0.641
F(000)	768.0
Crystal size/mm^3	0.27 × 0.19 × 0.13
Radiation	Cu Kα (λ = 1.54184)
2Θ range for data collection/°	7.602 to 154.046
Index ranges	$-13 \le h \le 13$, $-14 \le k \le 14$, $-18 \le l \le 19$
Reflections collected	45092
Independent reflections	7463 [R_{int} = 0.0282, R_{sigma} = 0.0166]
Data/restraints/parameters	7463/0/476
Goodness-of-fit on F^2	1.052
Final R indexes [I>=2σ (I)]	R_1 = 0.0344, wR_2 = 0.0872
Final R indexes [all data]	R_1 = 0.0379, wR_2 = 0.0899
Largest diff. peak/hole / e Å^{-3}	0.22/-0.17

Crystal structure determination of 22 ($C_{19}H_{22}ClNO_3$) (thermal ellipsoids are set at a 50% probability level)

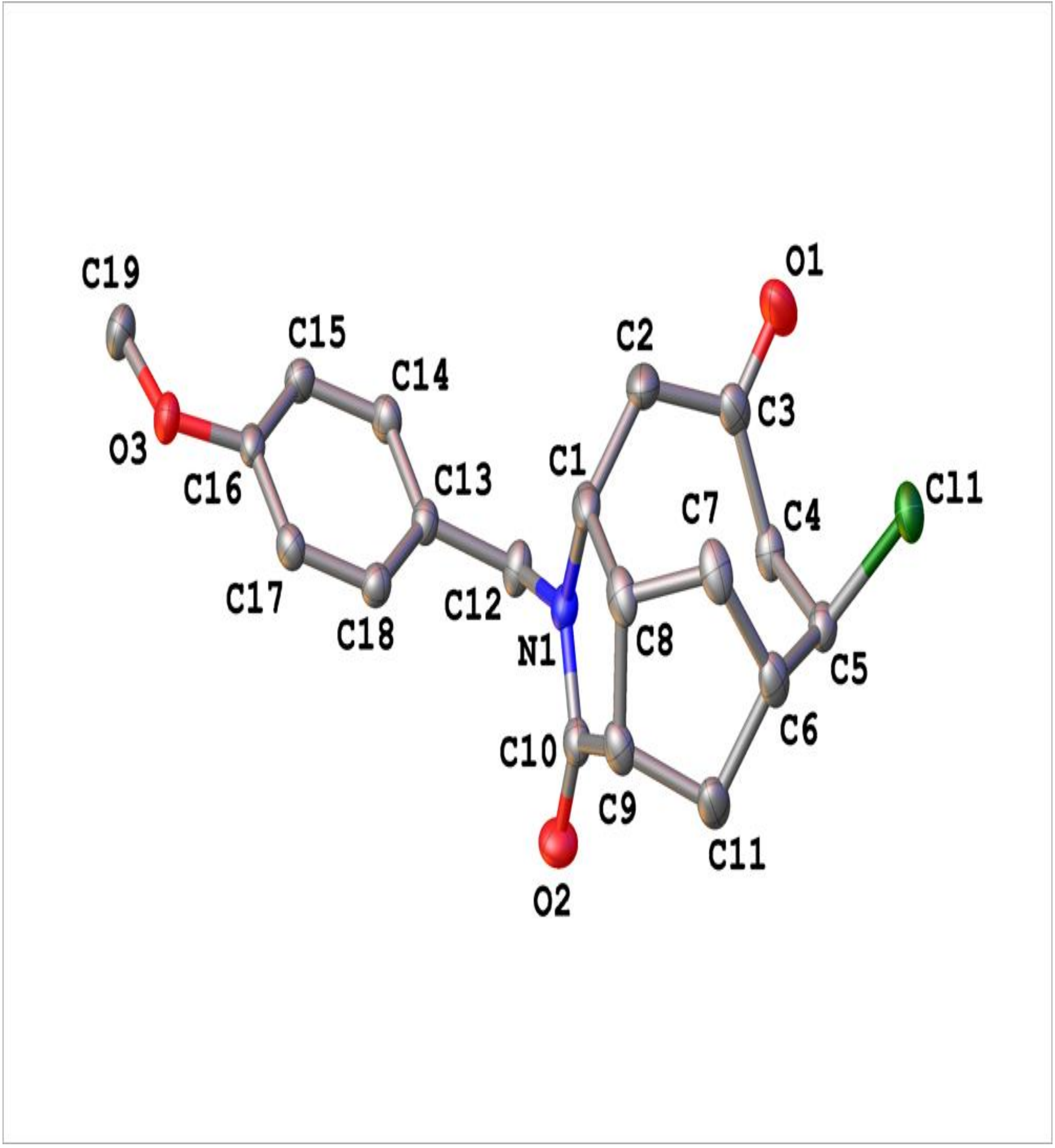

Table 2: Crystal data and structure refinement for 22.

Identification code	jkpw004_fa
Empirical formula	$C_{19}H_{22}ClNO_3$
Formula weight	347.82
Temperature/K	150.0(2)
Crystal system	triclinic
Space group	P-1
a/Å	7.61950(10)
b/Å	9.09530(10)
c/Å	13.2401(3)
α/°	99.561(2)
β/°	106.131(2)
γ/°	106.3310(10)
Volume/Å^3	815.65(3)
Z	2
ρ_{calc}g/cm^3	1.416
μ/mm^{-1}	2.218
F(000)	368.0
Crystal size/mm^3	0.23 × 0.18 × 0.08
Radiation	Cu Kα (λ = 1.54184)
2Θ range for data collection/°	7.208 to 156.27
Index ranges	$-9 \le h \le 9$, $-11 \le k \le 11$, $-16 \le l \le 14$
Reflections collected	28905
Independent reflections	3207 [R_{int} = 0.0380, R_{sigma} = 0.0177]
Data/restraints/parameters	3207/0/219
Goodness-of-fit on F^2	1.077
Final R indexes [I>=2σ (I)]	R_1 = 0.0352, wR_2 = 0.0945
Final R indexes [all data]	R_1 = 0.0365, wR_2 = 0.0954
Largest diff. peak/hole / e Å^{-3}	0.46/-0.35

Crystal structure determination of 18a ($C_{23}H_{28}F_{3}NO_{5}S$) (thermal ellipsoids are set at a 50% probability level)

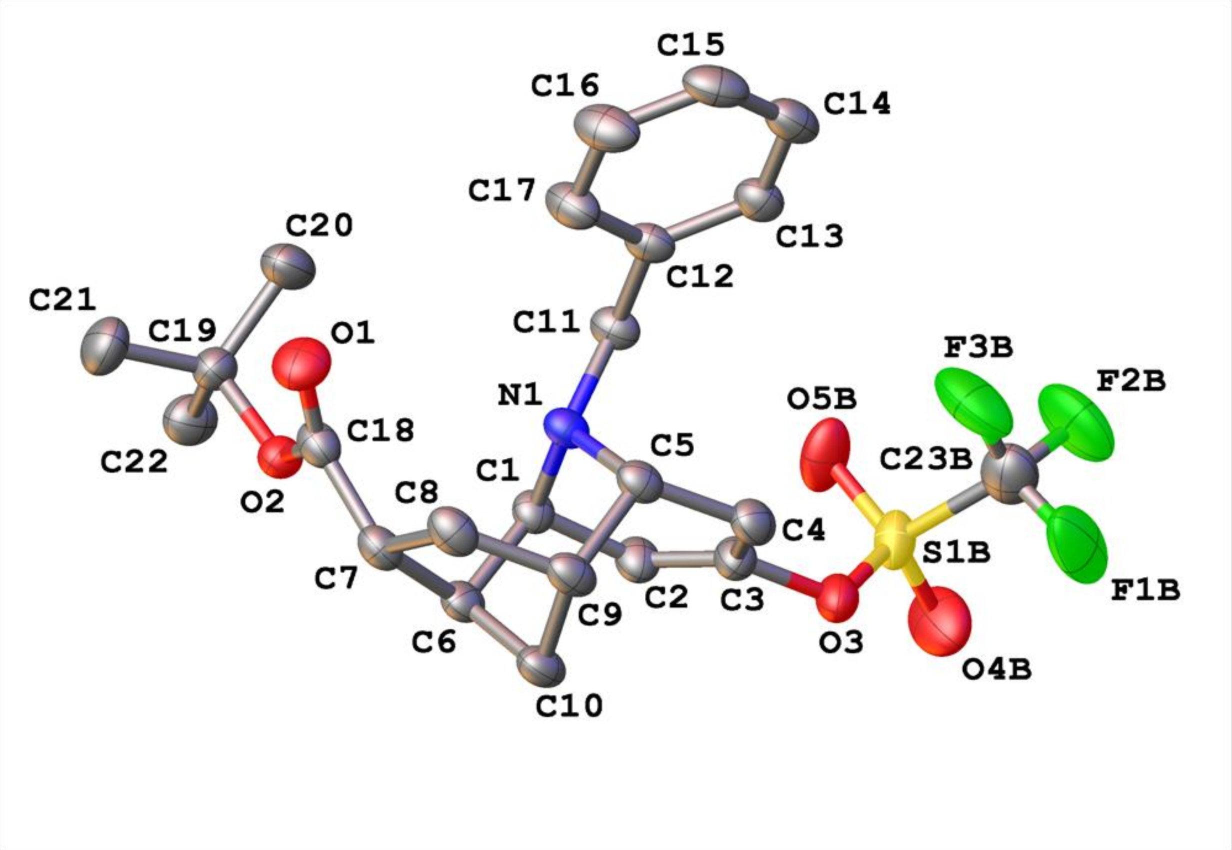

Table 3: Crystal data and structure refinement for 18a.

Identification code	jkpw005_fa
Empirical formula	$C_{23}H_{28}F_{3}NO_{5}S$
Formula weight	487.52
Temperature/K	150.0(2)
Crystal system	triclinic
Space group	P-1
a/Å	9.10590(10)
b/Å	11.0403(2)
c/Å	12.9283(2)
α/°	112.4480(10)
β/°	100.4740(10)
γ/°	95.6770(10)

Volume/Å^3	1160.77(3)
Z	2
ρ_{calc}g/cm^3	1.395
μ/mm^{-1}	1.765
F(000)	512.0
Crystal size/mm^3	0.18 × 0.15 × 0.08
Radiation	CuKα (λ = 1.54184)
2Θ range for data collection/°	7.62 to 154.5
Index ranges	$-10 \leq h \leq 11$, $-13 \leq k \leq 13$, $-15 \leq l \leq 15$
Reflections collected	41371
Independent reflections	4614 [R_{int} = 0.0264, R_{sigma} = 0.0130]
Data/restraints/parameters	4614/540/364
Goodness-of-fit on F^2	1.075
Final R indexes [I>=2σ (I)]	R_1 = 0.0335, wR_2 = 0.0880
Final R indexes [all data]	R_1 = 0.0375, wR_2 = 0.0908
Largest diff. peak/hole / e Å^{-3}	0.20/-0.29

8. References.

[1] Stille, J. R.; Santarsiero, B. D.; Grubbs, R. H. Rearrangement of bicyclo[2.2.1]heptane ring systems by titanocene alkylidene complexes to bicyclo[3.2.0]heptane enol ethers. Total synthesis of (+-)-DELTA9(12)-capnellene *J. Org, Chem*. **1990**, *55*, 843-862.
[2] Hatano, M.; Kamiya. S.; K. Ishihara, K. In situ generated “lanthanum(iii) nitrate alkoxide” as a highly active and nearly neutral transesterification catalyst. *Chem. Commun*. **2012**, *48*, 9465-9467.
[3] Vrabel, M.; Kölle, P.; Brunner, K. N.; Gattner, M. J.; López-Carrillo, V.; de Vivie-Riedle, R.; Carell, T. Norbornenes in Inverse Electron-Demand Diels–Alder Reactions. *Chem. Eur. J*. **2013**, *19*, 13309-13312.
[4] Abraham, R. J.; Fisher, Substituent chemical shifts in NMR. 3-Carbonitrile SCS in rigid molecules. *J. Magn. Reson. Chem*. **1986**, *24*, 451-459.
[5] Ho, T.-L.; Kung, L.-R.; Chein, R.-J. Total Synthesis of (±)-2-Isocyanoallopupukeanane. *J. Org. Chem*. **2000**, *65*, 5774-5779.
[6] Mathieu, B.; Ghosez, L. Trimethylsilyl bis(trifluoromethanesulfonyl)imide as a tolerant and environmentally benign Lewis acid catalyst of the Diels–Alder reaction. *Tetrahedron*, **2002**, *58*, 8219-8226.
[7] Clark R. C.; J. S. Reid, J. S. The analytical calculation of absorption in multifaceted crystals. *Acta Cryst.,* **1995**, *A51*, 887
[8] CrysAlisPro, Rigaku Oxford Diffraction, Tokyo, Japan.
[9] Sheldrick, G.M. SHELXT – Integrated space-group and crystal-structure determination. *Acta Crystallogr., Sect. A: Found. Crystallogr.* **2015,** *71*, 3-8.
[10] Sheldrick, G.M. A short history of SHELX. *Acta Crystallogr., Sect. A: Found. Crystallogr.* **2008,** *64*, 112-122.
[11] Dolomanov, O.V.; Bourhis, L.J.; Gildea, R.J.; Howard, J.A.K.; Puschmann, H. OLEX2: a complete structure solution, refinement and analysis program. *J. Appl. Cryst.* **2009,** *42*, 339-341.

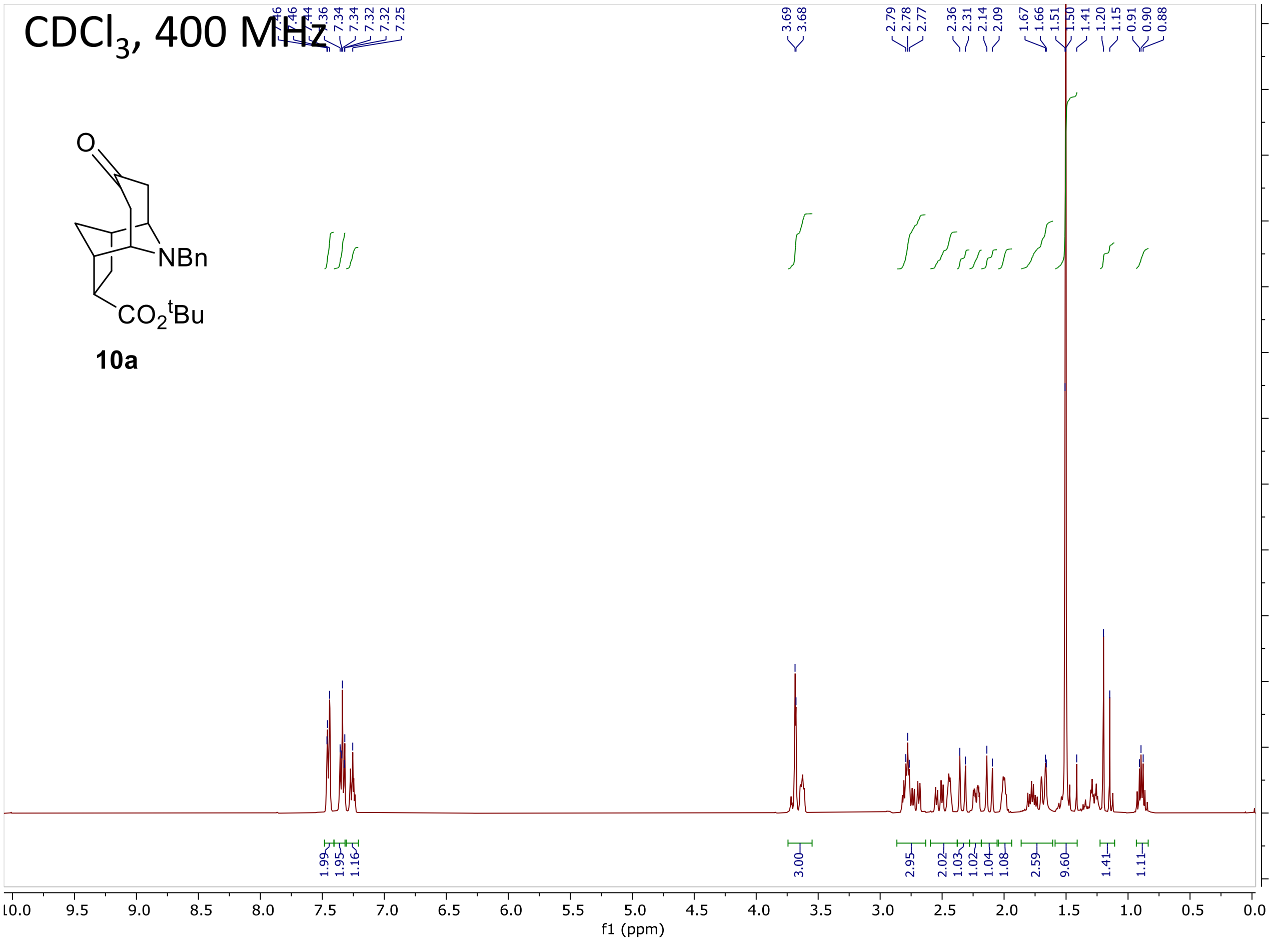
CDCl3, 400 MHz
O
NBn
CO2tBu
10a
3.69
3.68
2.79
2.78
2.77
2.36
2.31
2.14
2.09
1.67
1.66
1.51
1.50
1.41
1.20
1.15
0.91
0.90
0.88
1.99
1.95
1.16
3.00
2.95
2.02
1.03
1.02
1.04
1.08
2.59
9.60
1.41
1.11
f1 (ppm)

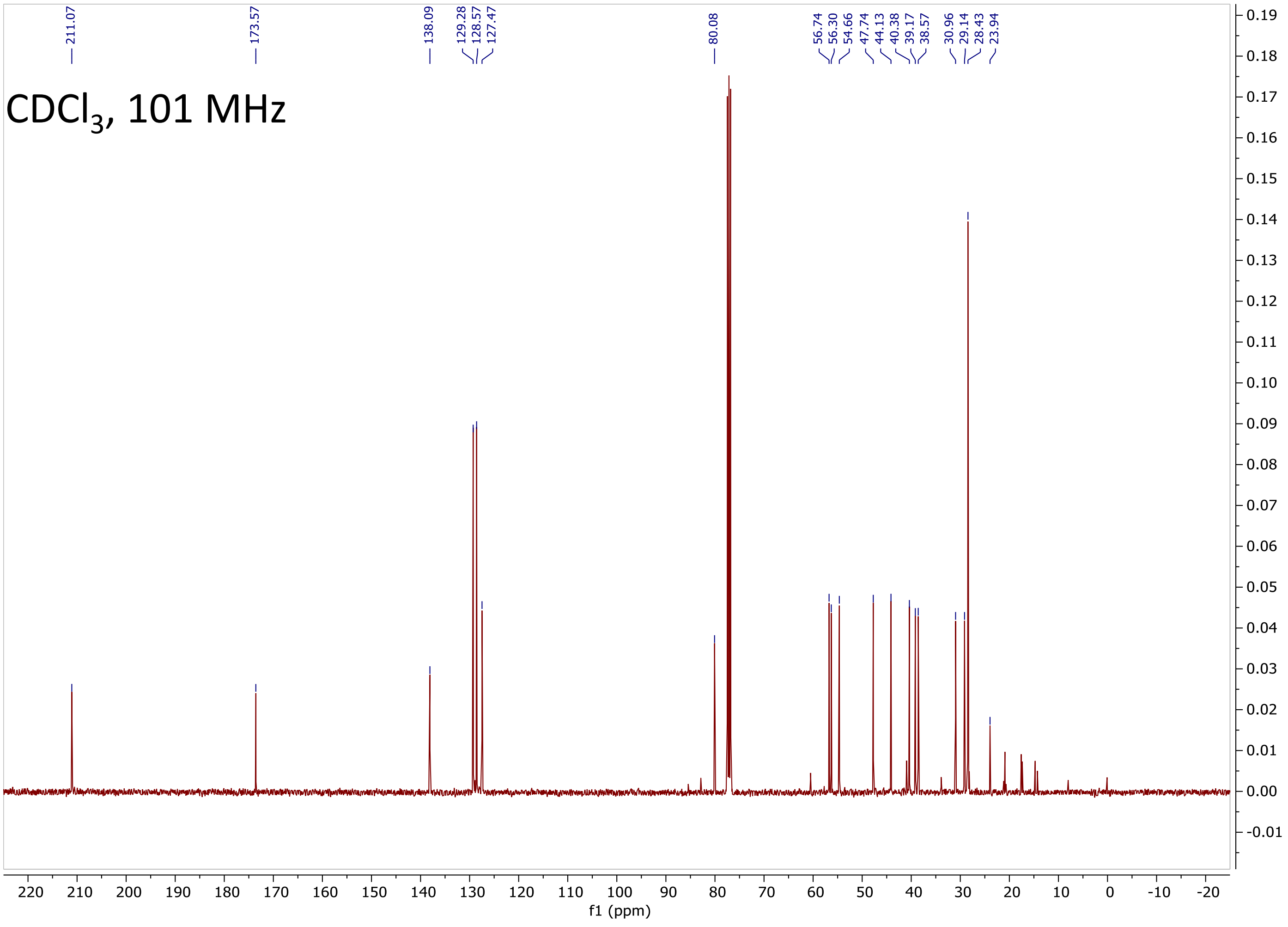
211.07
173.57
138.09
129.28
128.57
127.47
80.08
56.74
56.30
54.66
47.74
44.13
40.38
39.17
38.57
30.96
29.14
28.43
23.94
CDCl3, 101 MHz
f1 (ppm)

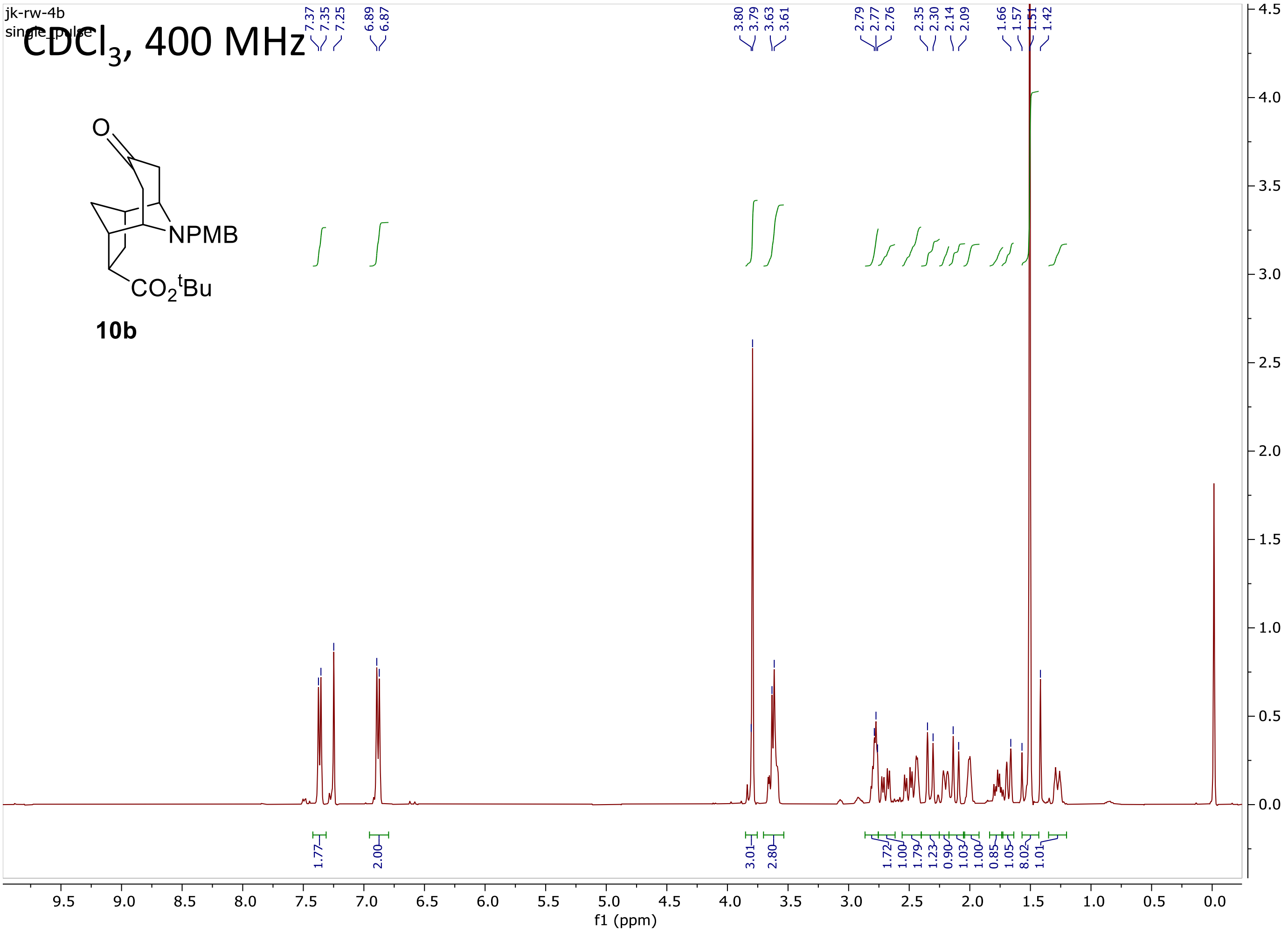
jk-rw-4b
single_pulse
CDCl$_3$, 400 MHz
O
NPMB
CO$_2$^{t}Bu
10b
7.37
7.35
7.25
6.89
6.87
3.80
3.79
3.63
3.61
2.79
2.77
2.76
2.35
2.30
2.14
2.09
1.66
1.57
1.51
1.42
1.77
2.00
3.01
2.80
1.72
1.00
1.79
1.23
0.90
1.03
1.00
0.85
1.05
8.02
1.01
f1 (ppm)

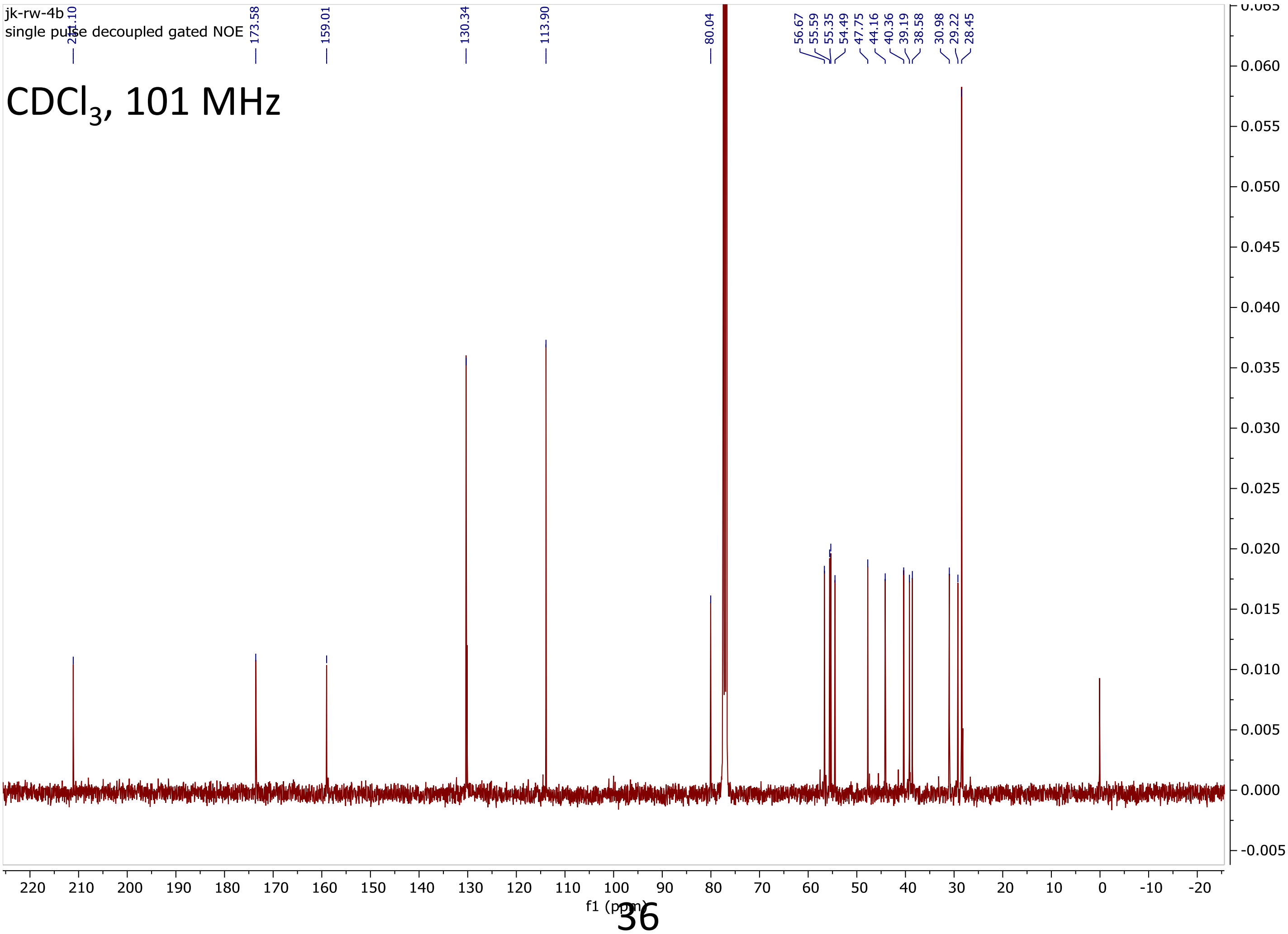
jk-rw-4b
single pulse decoupled gated NOE
CDCl$_3$, 101 MHz
211.10
173.58
159.01
130.34
113.90
80.04
56.67
55.59
55.35
54.49
47.75
44.16
40.36
39.19
38.58
30.98
29.22
28.45
f1 (ppm)

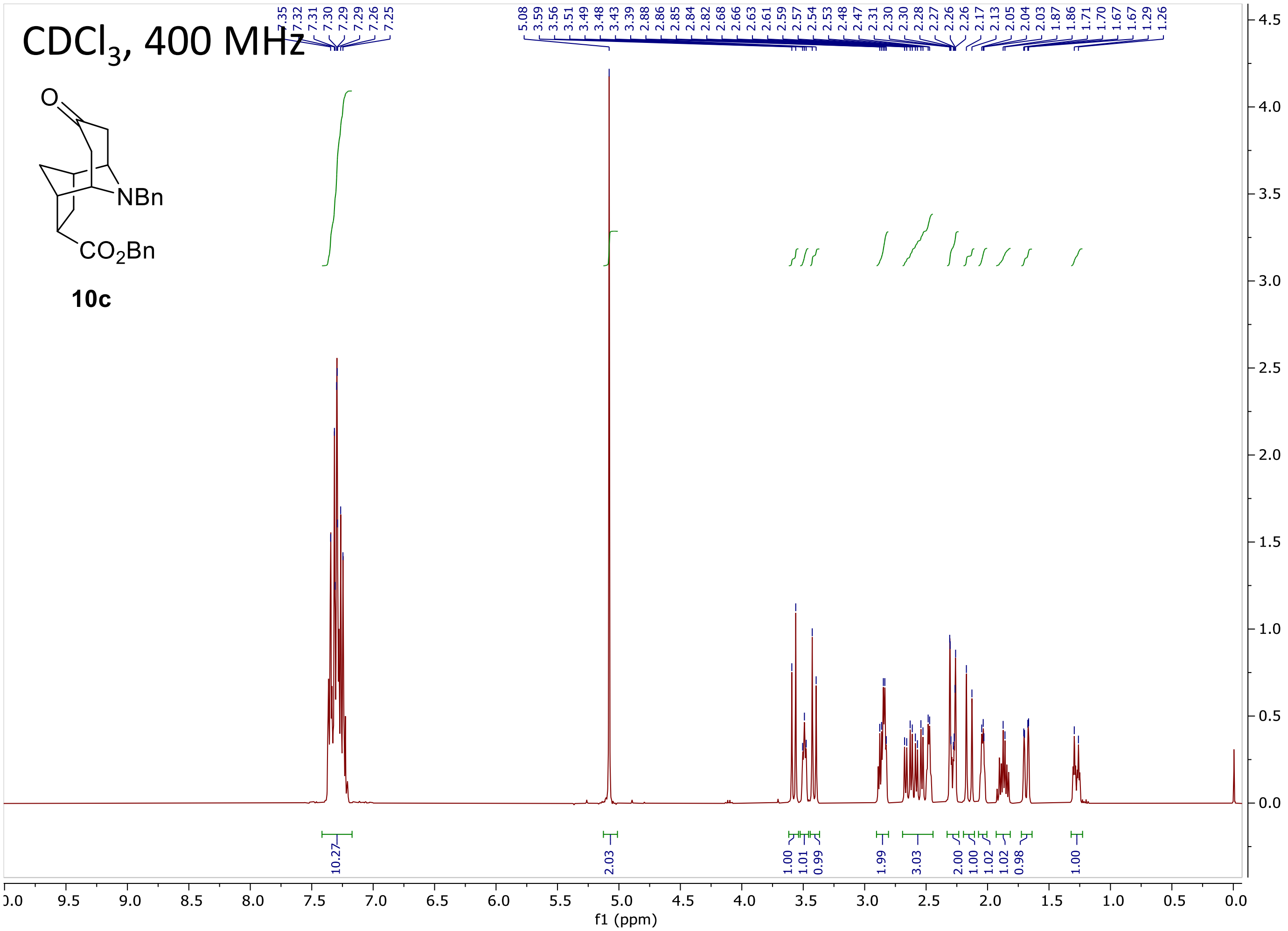
CDCl3, 400 MHz
10c
7.35 7.32 7.31 7.30 7.29 7.29 7.26 7.25
5.08 3.59 3.56 3.51 3.49 3.48 3.43 3.39 2.88 2.86 2.85 2.84 2.82 2.66 2.63 2.61 2.59 2.57 2.54 2.53 2.48 2.47 2.31 2.30 2.30 2.28 2.27 2.26 2.26 2.17 2.13 2.05 2.04 2.03 1.87 1.86 1.71 1.70 1.67 1.29 1.26
10.27 2.03 1.00 1.01 0.99 1.99 3.03 2.00 1.00 1.02 1.02 0.98 1.00
f1 (ppm)

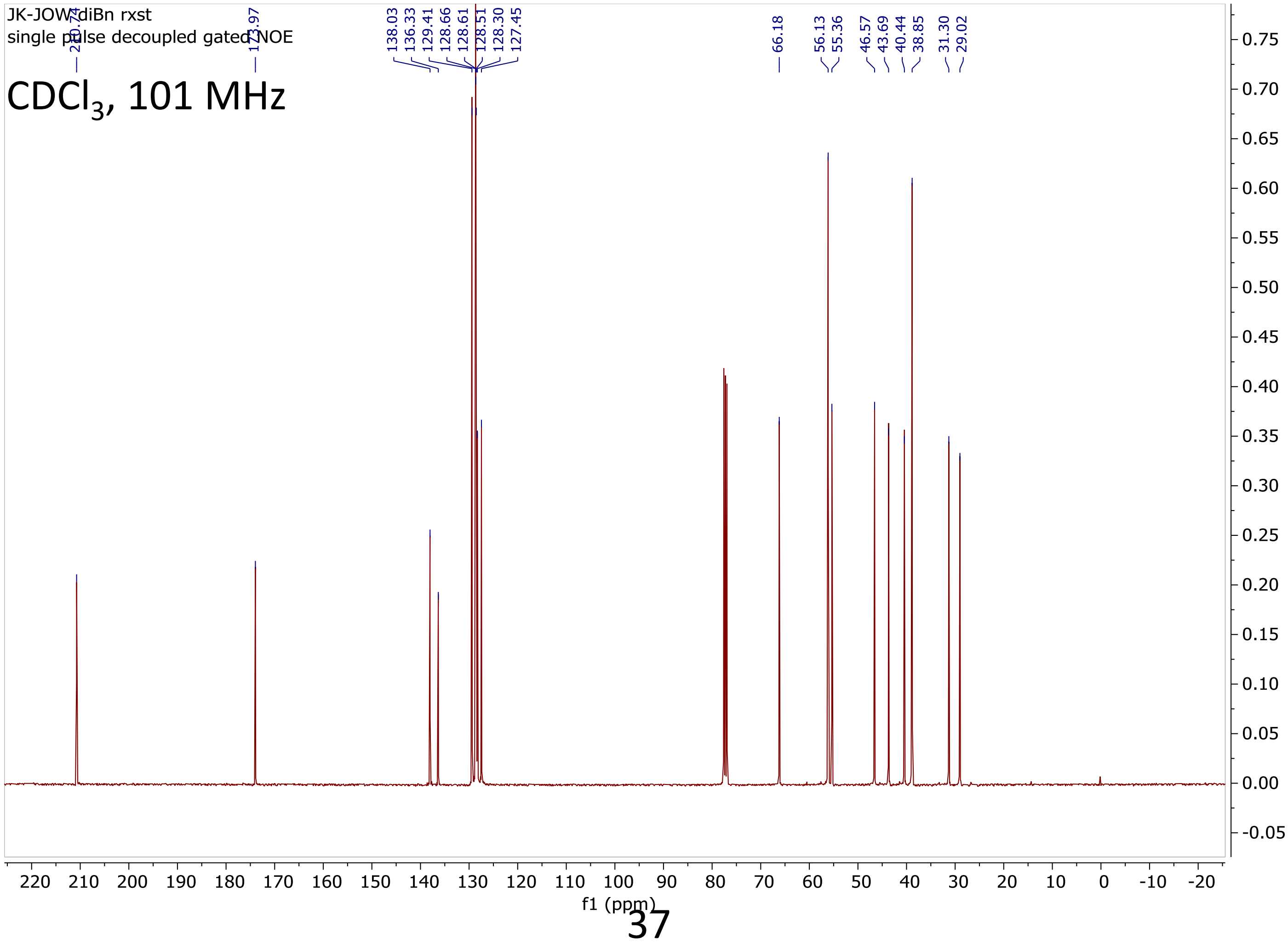
JK-JOW diBn rxst
single pulse decoupled gated NOE
CDCl3, 101 MHz
210.74 173.97 138.03 136.33 129.41 128.66 128.61 128.51 128.30 127.45 66.18 56.13 55.36 46.57 43.69 40.44 38.85 31.30 29.02
f1 (ppm)

CDCl$_3$, 400 MHz

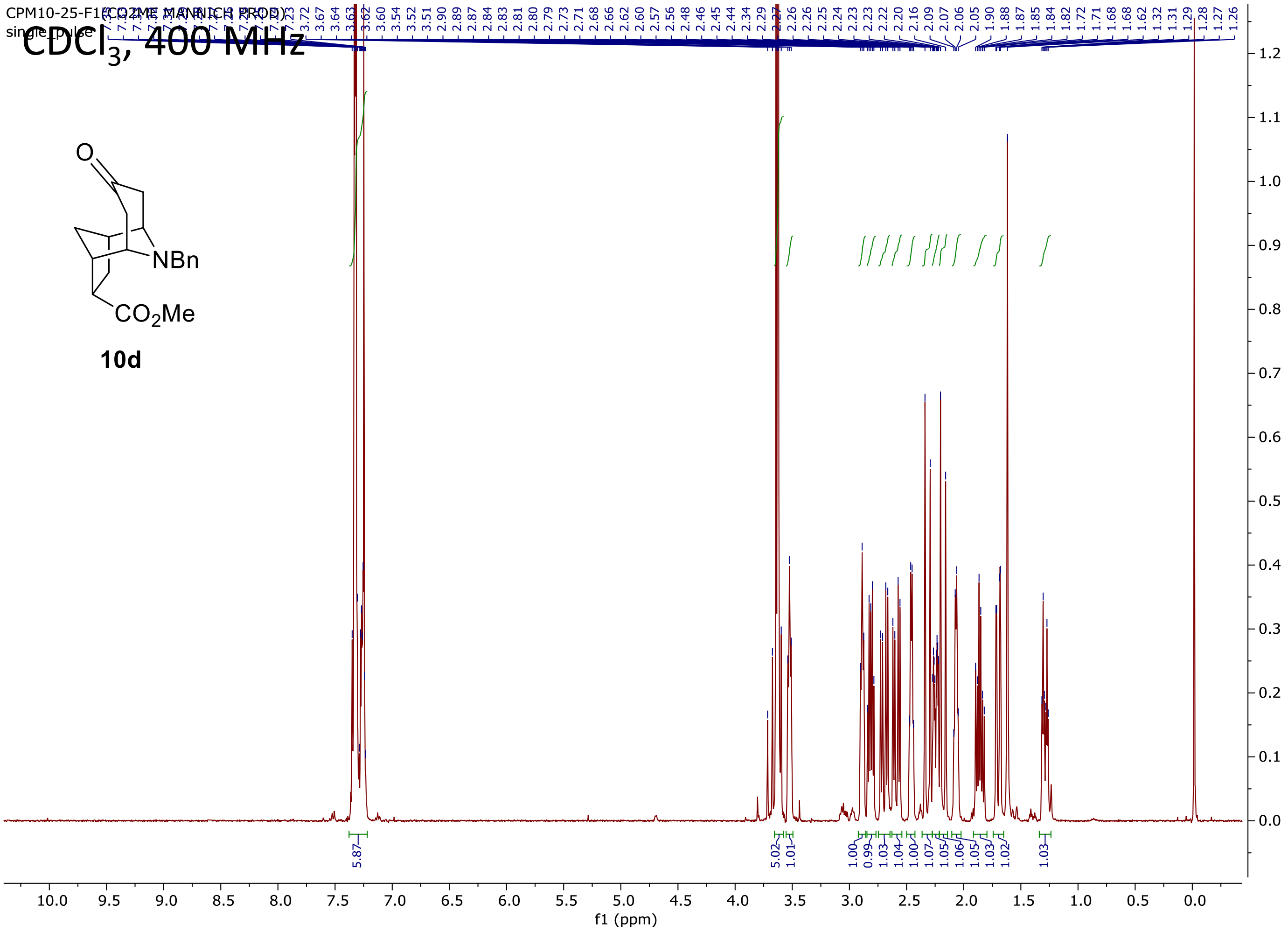

10d

CDCl$_3$, 101 MHz

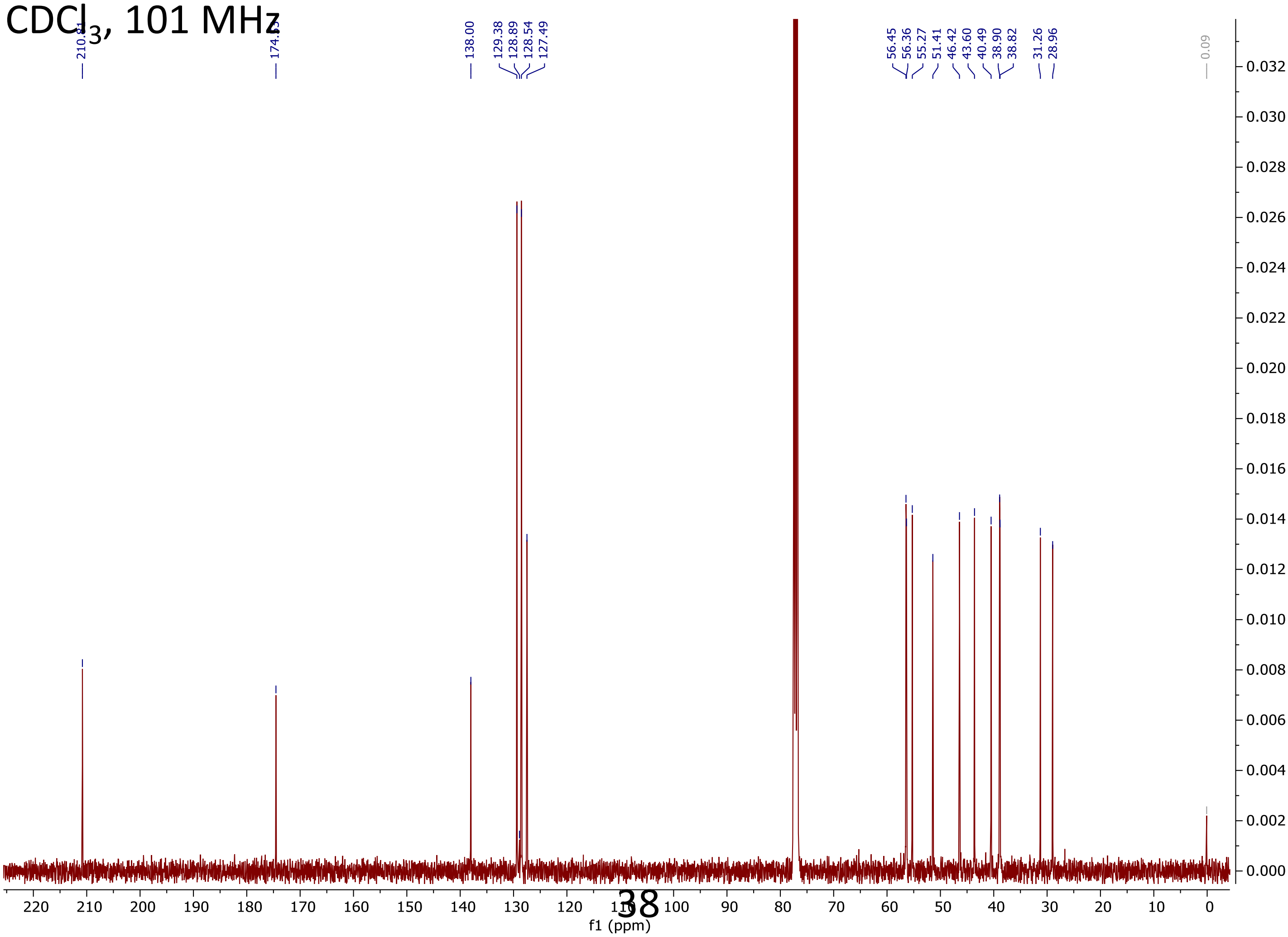

CDCl$_3$, 400 MHz

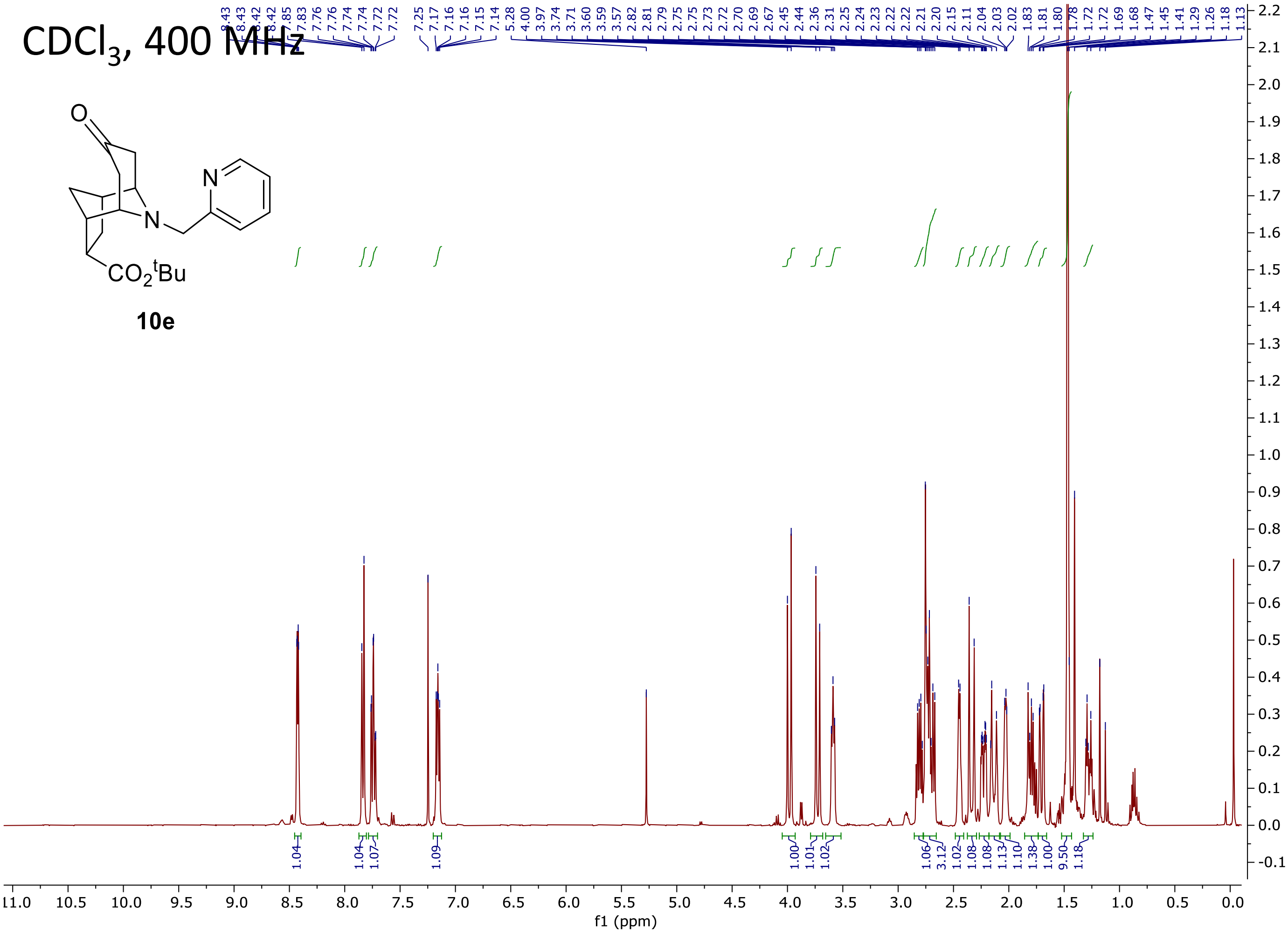

10e

CDCl$_3$, 101 MHz

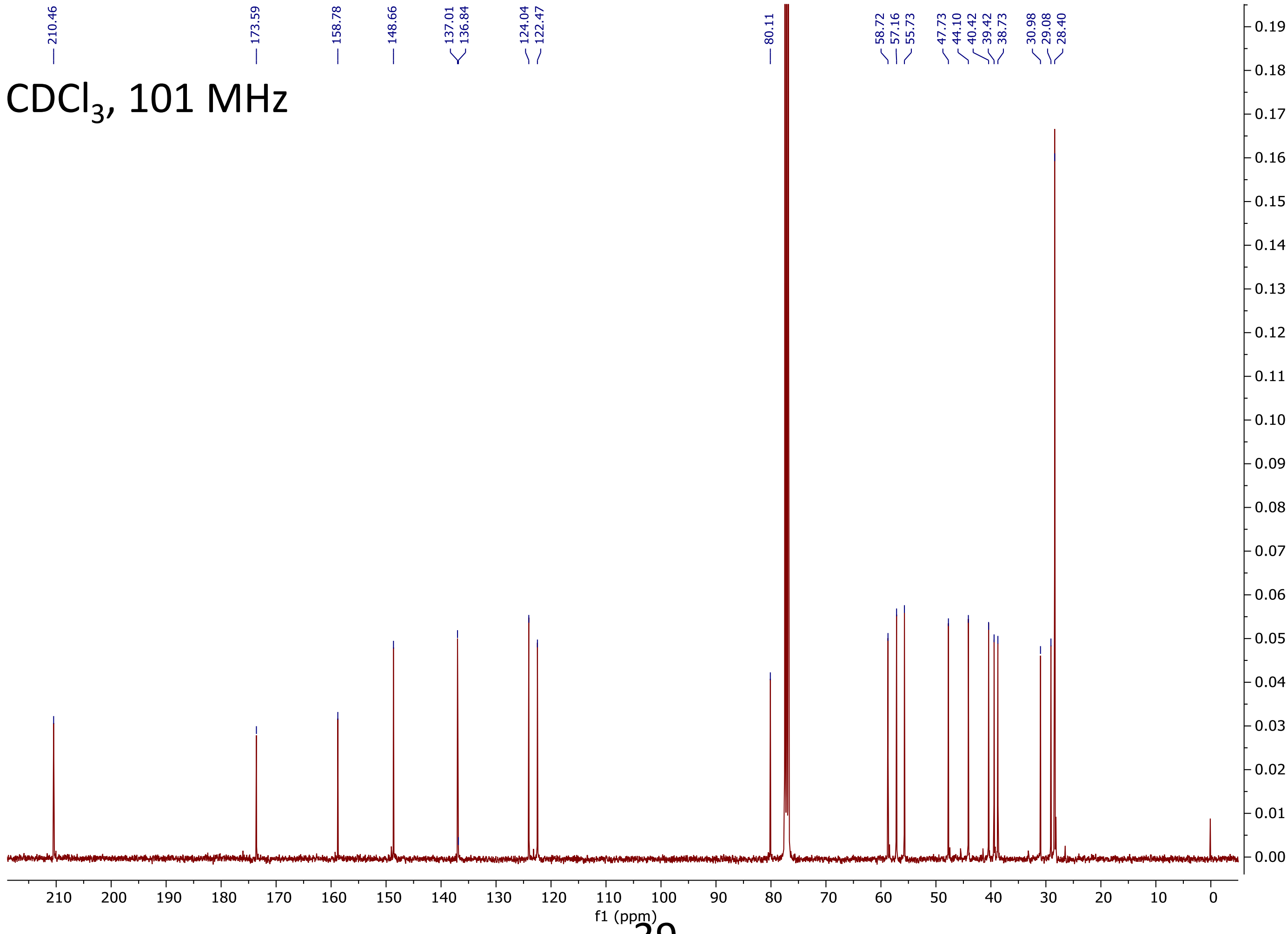

CDCl$_3$, 400 MHz

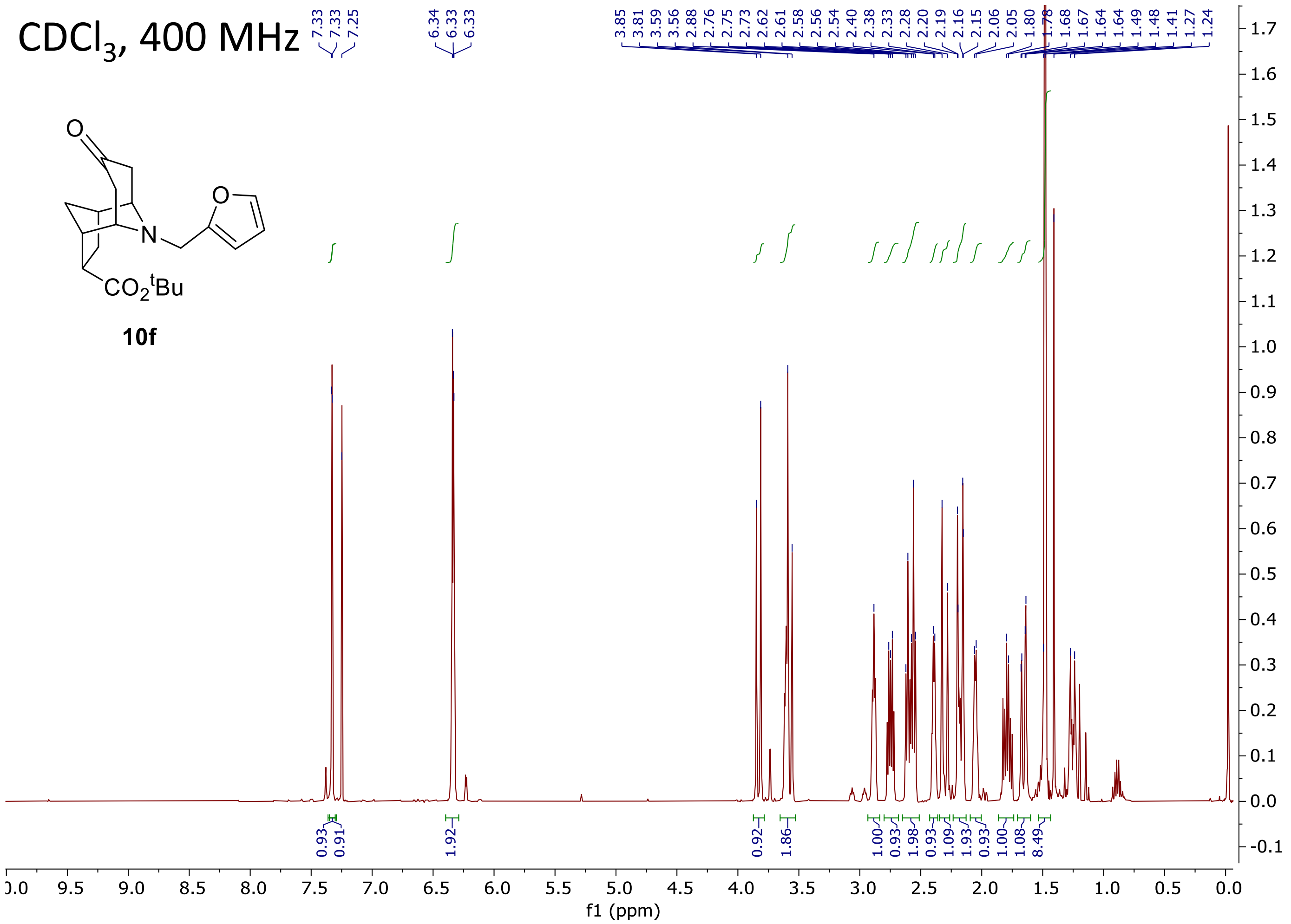

10f

CDCl$_3$, 101 MHz

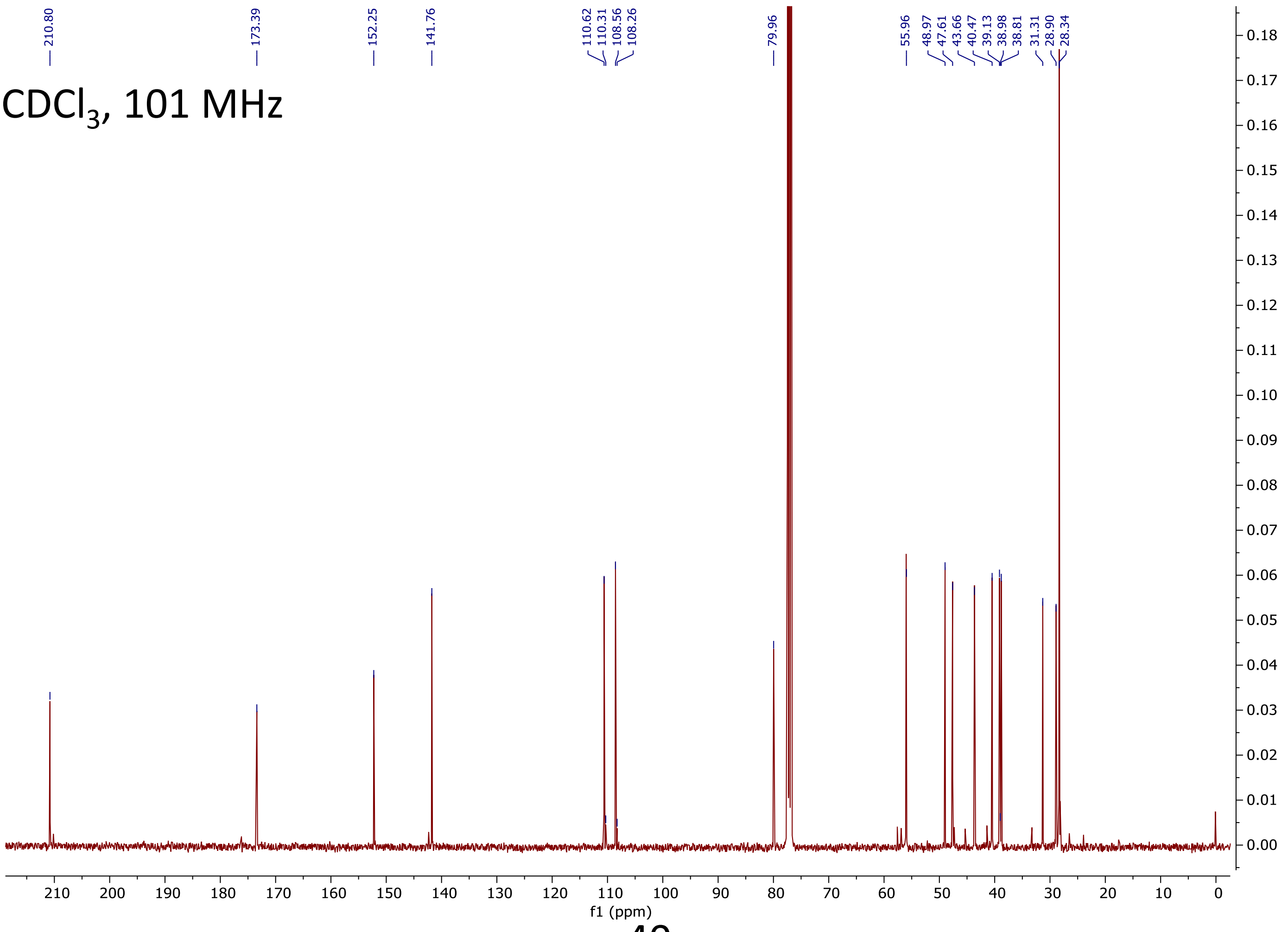

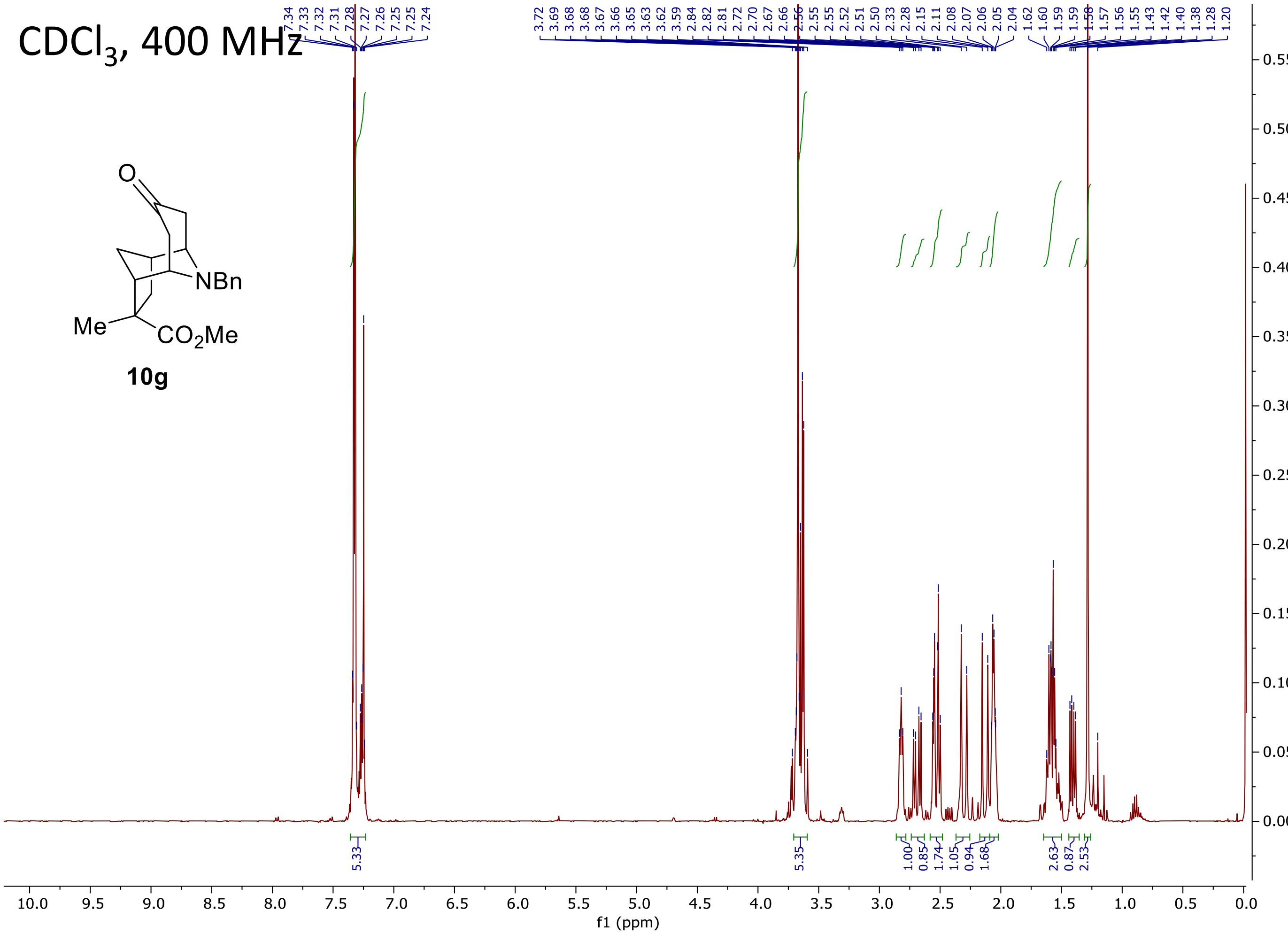
CDCl$_3$, 400 MHz
7.34 7.33 7.32 7.31 7.28 7.27 7.26 7.25 7.25 7.24
3.72 3.69 3.68 3.68 3.67 3.66 3.65 3.63 3.62 3.59 2.84 2.82 2.81 2.72 2.70 2.67 2.66 2.56 2.55 2.55 2.52 2.51 2.50 2.33 2.28 2.15 2.11 2.08 2.07 2.06 2.05 2.04 1.62 1.60 1.59 1.59 1.58 1.57 1.56 1.55 1.43 1.42 1.40 1.38 1.28 1.20
O
NBn
Me
CO$_2$Me
5.33 5.35 1.00 0.85 1.74 1.05 0.94 1.68 2.63 0.87 2.53
f1 (ppm)
10g

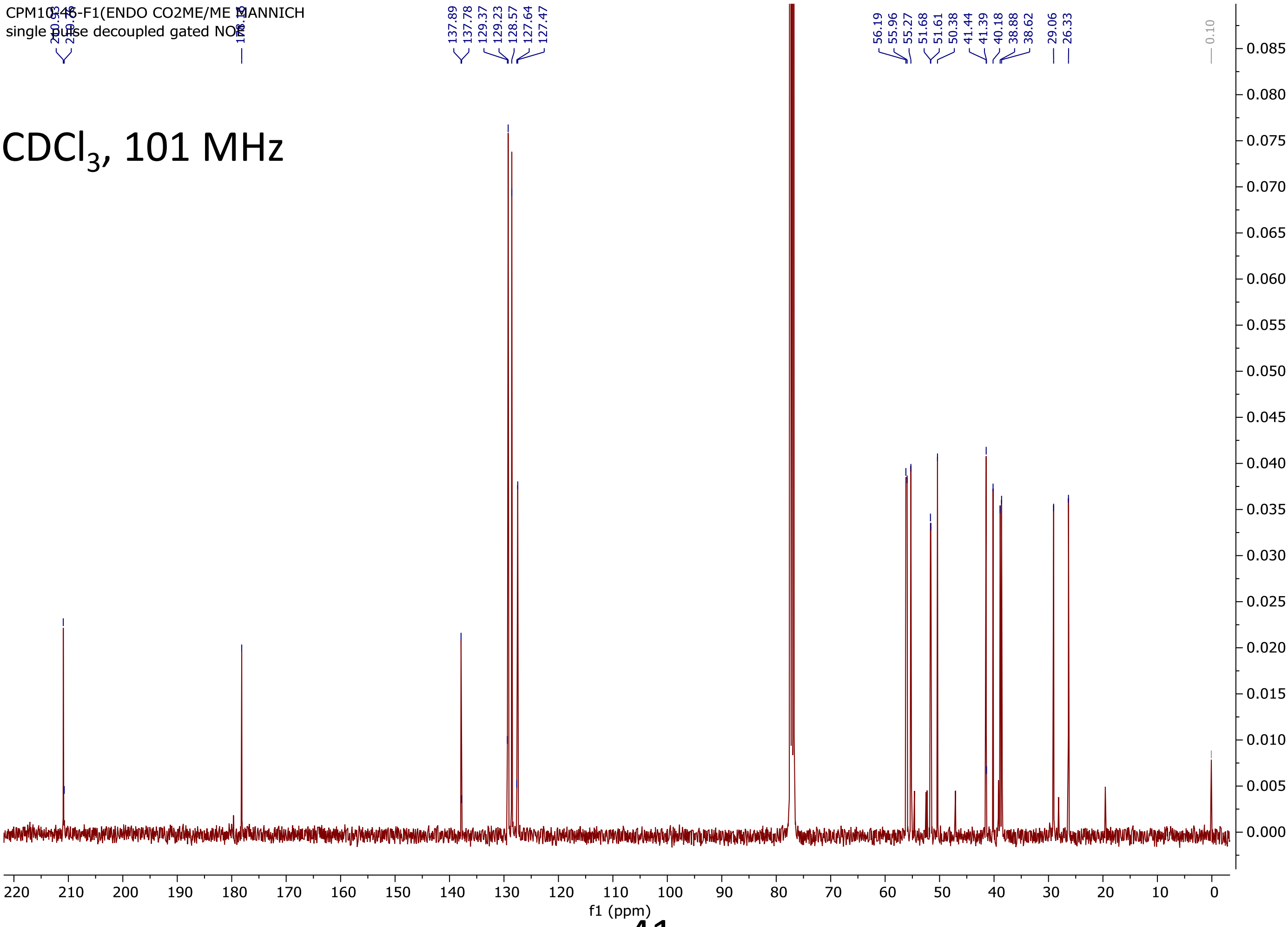
single pulse decoupled gated NOE
CDCl$_3$, 101 MHz
137.89 137.78 129.37 129.23 128.57 127.64 127.47
56.19 55.96 55.27 51.68 51.61 50.38 41.44 41.39 40.18 38.88 38.62 29.06 26.33
0.10
f1 (ppm)

^1H-^1H NOESY spectrum. $CDCl_3$, 400 MHz

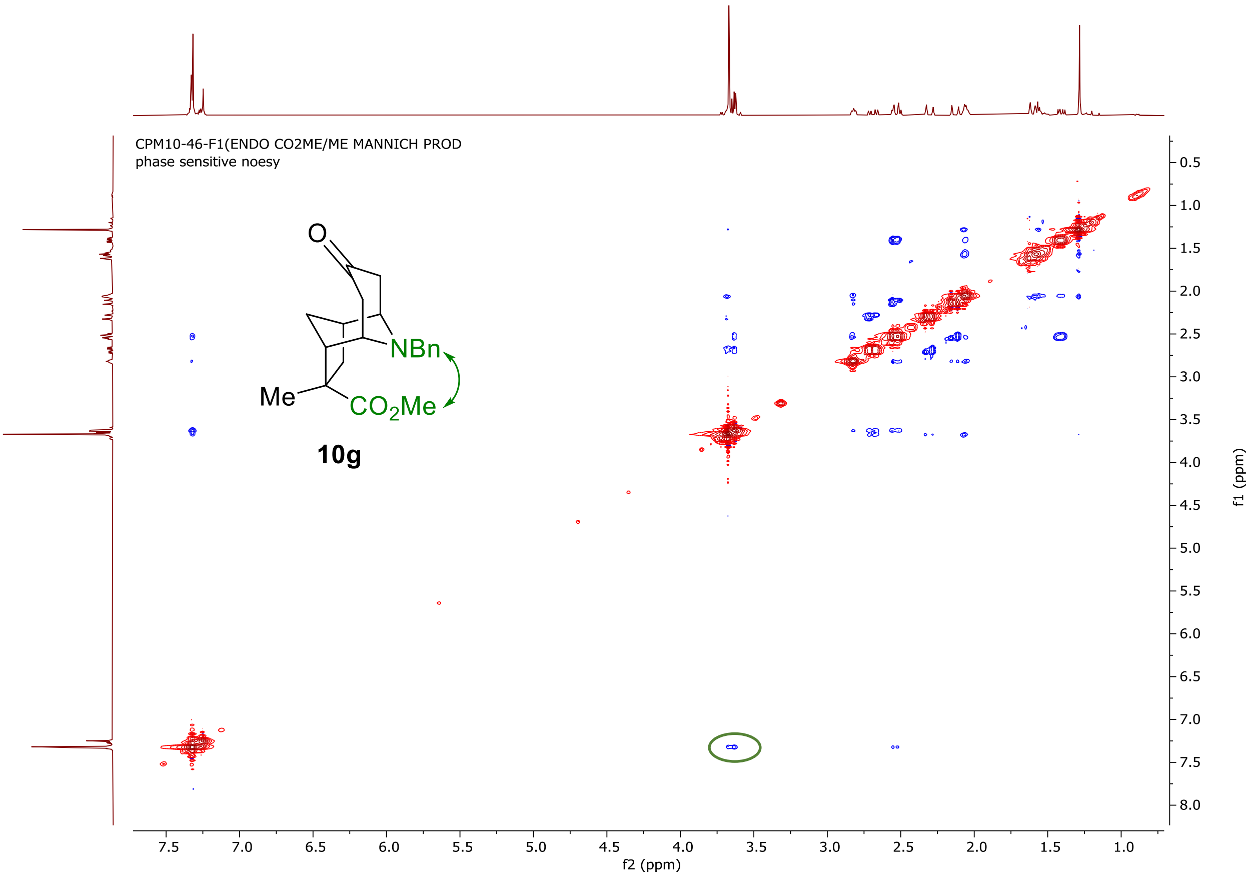

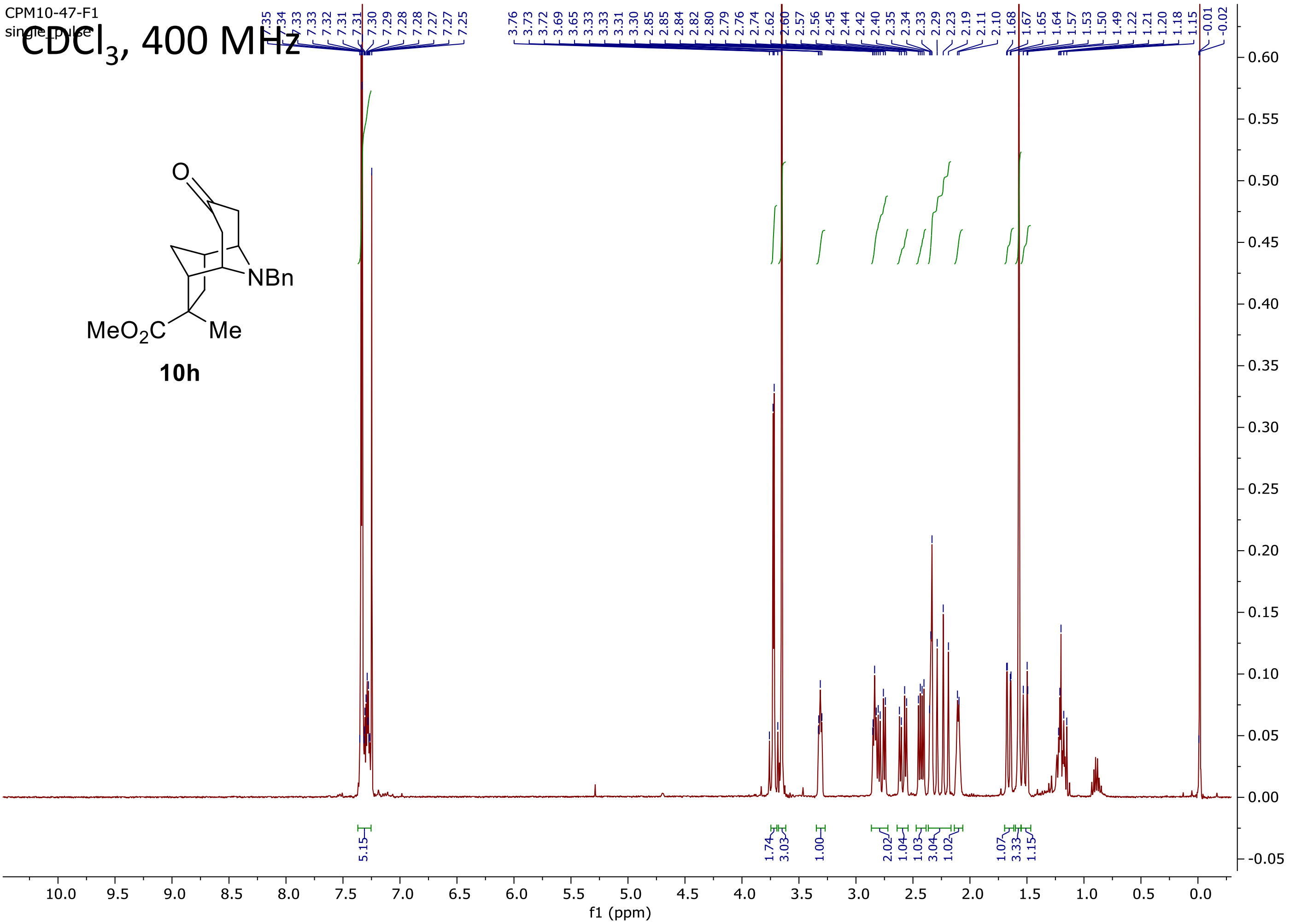
CPM10-47-F1
single pulse
CDCl$_3$, 400 MHz
7.35 7.34 7.33 7.33 7.32 7.31 7.31 7.30 7.29 7.28 7.28 7.27 7.27 7.25
3.76 3.73 3.72 3.69 3.65 3.33 3.33 3.31 3.30 2.85 2.85 2.84 2.82 2.80 2.79 2.76 2.74 2.62 2.60 2.57 2.56 2.45 2.44 2.42 2.40 2.35 2.34 2.33 2.29 2.23 2.19 2.11 2.10 1.68 1.67 1.65 1.64 1.57 1.53 1.50 1.49 1.22 1.21 1.20 1.18 1.15 -0.01 -0.02
O
NBn
MeO$_2$C
Me
10h
5.15 1.74 3.03 1.00 2.02 1.04 1.03 3.04 1.02 1.07 3.33 1.15
f1 (ppm)

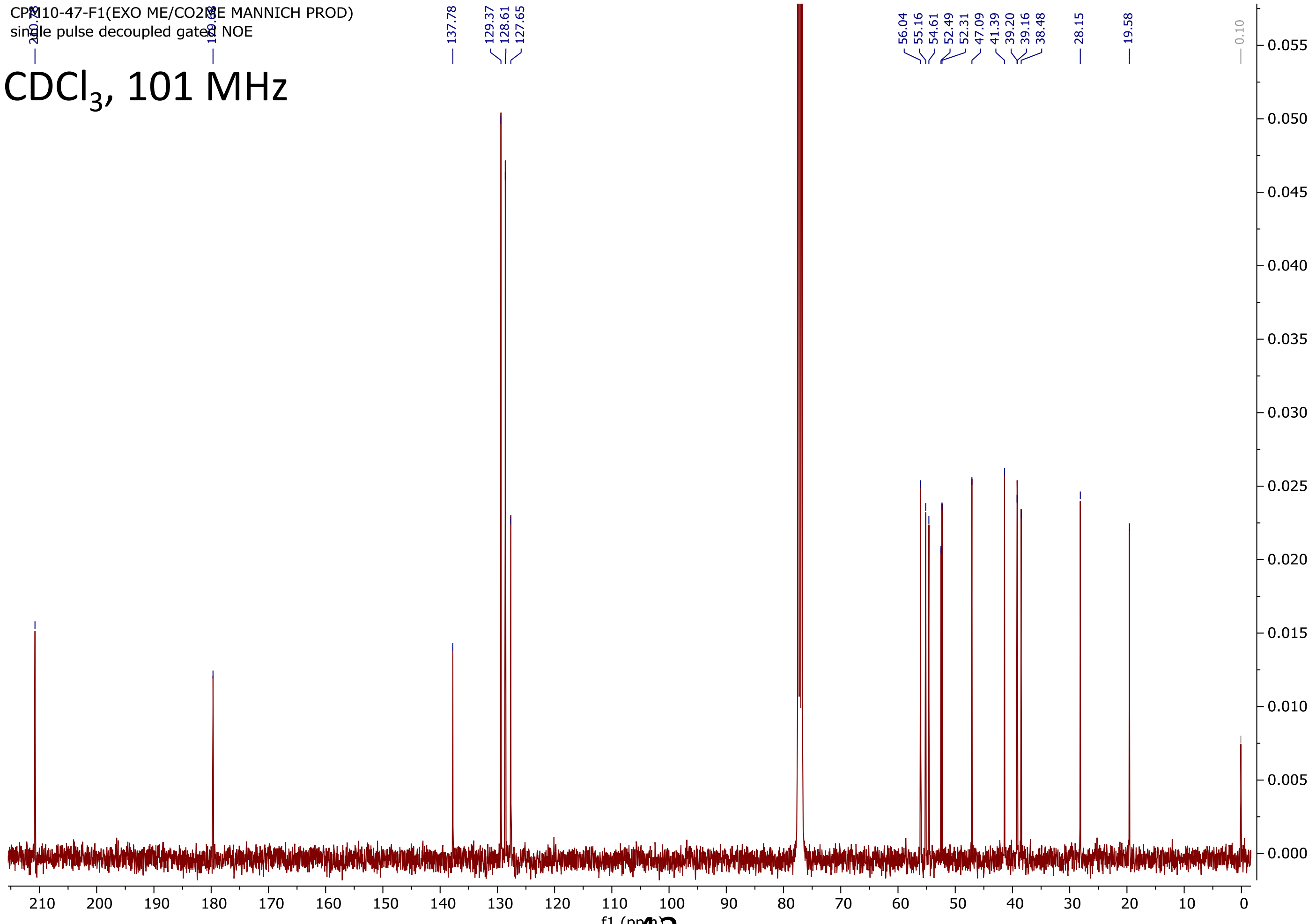
CPM10-47-F1(EXO ME/CO2ME MANNICH PROD)
single pulse decoupled gated NOE
CDCl$_3$, 101 MHz
137.78 129.37 128.61 127.65
56.04 55.16 54.61 52.49 52.31 47.09 41.39 39.20 39.16 38.48 28.15 19.58 0.10
f1 (ppm)

^1H-^1H NOESY spectrum. $CDCl_3$, 400 MHz

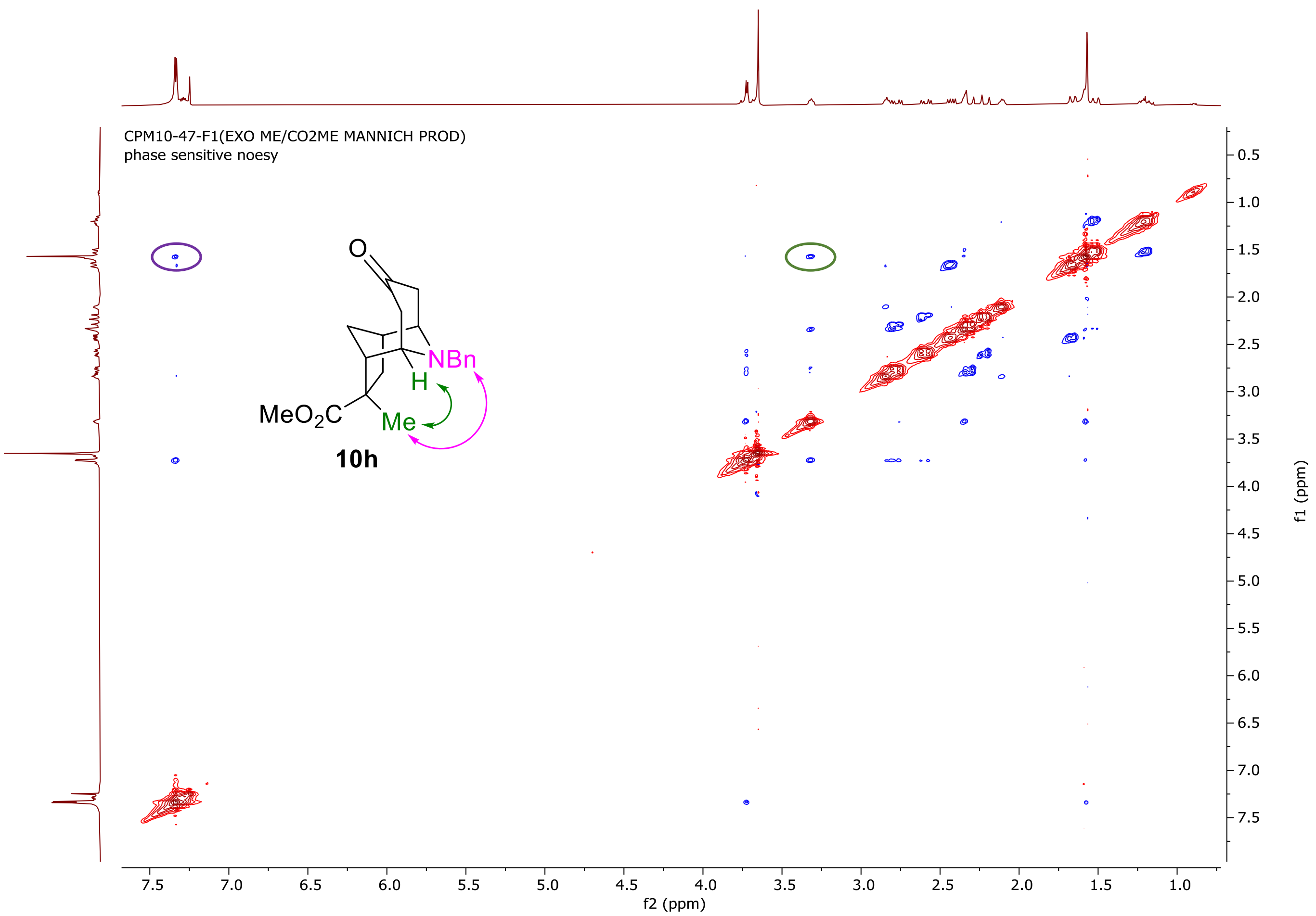

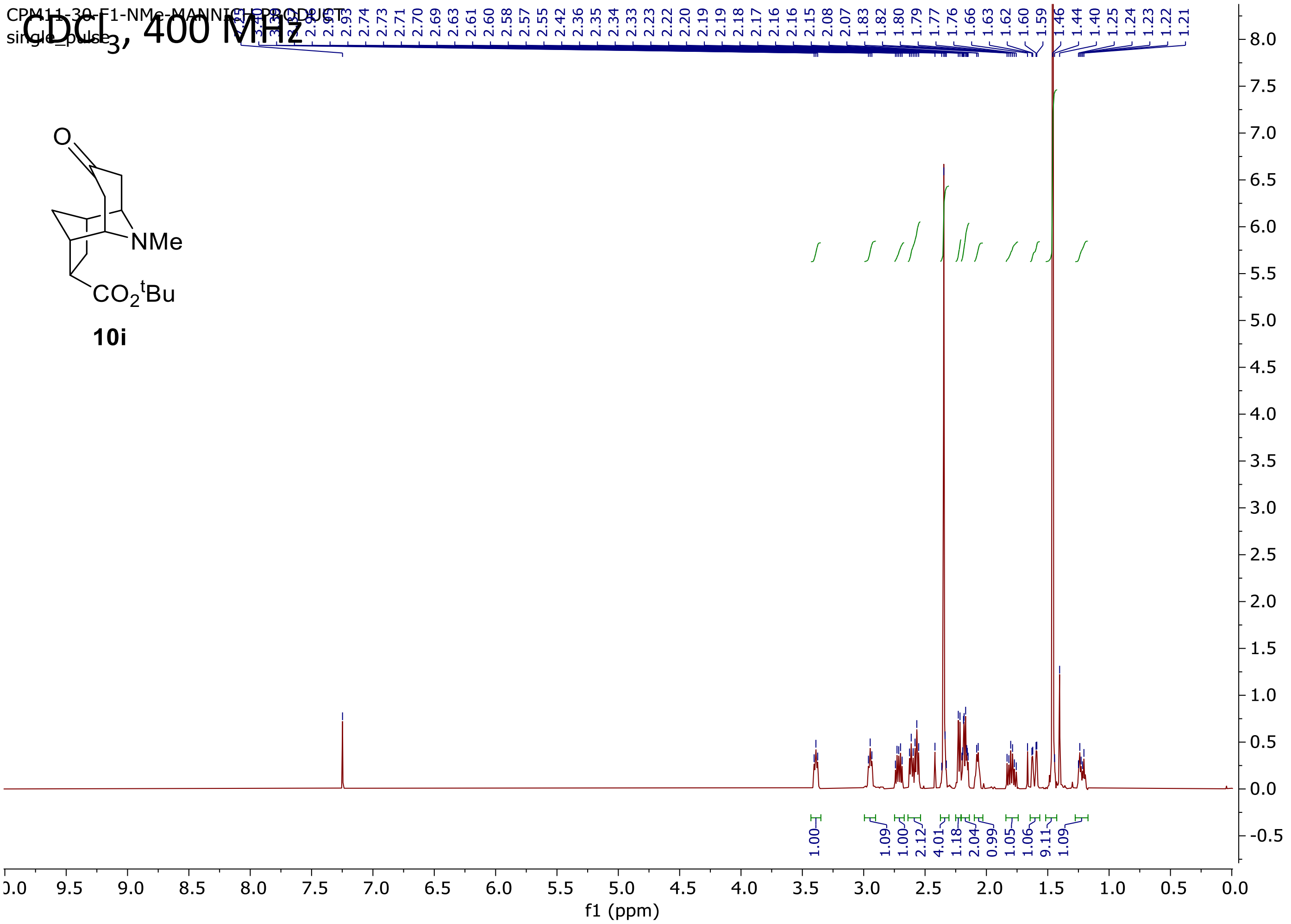
CPM11-30-F1-NMe-MANNICH PRODUCT
single_pulse
CDCl3, 400 MHz
O
NMe
CO2tBu
10i
2.93 2.74 2.73 2.71 2.70 2.69 2.63 2.61 2.60 2.58 2.57 2.55 2.42 2.36 2.35 2.34 2.33 2.23 2.22 2.20 2.19 2.19 2.18 2.17 2.16 2.16 2.15 2.08 2.07 1.83 1.82 1.80 1.79 1.77 1.76 1.66 1.63 1.62 1.60 1.59 1.46 1.44 1.40 1.25 1.24 1.23 1.22 1.21
1.00 1.09 1.00 2.12 4.01 1.18 2.04 0.99 1.05 1.06 9.11 1.09
f1 (ppm)
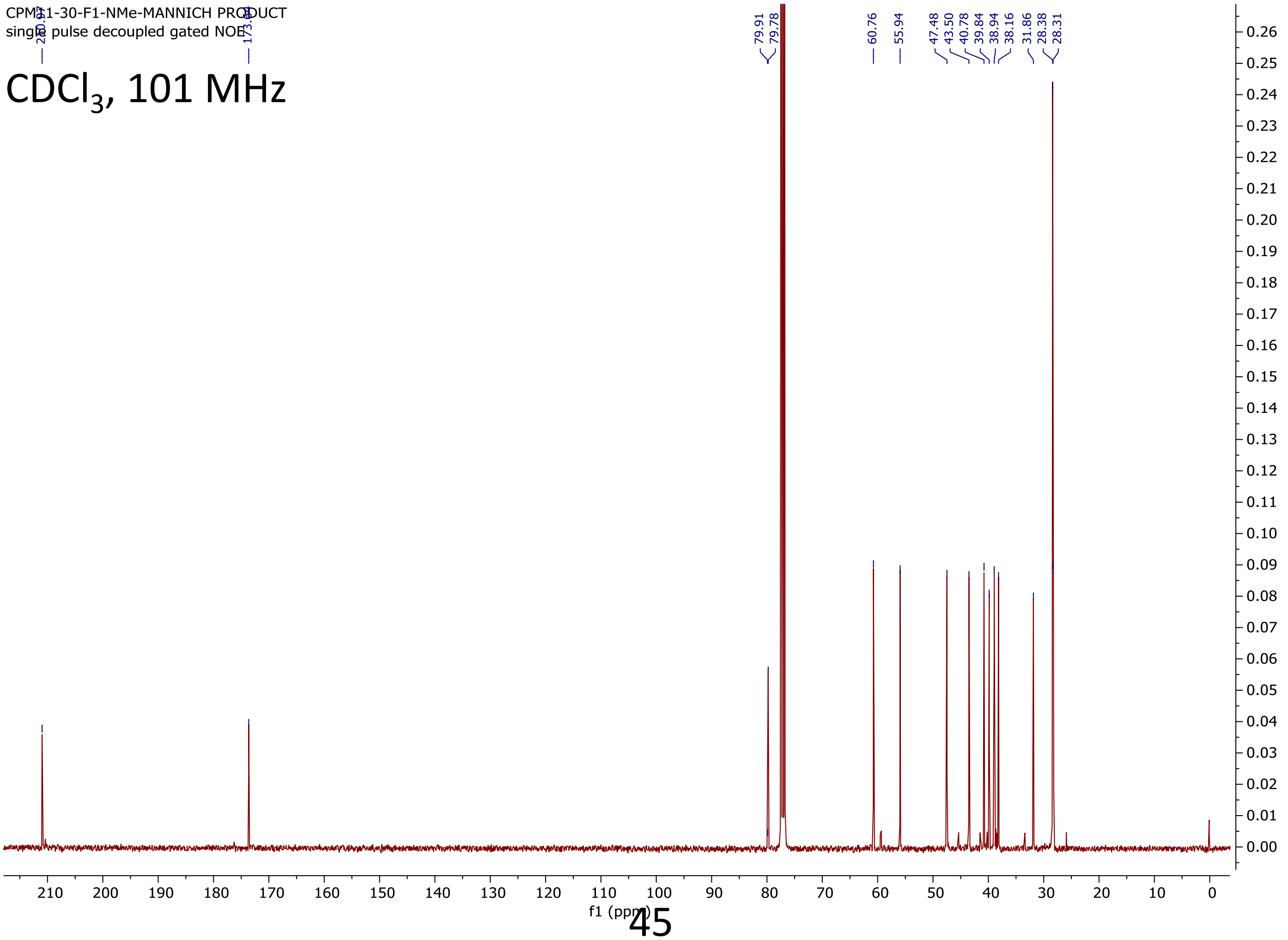
CPM11-30-F1-NMe-MANNICH PRODUCT
single pulse decoupled gated NOE
CDCl3, 101 MHz
173.64
79.91 79.78
60.76
55.94
47.48 43.50 40.78 39.84 38.94 38.16
31.86 28.38 28.31
f1 (ppm)

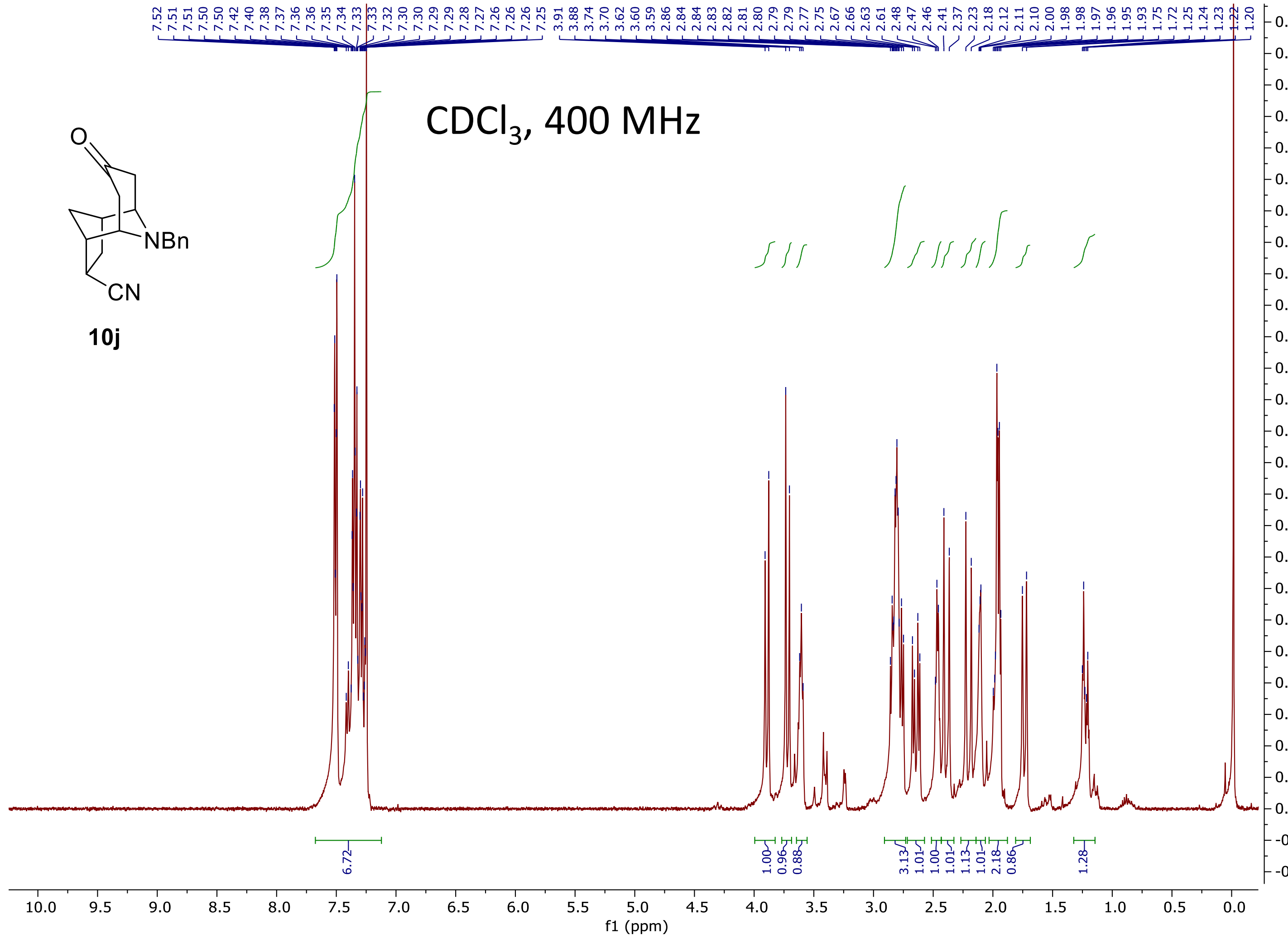
O
NBn
CN
10j
CDCl$_3$, 400 MHz
f1 (ppm)

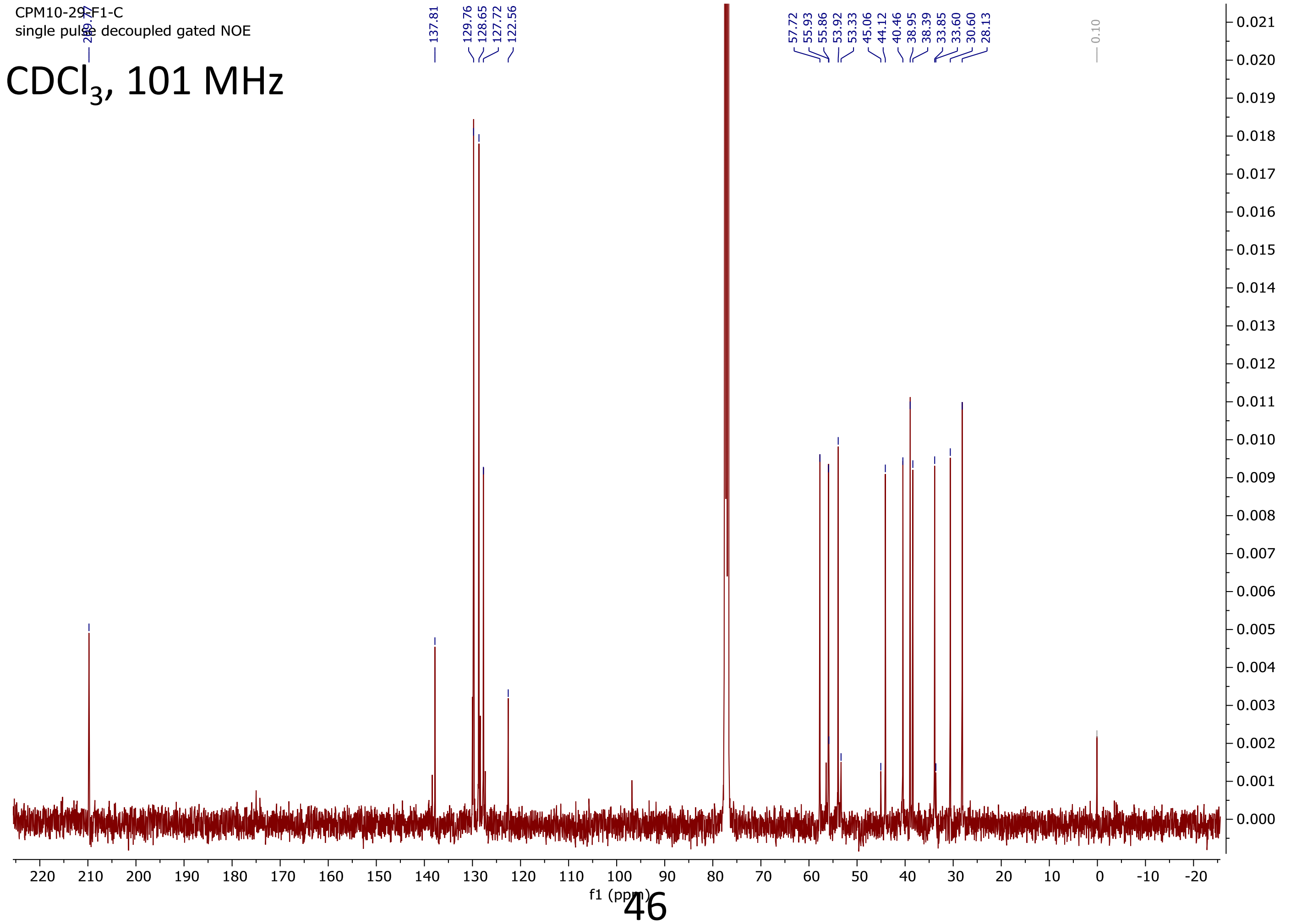
CPM10-29-F1-C
single pulse decoupled gated NOE
CDCl$_3$, 101 MHz
f1 (ppm)

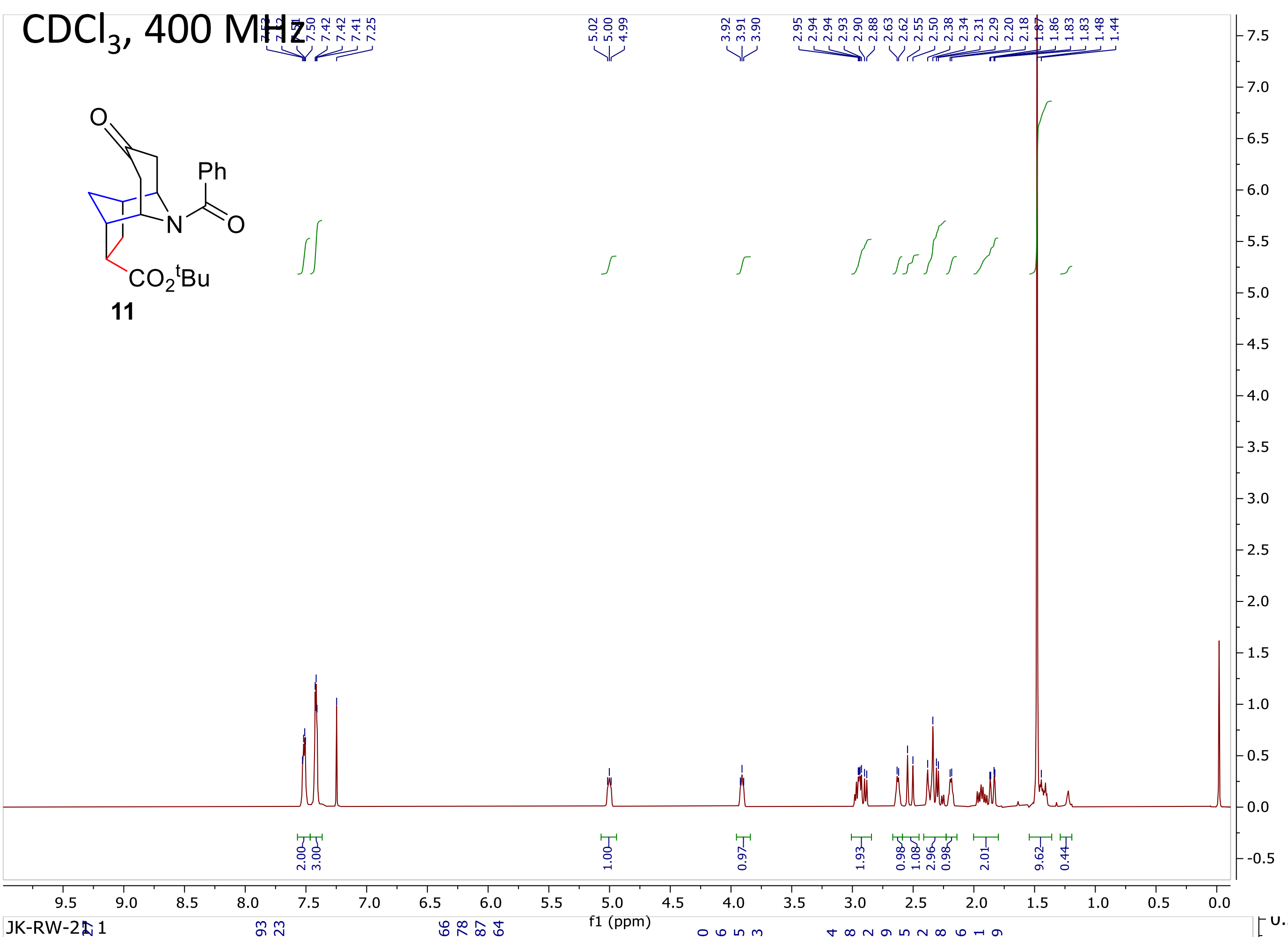
CDCl$_3$, 400 MHz
Ph
N
O
CO$_2$^{t}Bu
11
7.50 7.42 7.42 7.41 7.25
5.02 5.00 4.99
3.92 3.91 3.90
2.95 2.94 2.94 2.93 2.90 2.88 2.63 2.62 2.55 2.50 2.38 2.34 2.31 2.29 2.20 2.18 1.87 1.86 1.83 1.83 1.48 1.44
2.00 3.00 1.00 0.97 1.93 0.98 1.08 2.96 0.98 2.01 9.62 0.44
f1 (ppm)

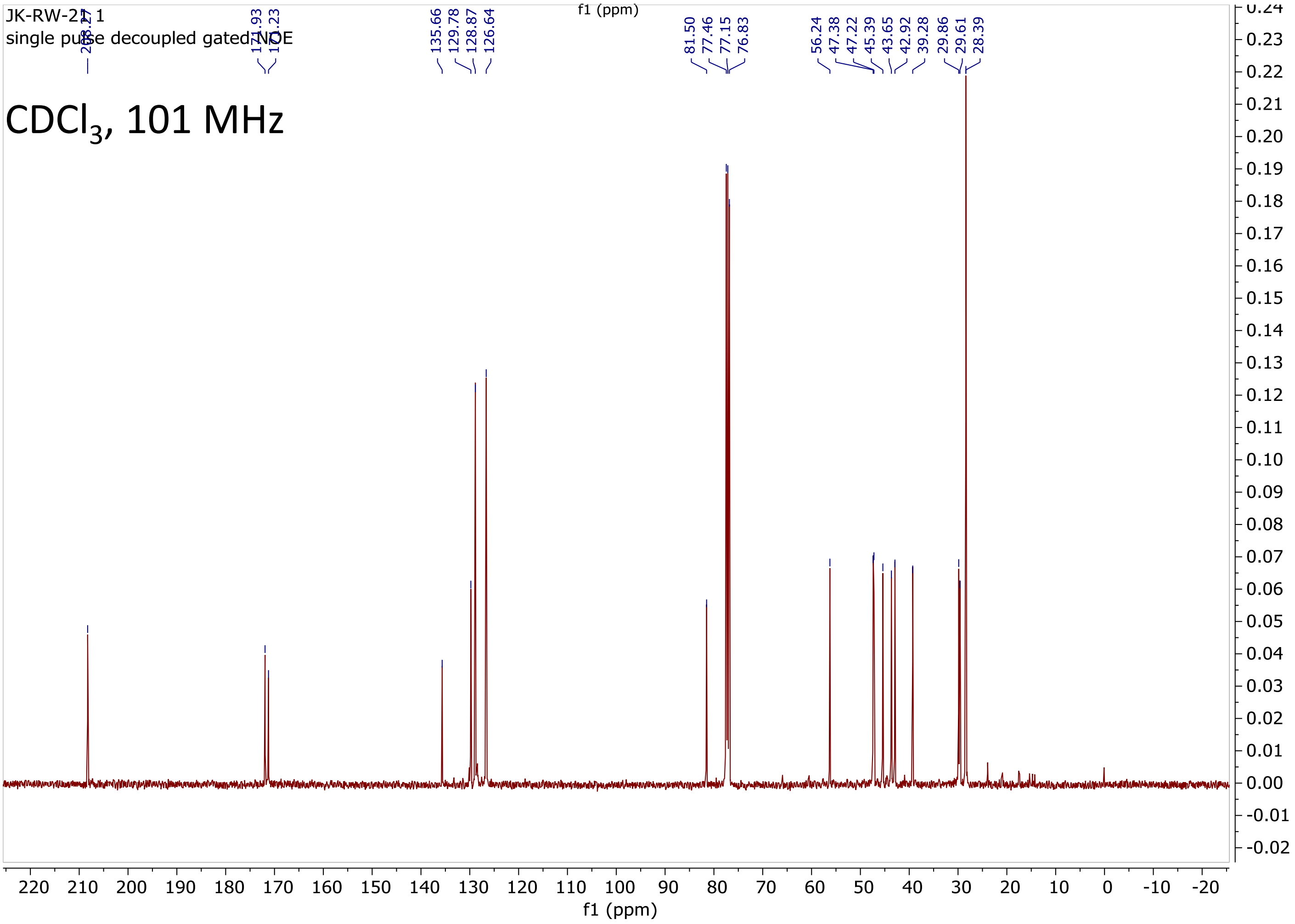
JK-RW-21 1
single pulse decoupled gated NOE
CDCl$_3$, 101 MHz
171.93 171.23
135.66 129.78 128.87 126.64
81.50 77.46 77.15 76.83
56.24 47.38 47.22 45.39 43.65 42.92 39.28 29.86 29.61 28.39
f1 (ppm)

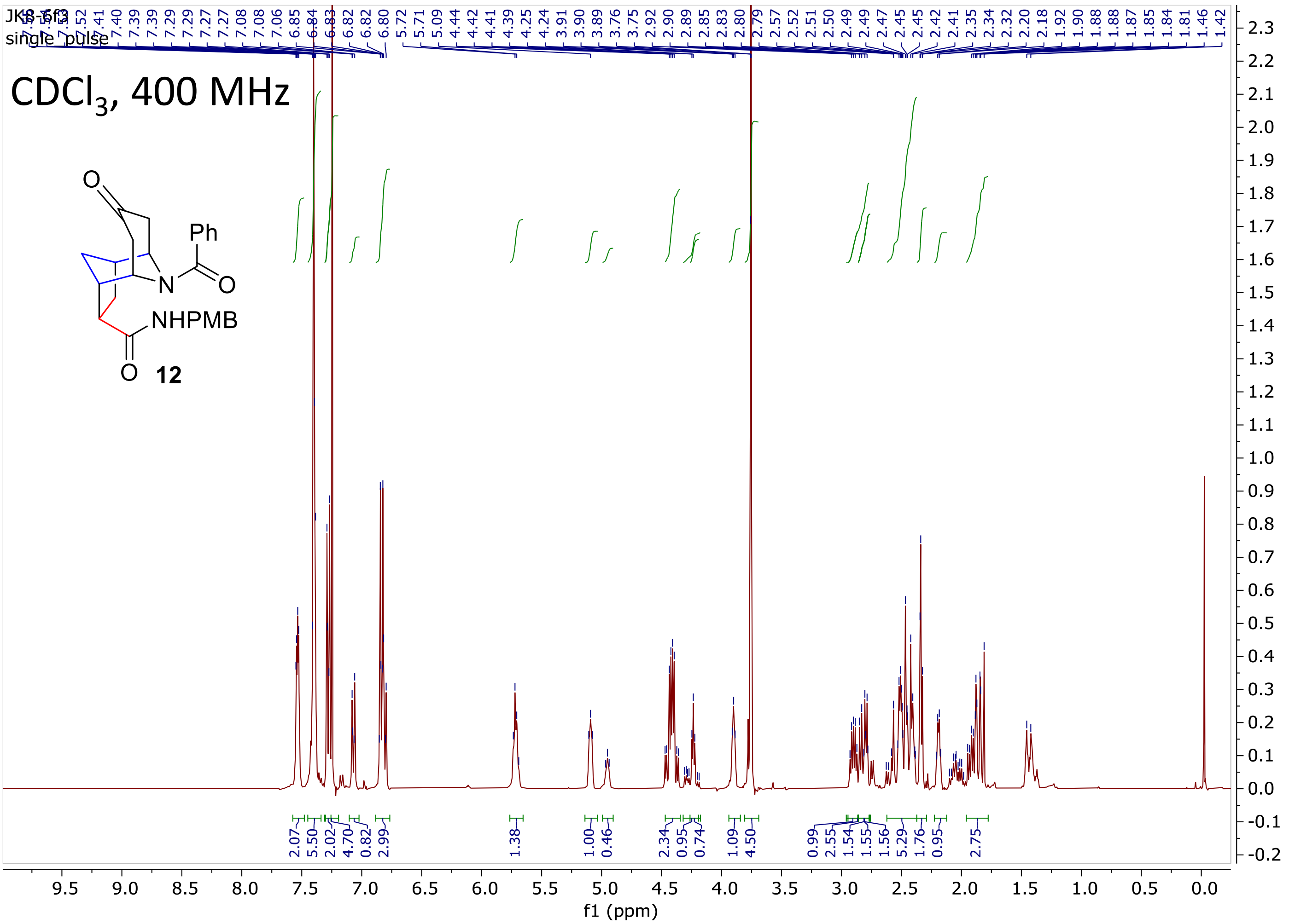
JK8-6f3
single pulse
CDCl3, 400 MHz
Ph
N
O
NHPMB
12
f1 (ppm)

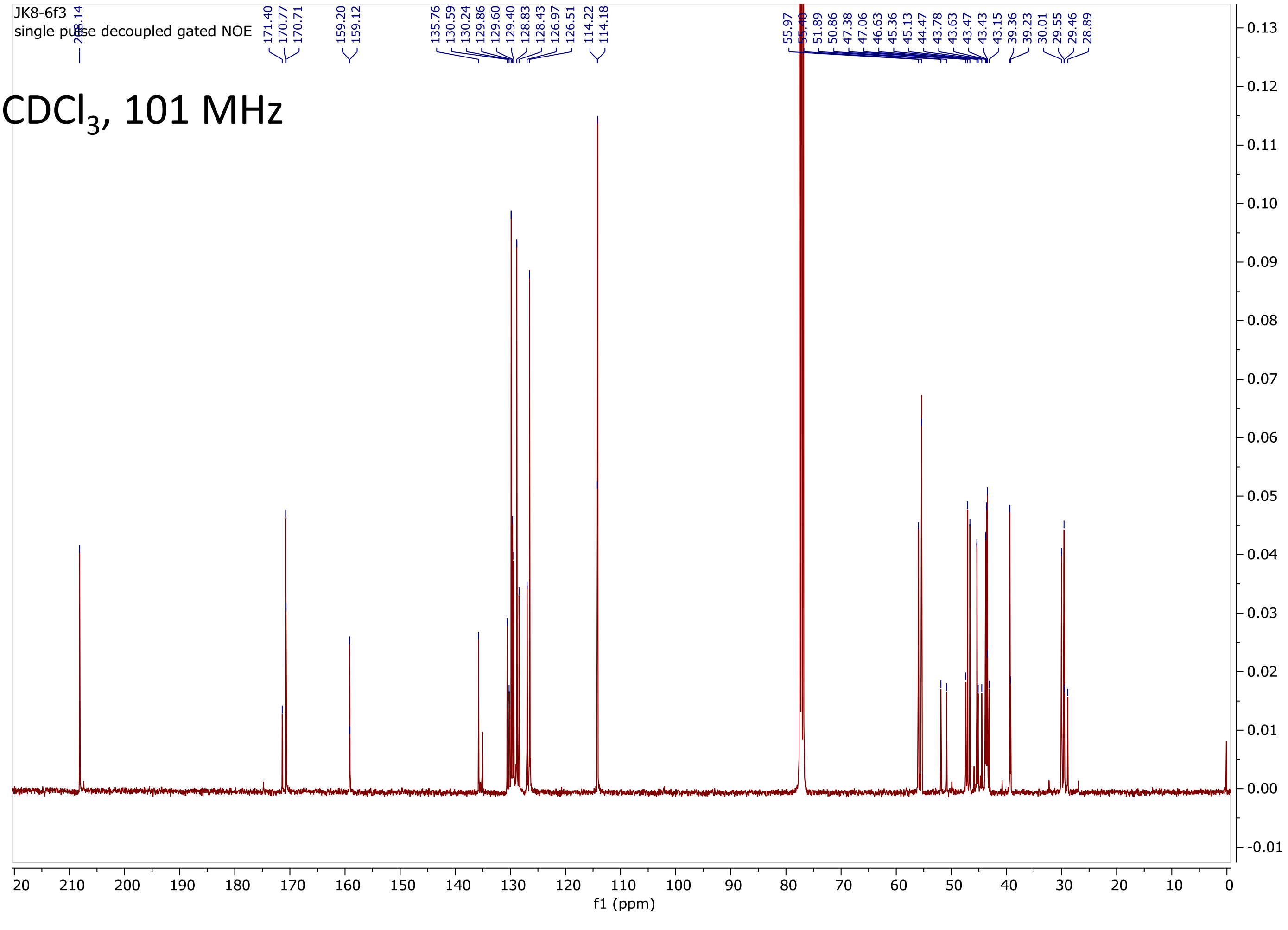
JK8-6f3
single pulse decoupled gated NOE
CDCl3, 101 MHz
f1 (ppm)

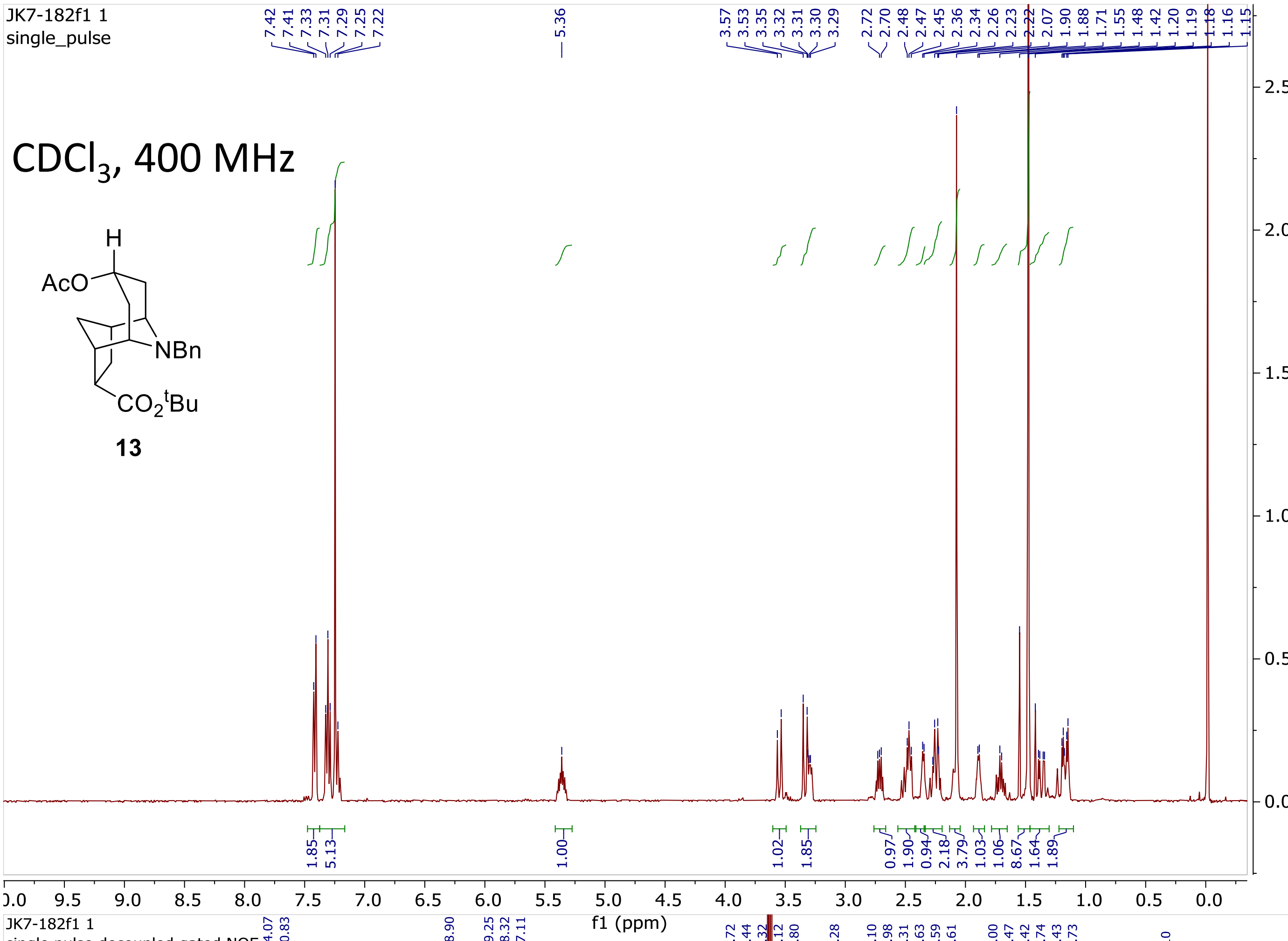
JK7-182f1 1
single_pulse
CDCl3, 400 MHz
H
AcO
NBn
CO2tBu
13
7.42 7.41 7.33 7.31 7.29 7.25 7.22
5.36
3.57 3.53 3.35 3.32 3.31 3.30 3.29
2.72 2.70 2.48 2.47 2.45 2.36 2.34 2.26 2.23 2.22 2.07 1.90 1.88 1.71 1.55 1.48 1.42 1.20 1.19 1.18 1.16 1.15
1.85 5.13 1.00 1.02 1.85 0.97 1.90 0.94 2.18 3.79 1.03 1.06 8.67 1.64 1.89
f1 (ppm)

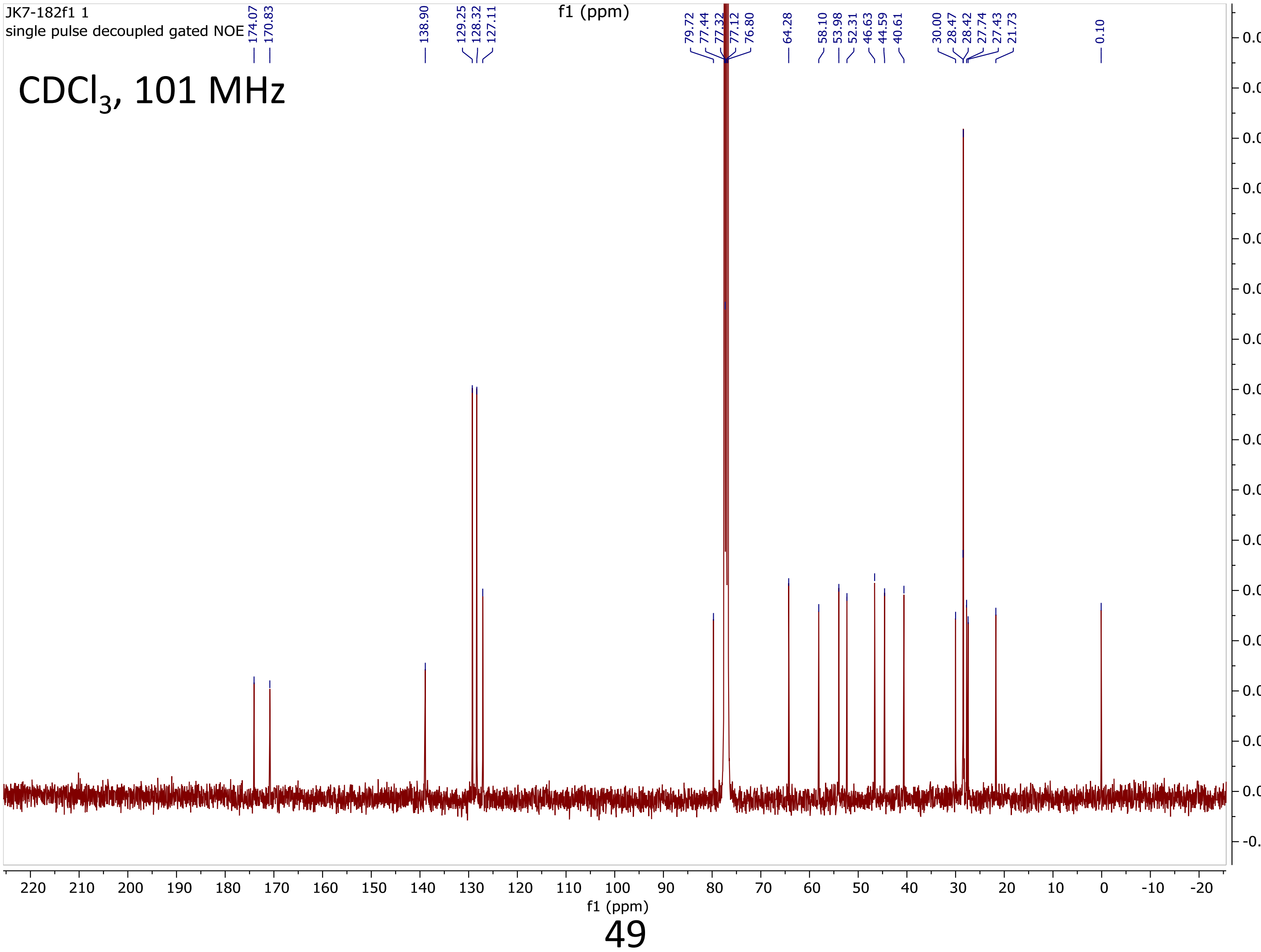
JK7-182f1 1
single pulse decoupled gated NOE
CDCl3, 101 MHz
174.07 170.83
138.90
129.25 128.32 127.11
79.72 77.44 77.32 77.12 76.80
64.28
58.10 53.98 52.31 46.63 44.59 40.61
30.00 28.47 28.42 27.74 27.43 21.73
0.10
f1 (ppm)

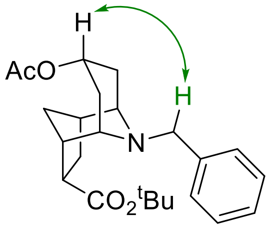

13

^1H-^1H NOESY spectrum. $CDCl_3$, 400 MHz

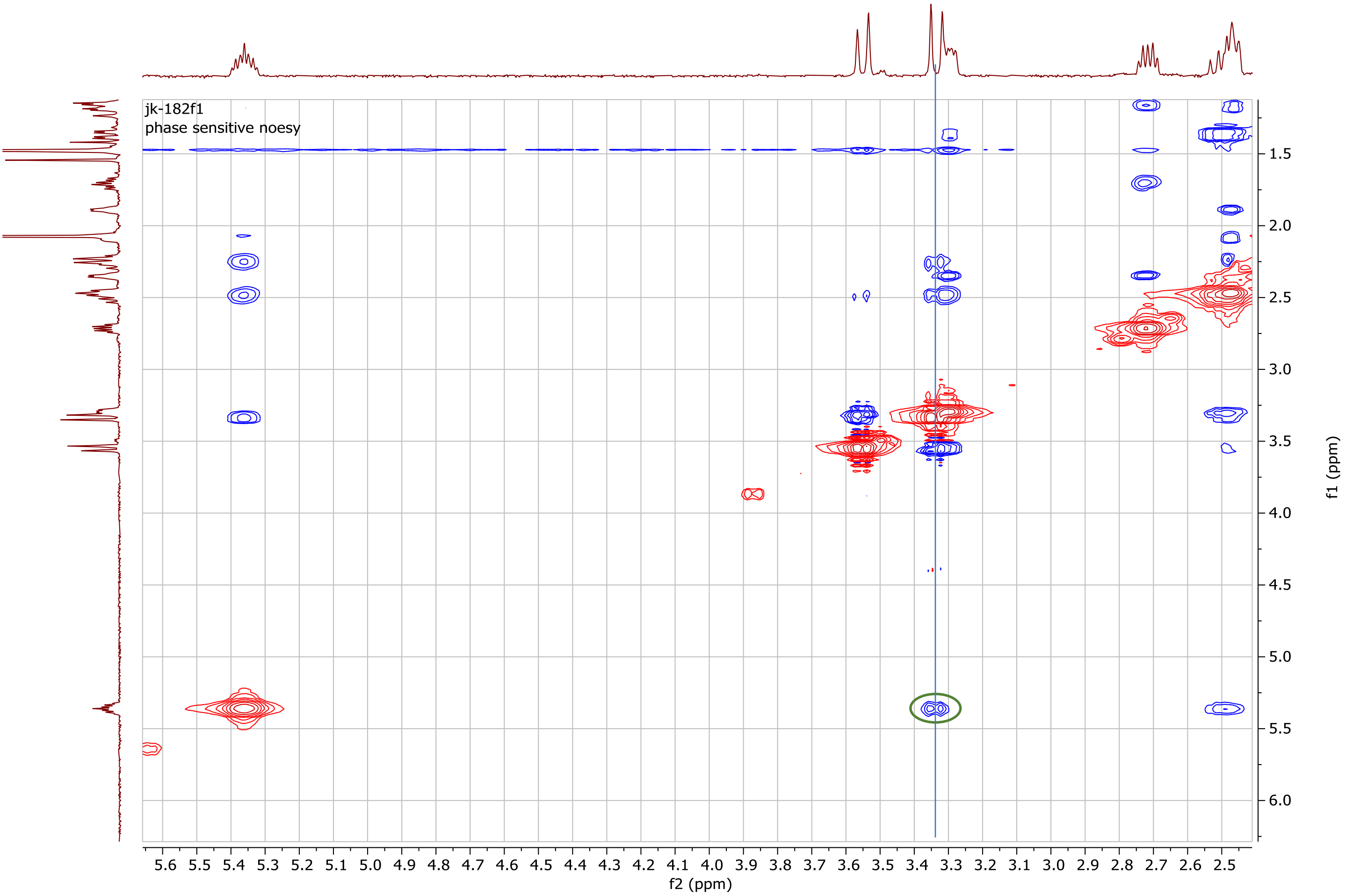

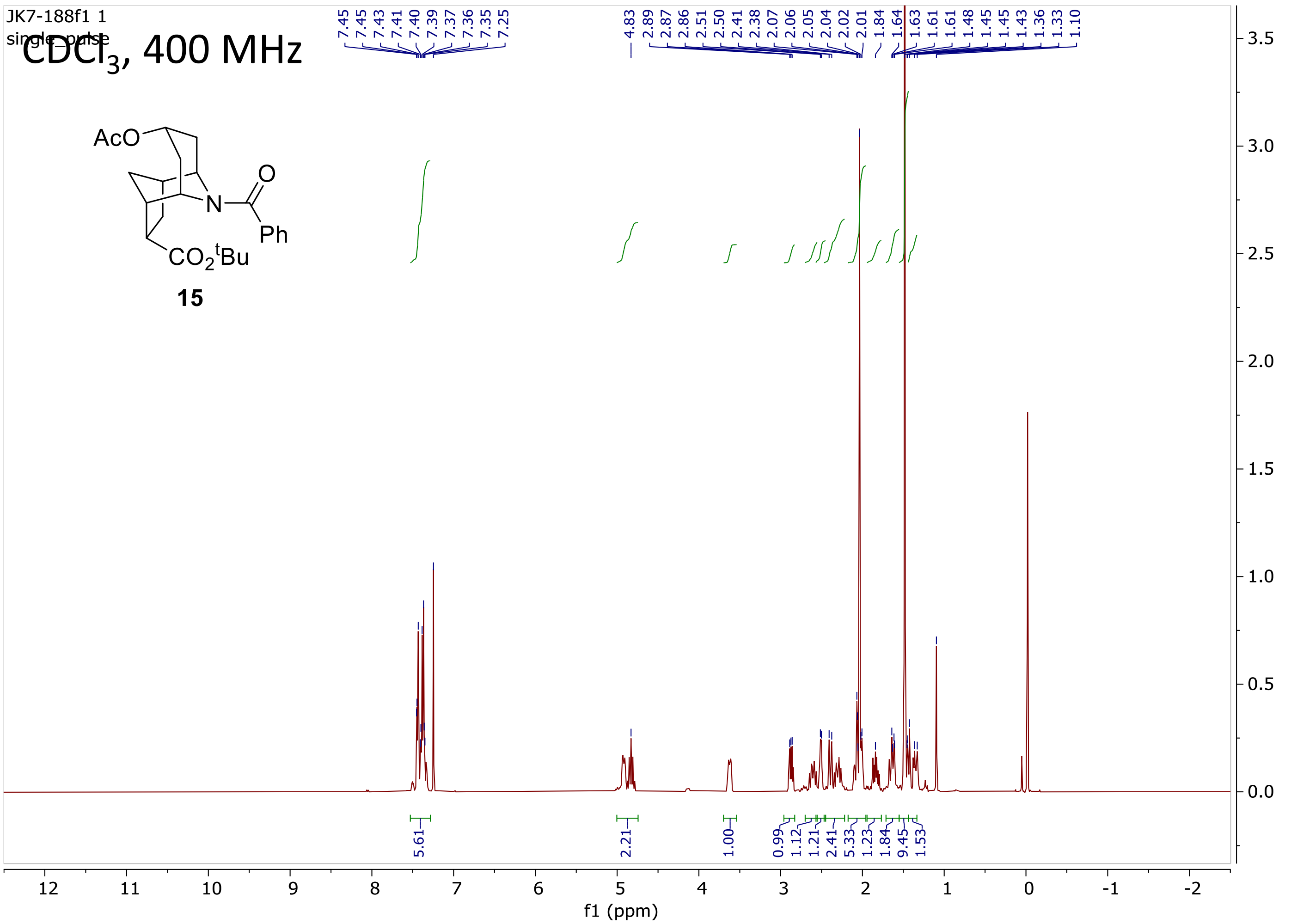
JK7-188f1 1
single_pulse
CDCl3, 400 MHz
AcO
N
O
Ph
CO2tBu
15
7.45 7.45 7.43 7.41 7.40 7.39 7.37 7.36 7.35 7.25
4.83 2.89 2.87 2.86 2.51 2.50 2.41 2.38 2.07 2.06 2.05 2.04 2.02 2.01 1.84 1.64 1.63 1.61 1.61 1.48 1.45 1.45 1.43 1.36 1.33 1.10
5.61 2.21 1.00 0.99 1.12 1.21 2.41 5.33 1.23 1.84 9.45 1.53
f1 (ppm)

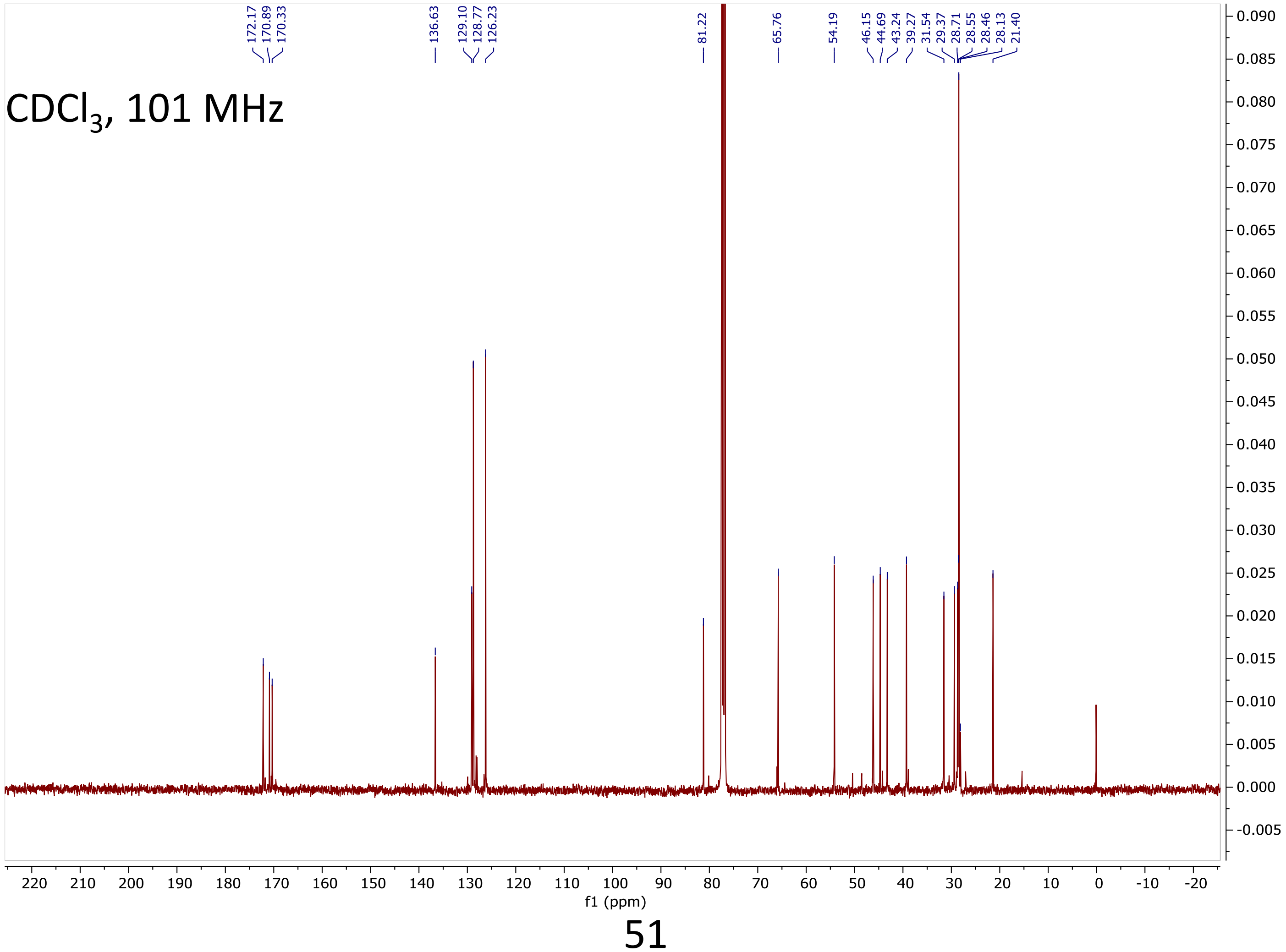
CDCl3, 101 MHz
172.17 170.89 170.33 136.63 129.10 128.77 126.23 81.22 65.76 54.19 46.15 44.69 43.24 39.27 31.54 29.37 28.71 28.55 28.46 28.13 21.40
f1 (ppm)

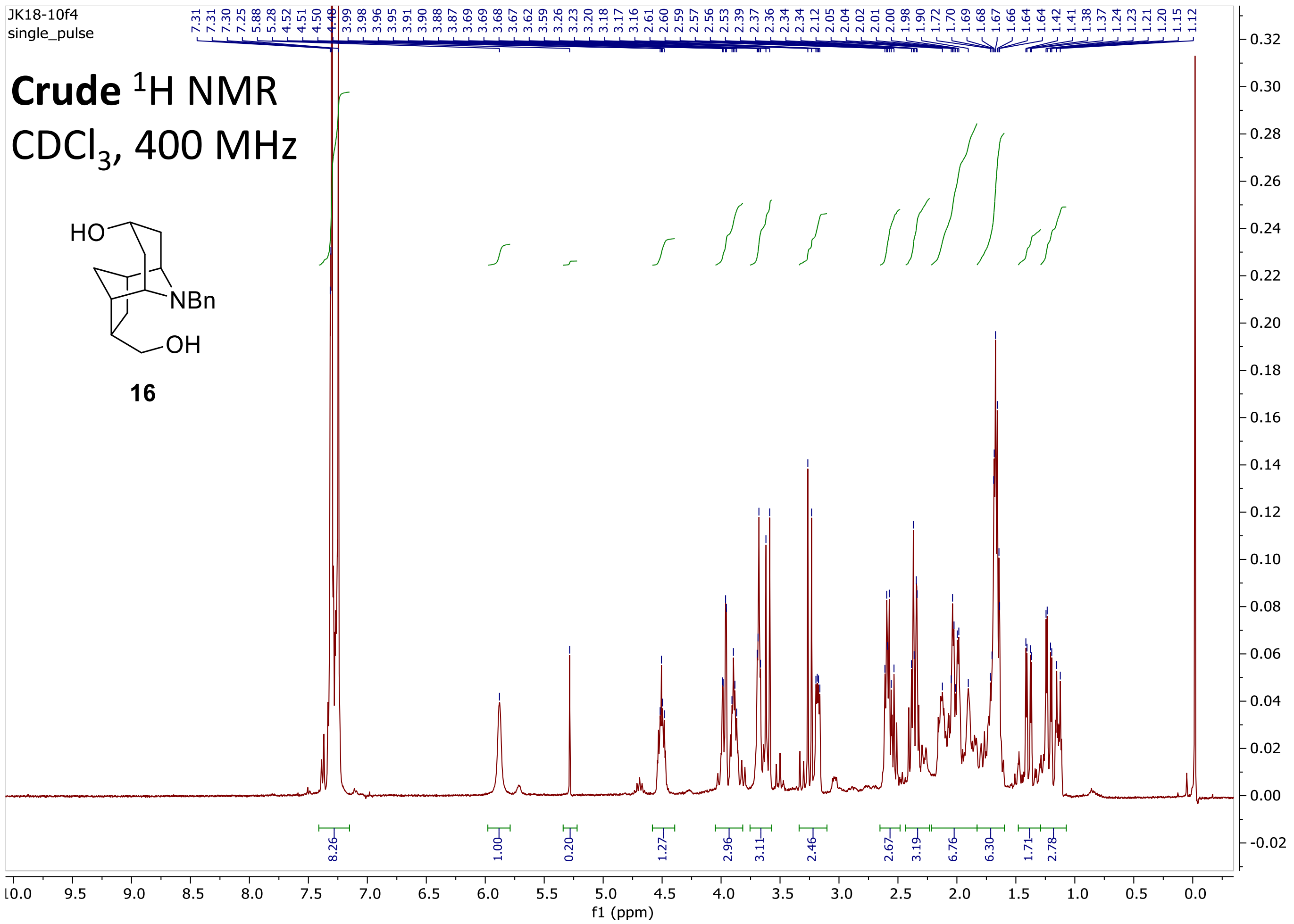
JK18-10f4
single_pulse
Crude 1H NMR
CDCl3, 400 MHz
HO
NBn
OH
16
7.31 7.31 7.30 7.25 5.88 5.28 4.52 4.51 4.50 4.49 3.99 3.98 3.96 3.95 3.91 3.90 3.88 3.87 3.69 3.69 3.68 3.67 3.62 3.59 3.26 3.23 3.20 3.18 3.17 3.16 2.61 2.60 2.59 2.57 2.56 2.53 2.39 2.37 2.36 2.34 2.34 2.12 2.05 2.04 2.02 2.01 2.00 1.98 1.90 1.72 1.70 1.69 1.68 1.67 1.66 1.64 1.64 1.42 1.41 1.38 1.37 1.24 1.23 1.21 1.20 1.15 1.12
8.26 1.00 0.20 1.27 2.96 3.11 2.46 2.67 3.19 6.76 6.30 1.71 2.78
f1 (ppm)

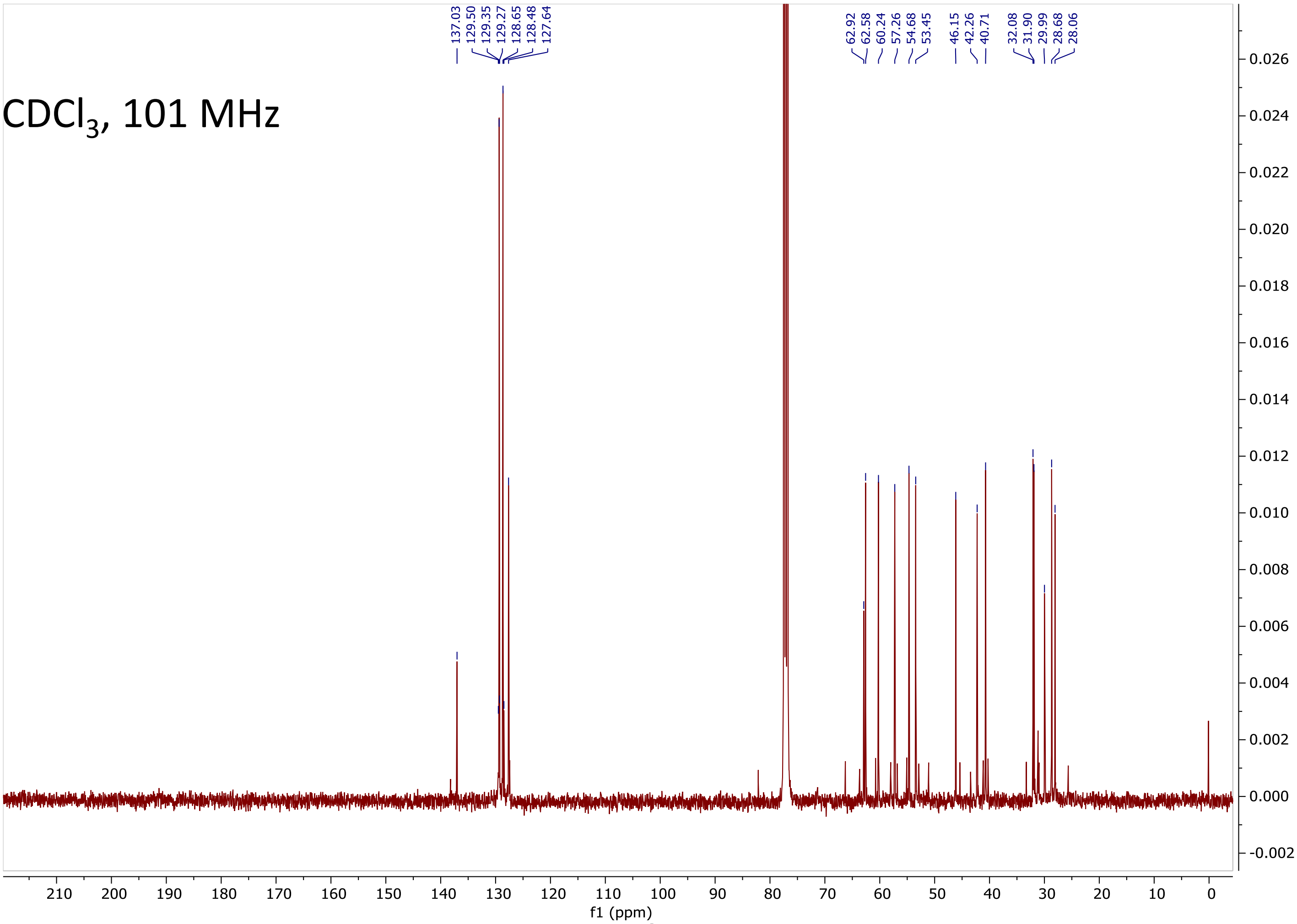
CDCl3, 101 MHz
137.03 129.50 129.35 129.27 128.65 128.48 127.64
62.92 62.58 60.24 57.26 54.68 53.45 46.15 42.26 40.71 32.08 31.90 29.99 28.68 28.06
f1 (ppm)

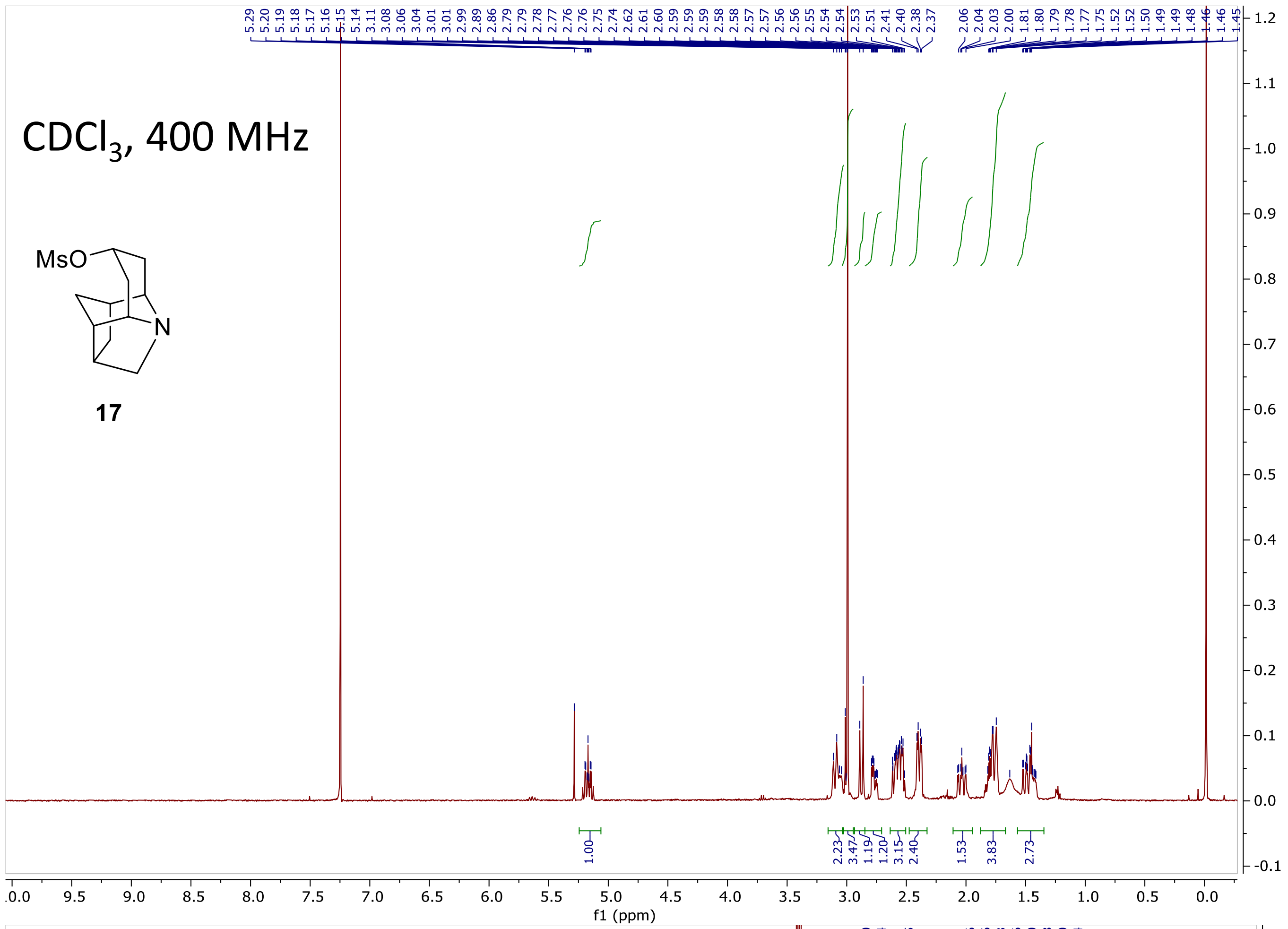
CDCl$_3$, 400 MHz
MsO
N
17
5.29 5.20 5.19 5.18 5.17 5.16 5.15 5.14 3.11 3.08 3.06 3.04 3.01 3.01 2.99 2.89 2.86 2.79 2.79 2.78 2.77 2.76 2.76 2.75 2.74 2.62 2.61 2.60 2.59 2.59 2.59 2.58 2.58 2.57 2.57 2.56 2.56 2.55 2.54 2.54 2.53 2.51 2.41 2.40 2.38 2.37 2.06 2.04 2.03 2.00 1.81 1.80 1.79 1.78 1.77 1.75 1.52 1.52 1.50 1.49 1.48 1.46 1.46 1.45
1.00 2.23 3.47 1.19 1.20 3.15 2.40 1.53 3.83 2.73
f1 (ppm)

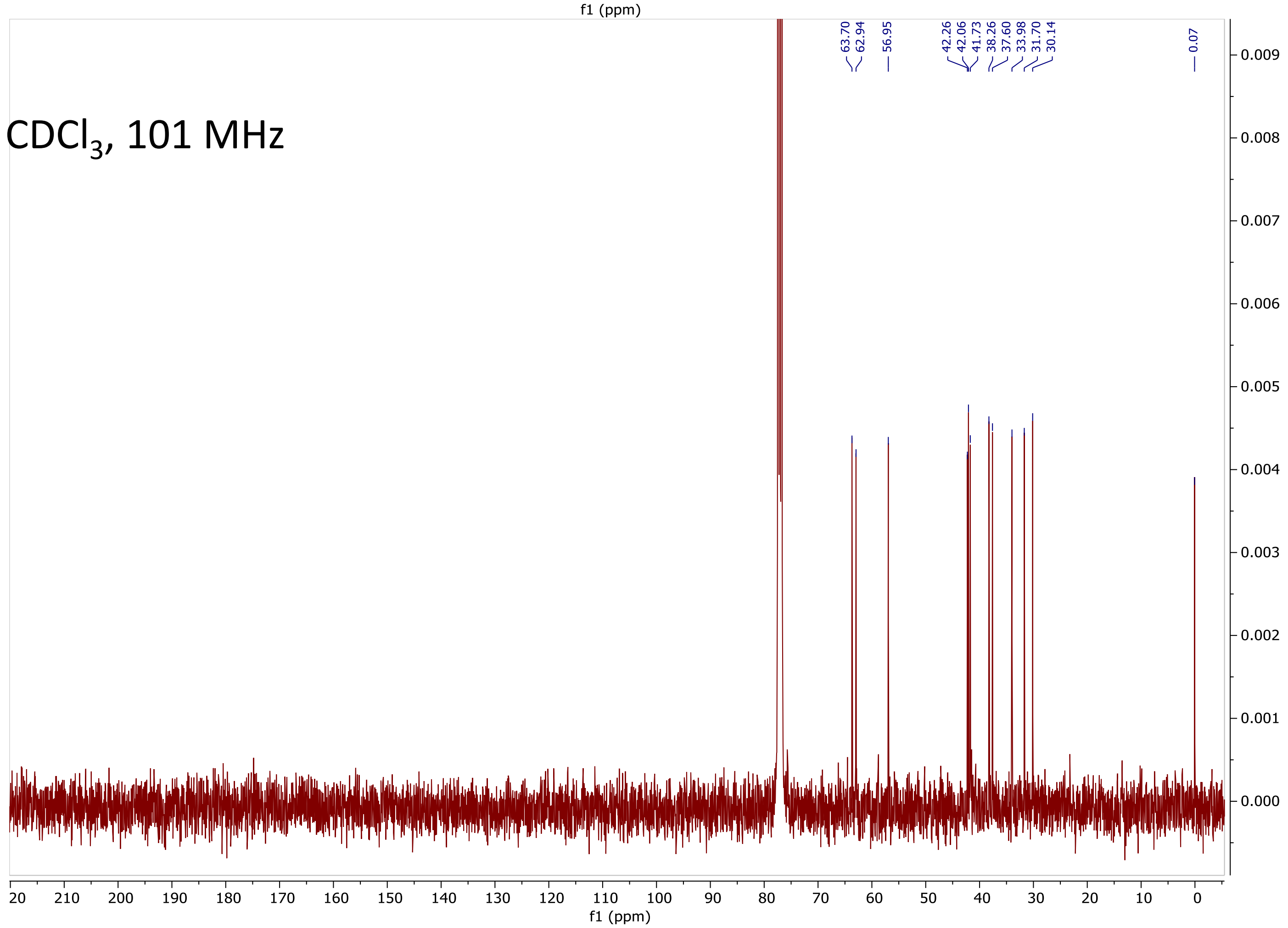
CDCl$_3$, 101 MHz
63.70 62.94 56.95 42.26 42.06 41.73 38.26 37.60 33.98 31.70 30.14 0.07
f1 (ppm)

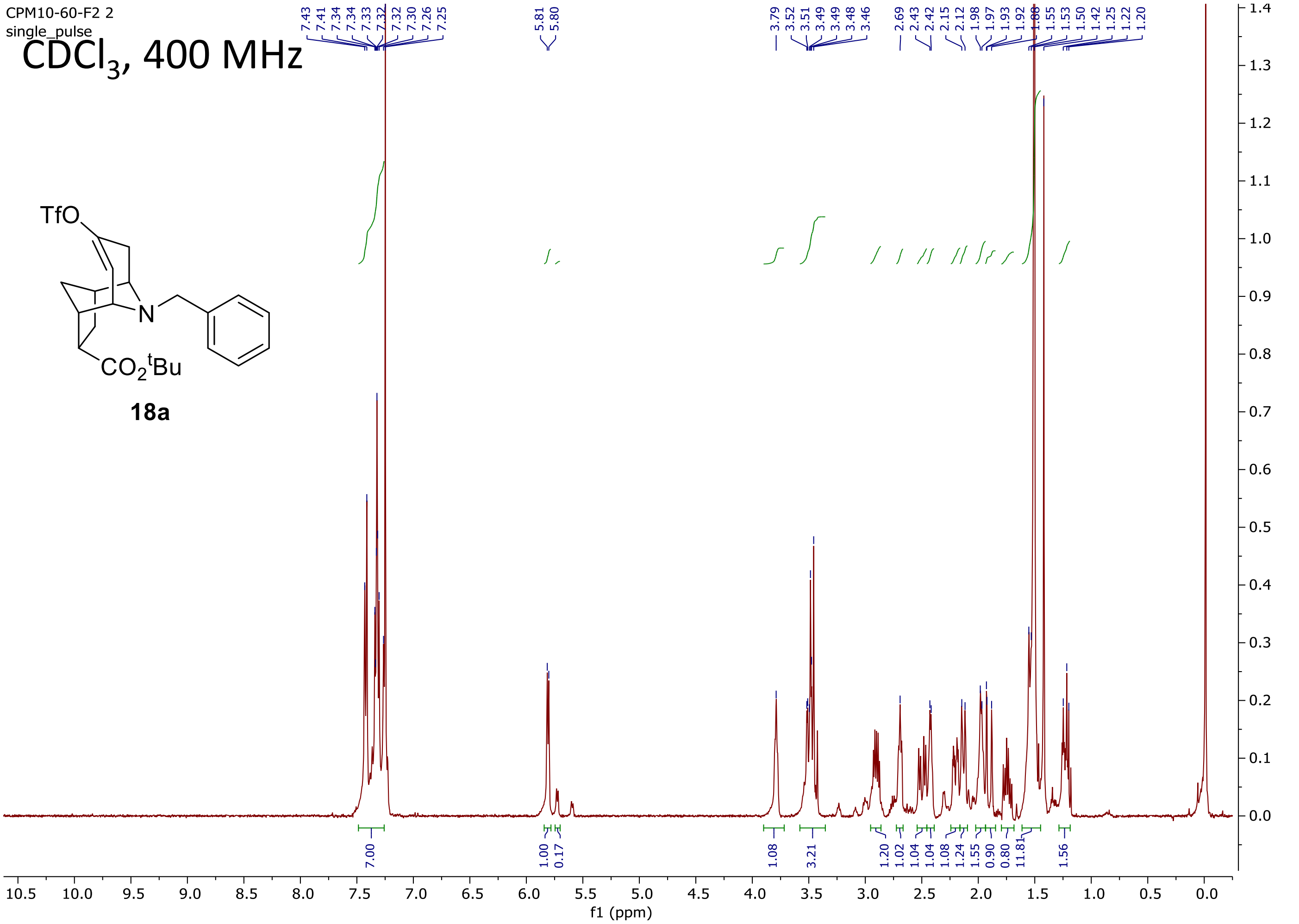

CPM10-60-F2 2
single_pulse
CDCl3, 400 MHz
TfO
N
CO2tBu
18a
7.43 7.41 7.34 7.34 7.33 7.32 7.32 7.30 7.26 7.25
5.81 5.80
3.79 3.52 3.51 3.49 3.49 3.48 3.46
2.69 2.43 2.42 2.15 2.12 1.98 1.97 1.93 1.92 1.88 1.55 1.53 1.50 1.42 1.25 1.22 1.20
7.00 1.00 0.17 1.08 3.21 1.20 1.02 1.04 1.04 1.08 1.24 1.55 0.90 0.80 11.81 1.56
f1 (ppm)

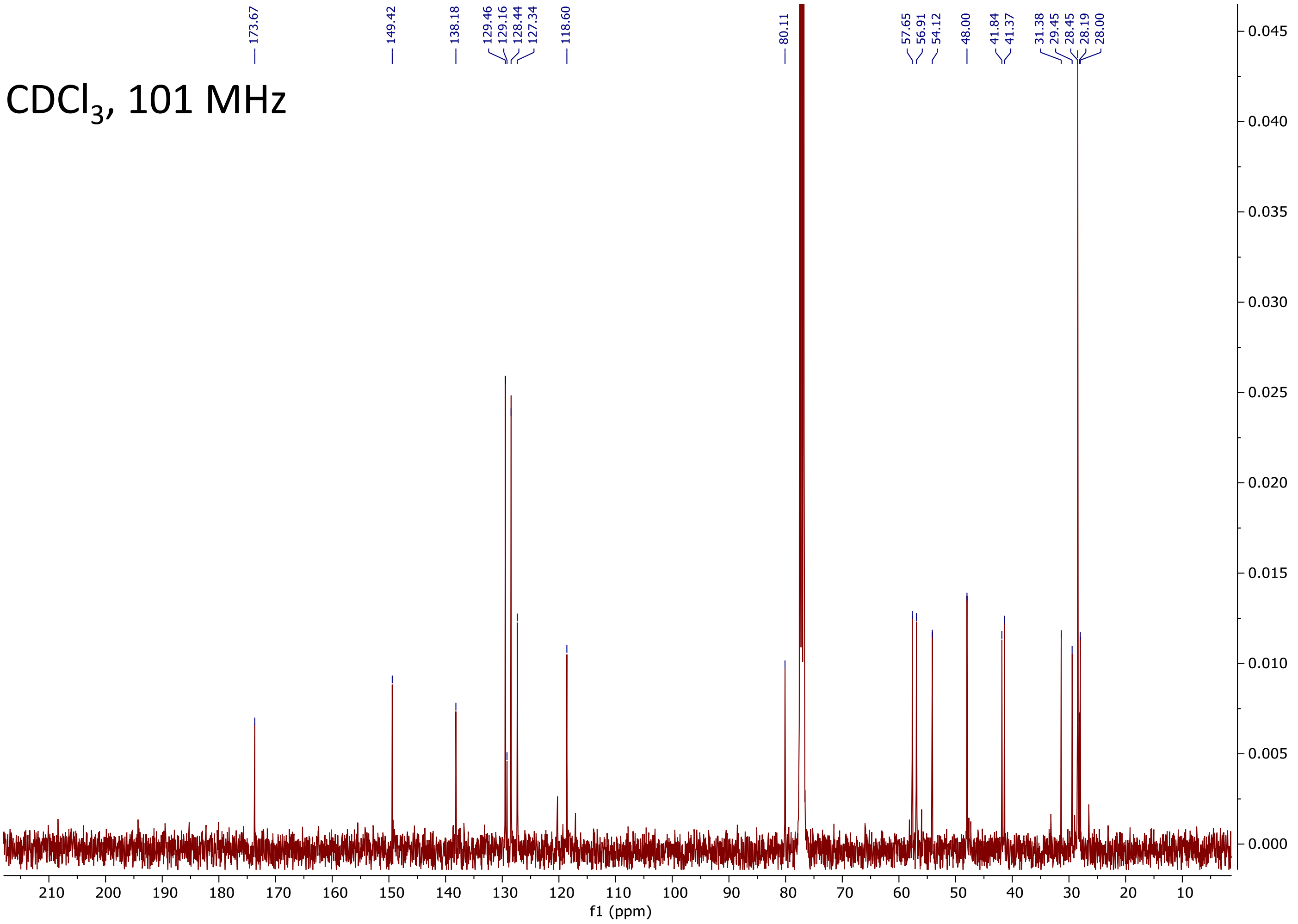

CDCl3, 101 MHz
173.67 149.42 138.18 129.46 129.16 128.44 127.34 118.60 80.11
57.65 56.91 54.12 48.00 41.84 41.37 31.38 29.45 28.45 28.19 28.00
f1 (ppm)

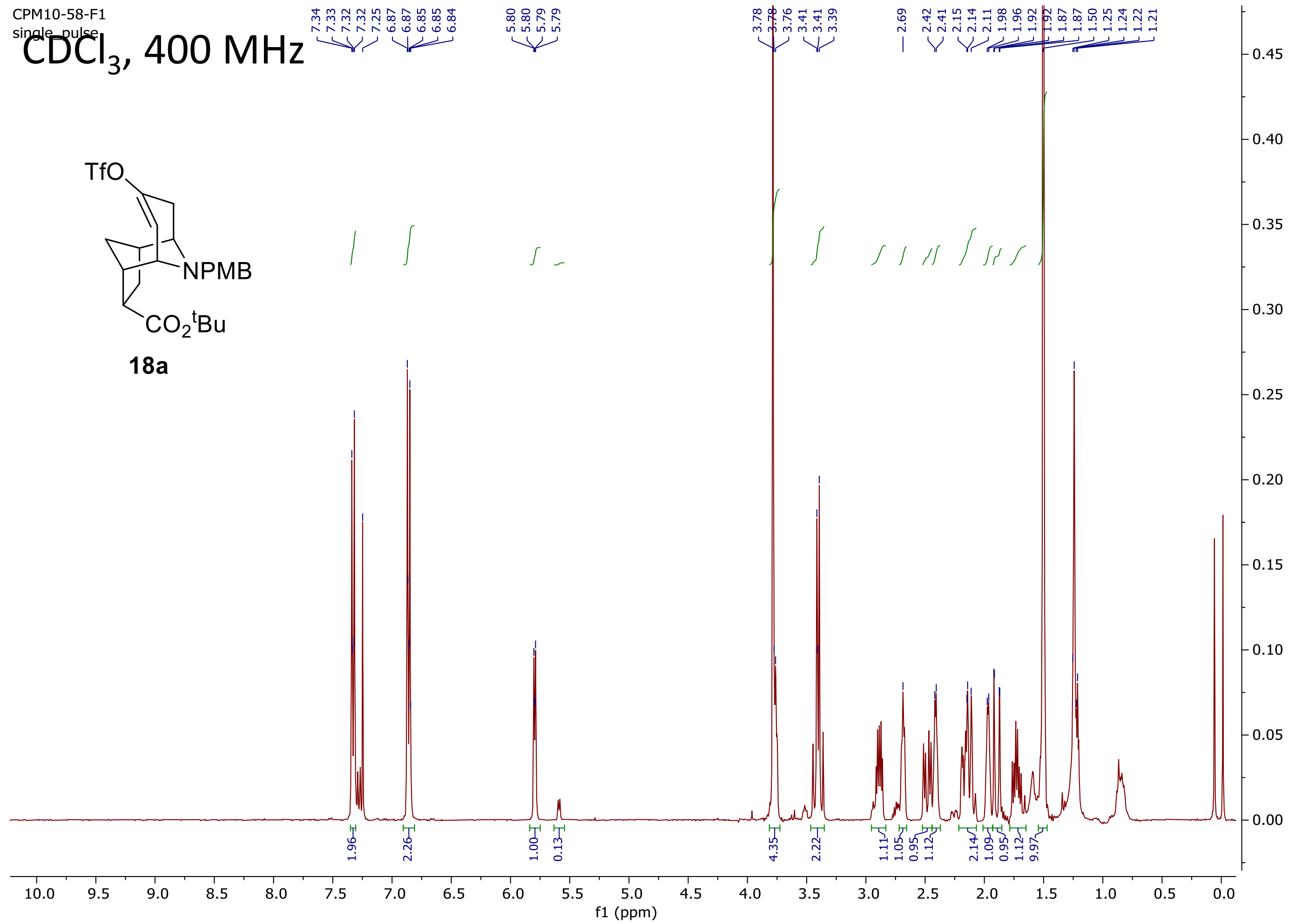
CPM10-58-F1
single_pulse
CDCl3, 400 MHz
7.34 7.33 7.32 7.32 7.25 6.87 6.87 6.85 6.85 6.84
5.80 5.80 5.79 5.79
3.78 3.76 3.76 3.41 3.41 3.39
2.69 2.42 2.41 2.15 2.14 2.11 1.98 1.96 1.92 1.92 1.87 1.87 1.50 1.25 1.24 1.22 1.21
TfO
NPMB
CO2tBu
18a
1.96 2.26 1.00 0.13 4.35 2.22 1.11 1.05 0.95 1.12 2.14 1.09 0.95 1.12 9.97
f1 (ppm)

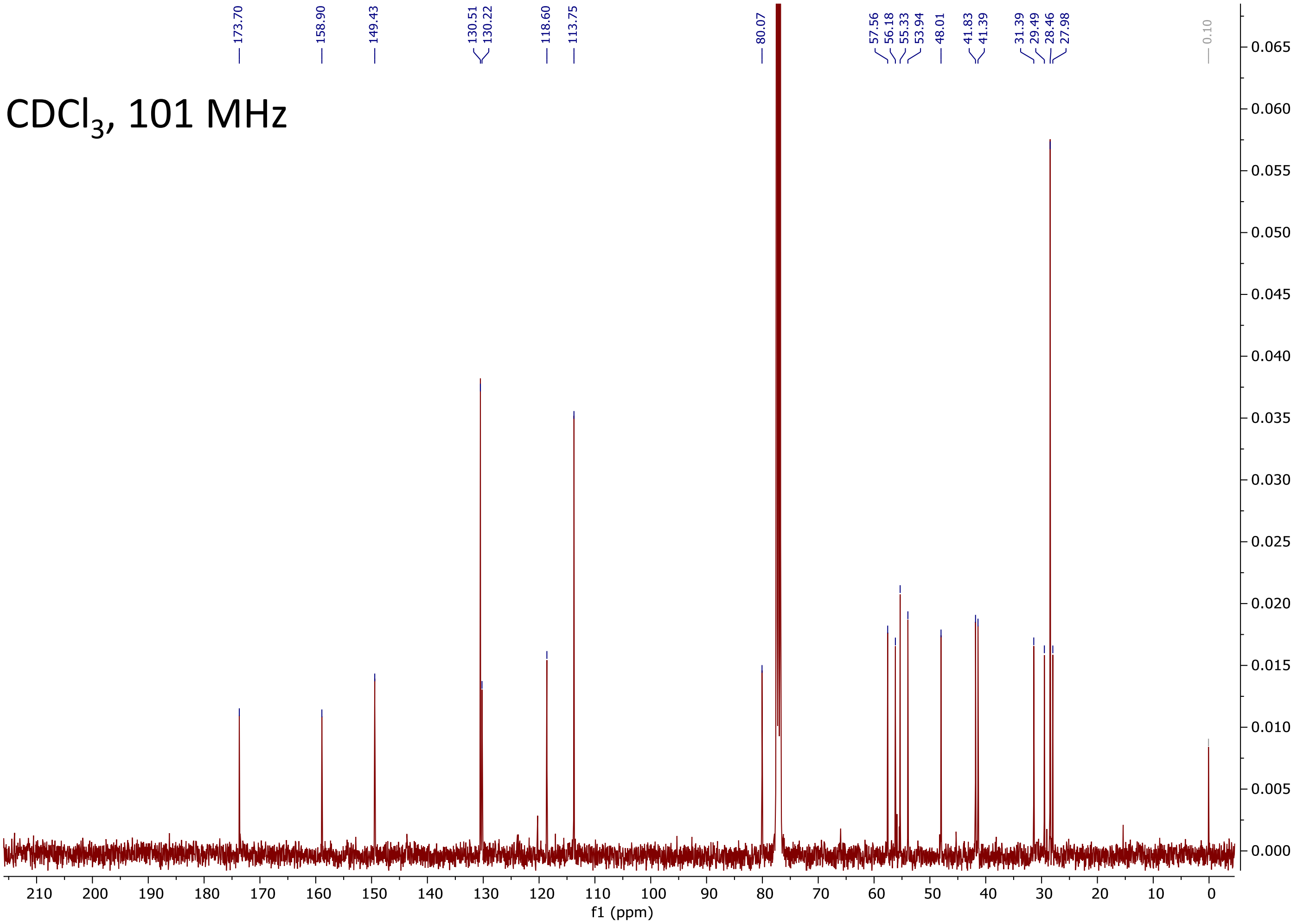
CDCl3, 101 MHz
173.70
158.90
149.43
130.51 130.22
118.60
113.75
80.07
57.56 56.18 55.33 53.94
48.01
41.83 41.39
31.39 29.49 28.46 27.98
0.10
f1 (ppm)

CDCl$_3$, 400 MHz

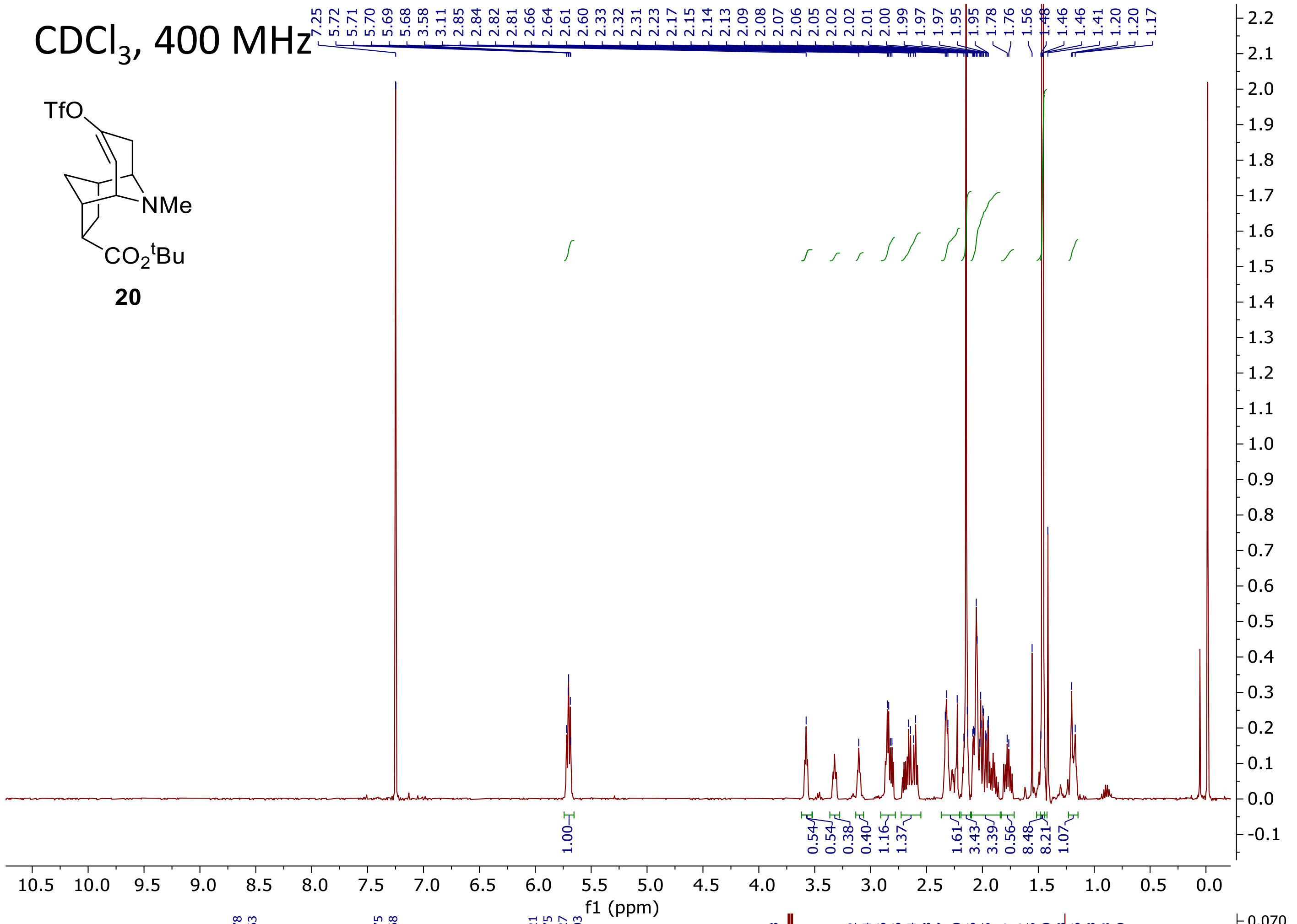

CDCl$_3$, 101 MHz

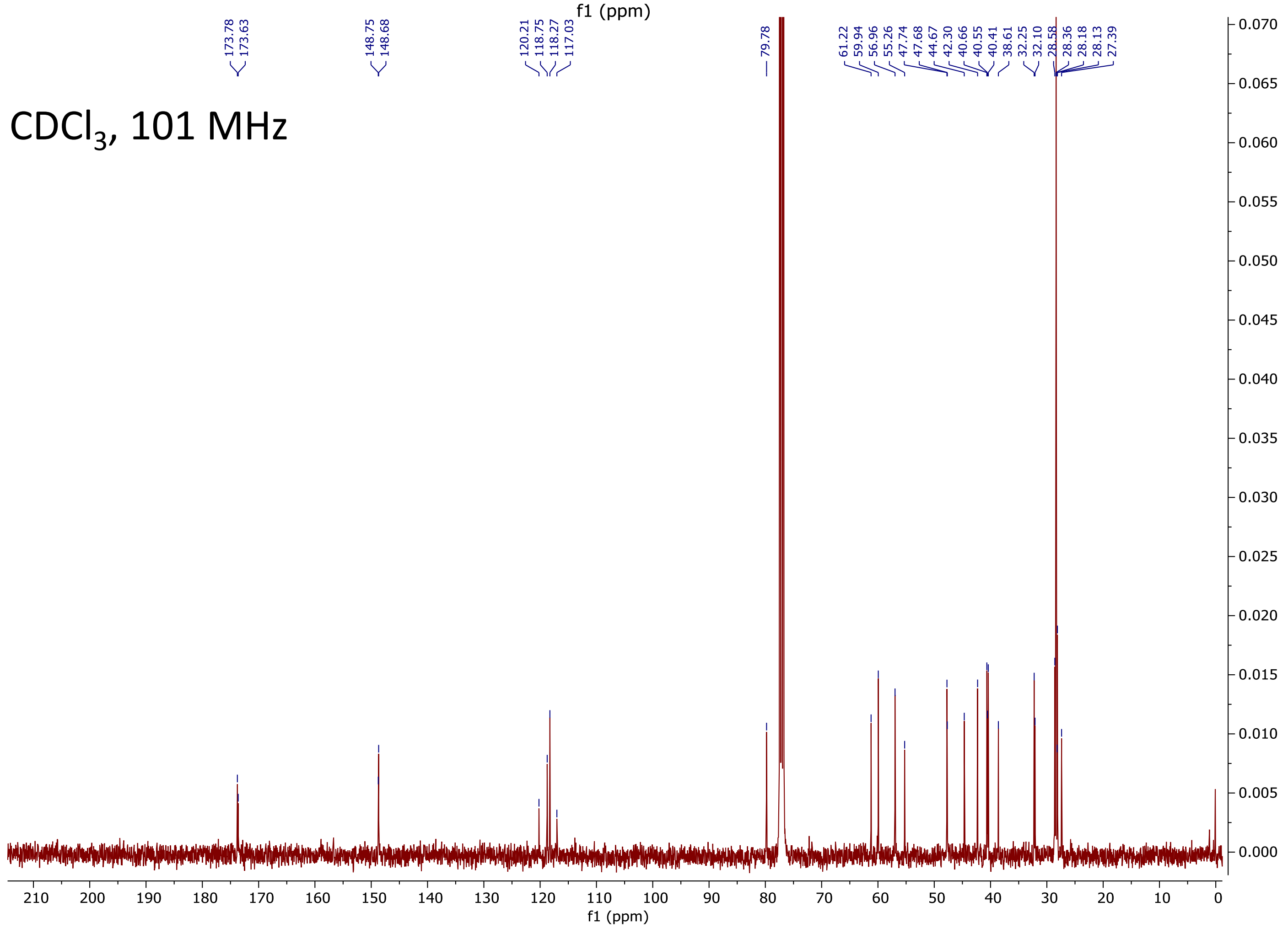

CDCl$_3$, 400 MHz

19a

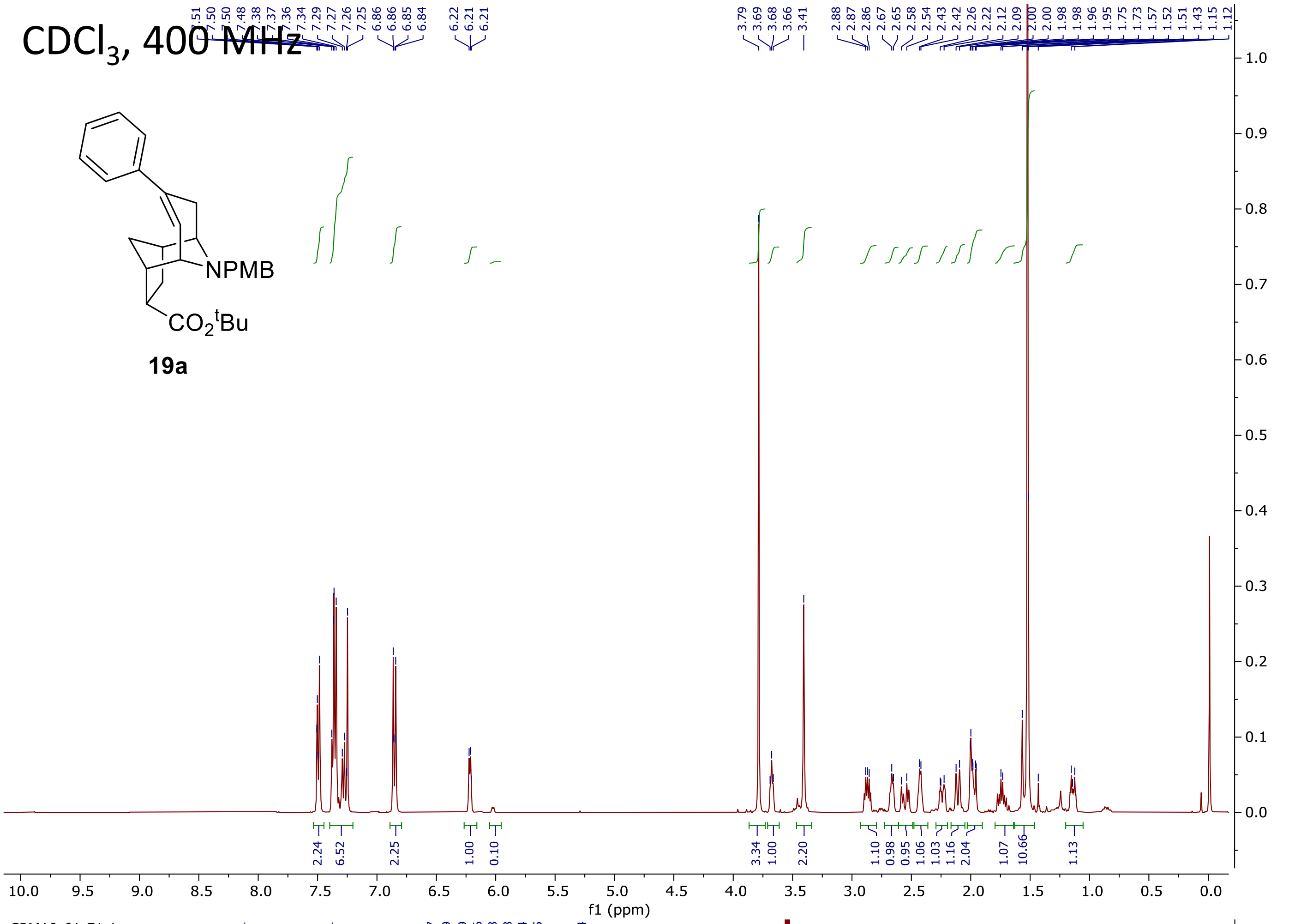

CPM10-61-F1 1
single pulse decoupled gated NOE

CDCl$_3$, 101 MHz

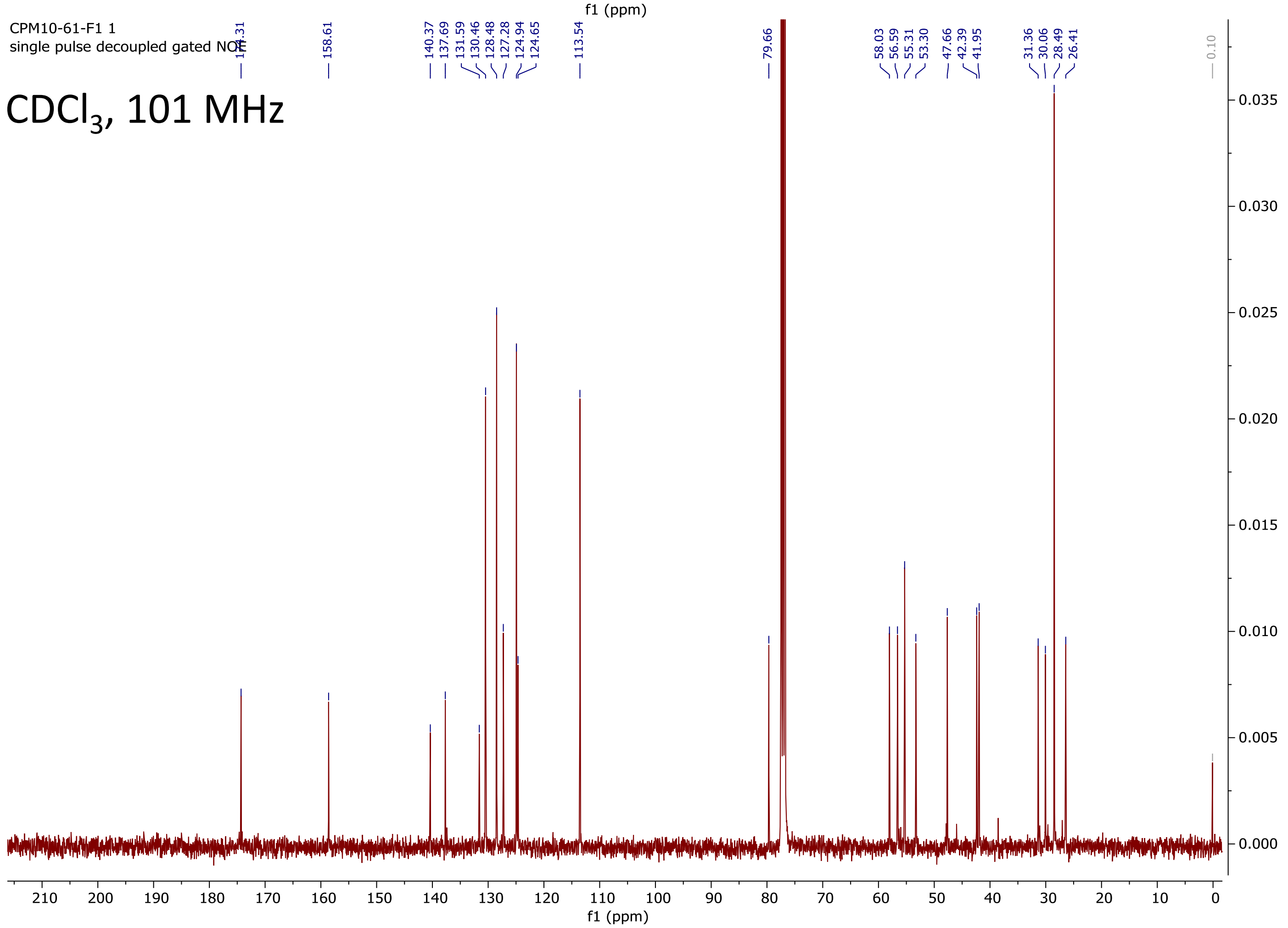

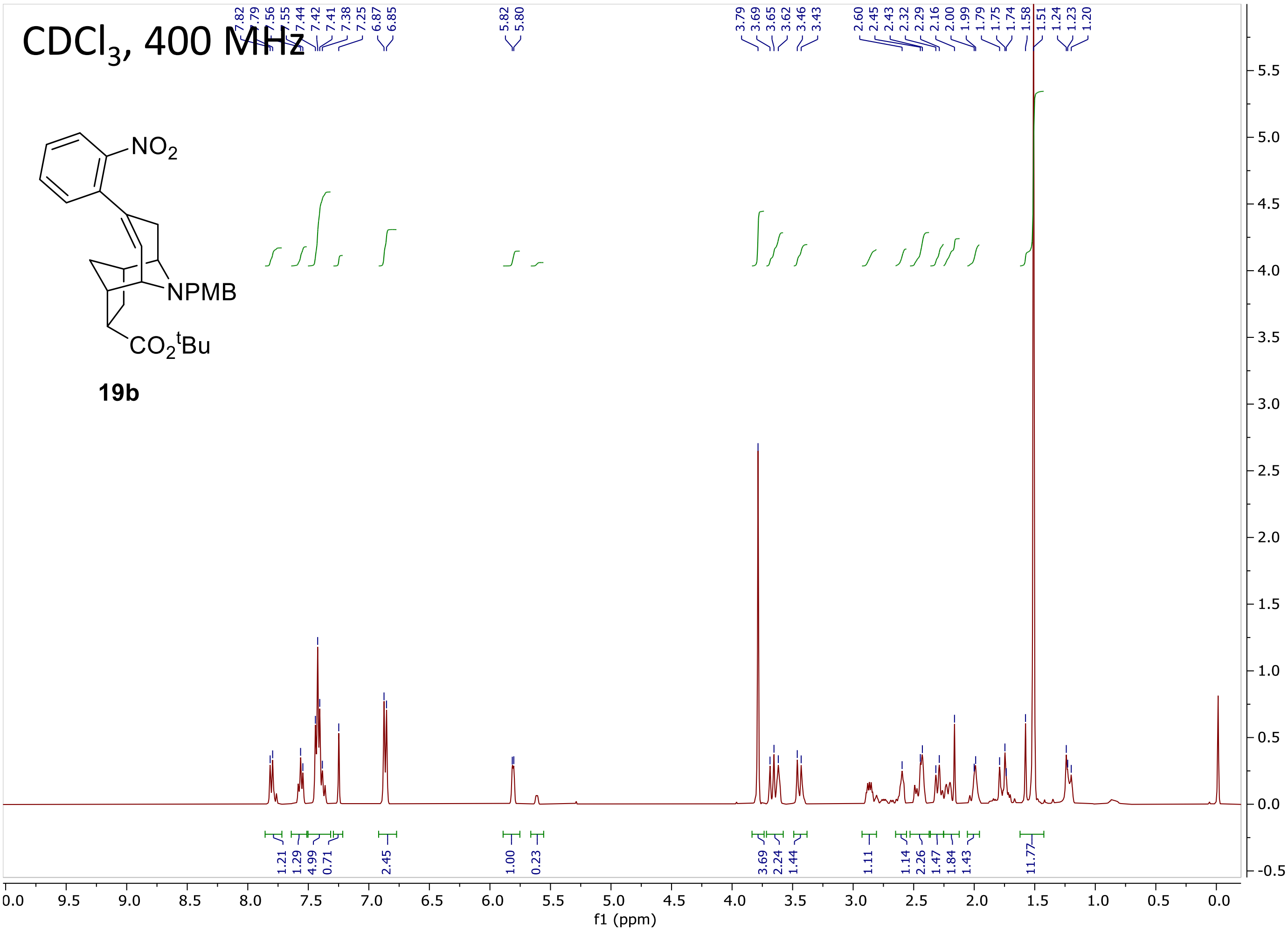
CDCl$_3$, 400 MHz
NO$_2$
NPMB
CO$_2$^{t}Bu
19b
f1 (ppm)

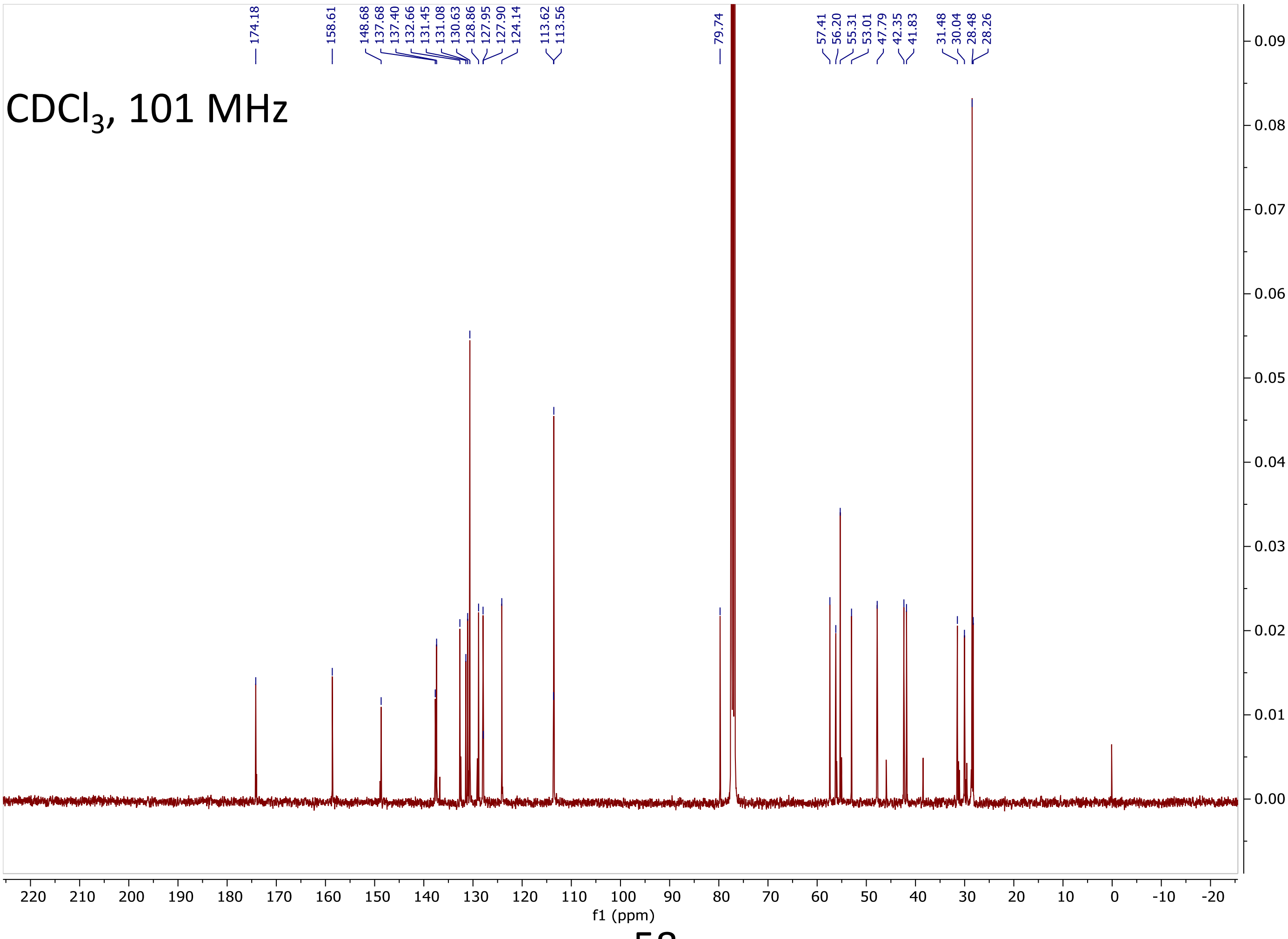
CDCl$_3$, 101 MHz
f1 (ppm)

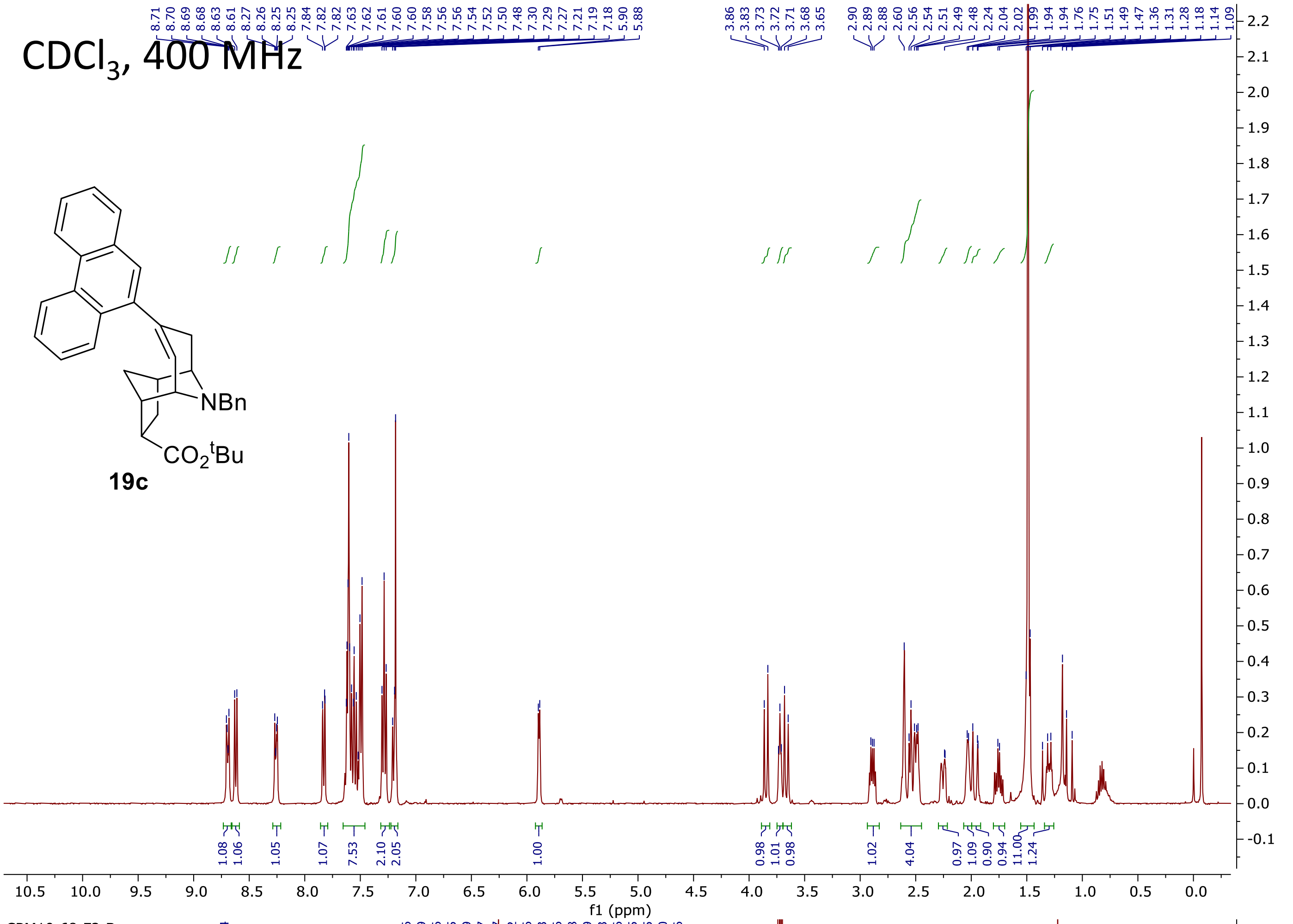
CDCl$_3$, 400 MHz
NBn
CO$_2$^{t}Bu
19c
8.71 8.70 8.69 8.68 8.63 8.61 8.27 8.26 8.25 8.25 7.84 7.82 7.82 7.63 7.62 7.61 7.60 7.58 7.56 7.56 7.54 7.52 7.50 7.48 7.30 7.29 7.27 7.21 7.19 7.18 5.90 5.88 3.86 3.83 3.73 3.72 3.71 3.68 3.65 2.90 2.89 2.88 2.60 2.56 2.54 2.51 2.49 2.48 2.24 2.04 2.02 1.99 1.94 1.94 1.76 1.75 1.51 1.49 1.47 1.36 1.31 1.28 1.18 1.14 1.09
1.08 1.06 1.05 1.07 7.53 2.10 2.05 1.00 0.98 1.01 0.98 1.02 4.04 0.97 1.09 0.90 0.94 11.00 1.24
f1 (ppm)

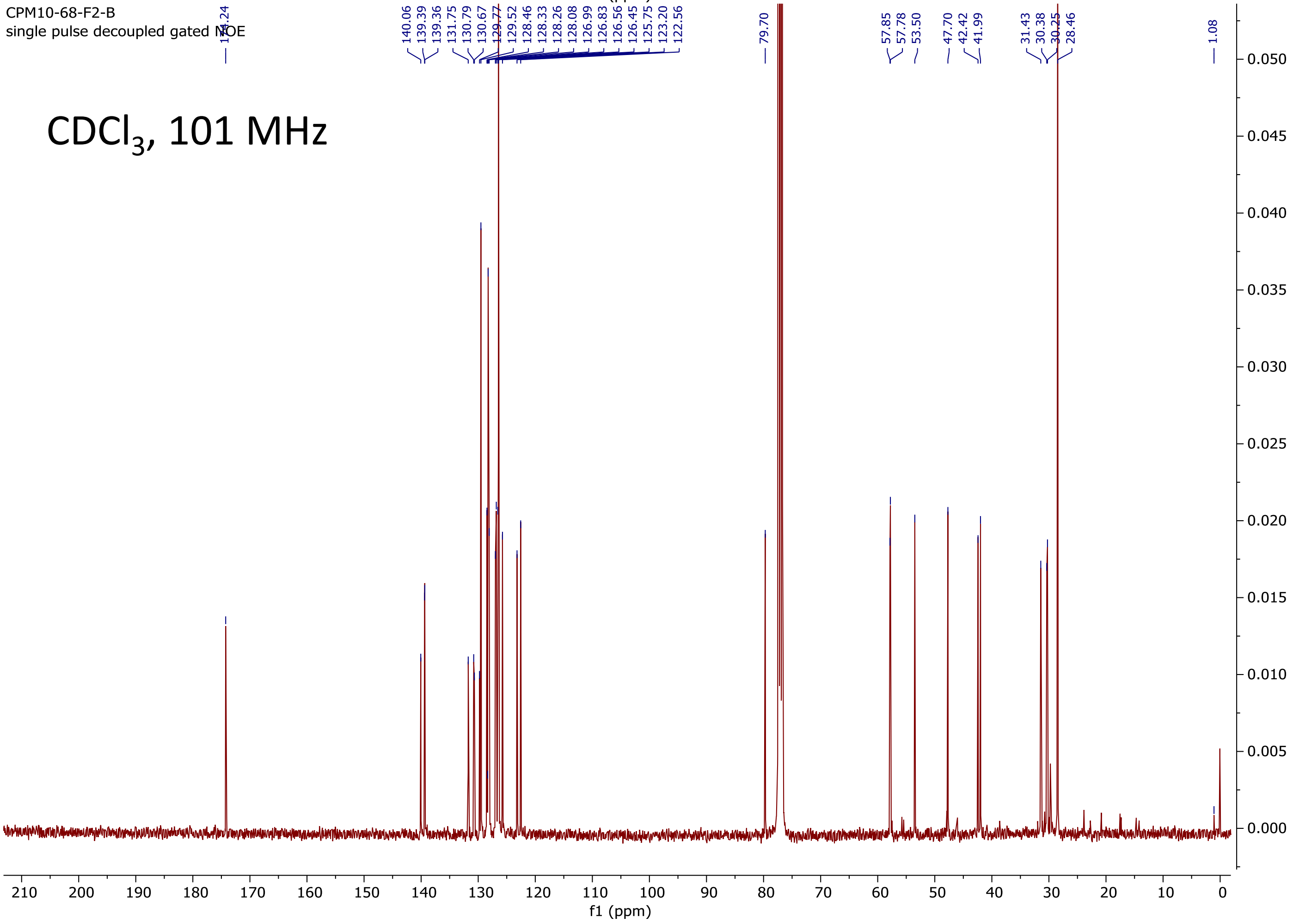
CPM10-68-F2-B
single pulse decoupled gated NOE
CDCl$_3$, 101 MHz
174.24 140.06 139.39 139.36 131.75 130.79 130.67 129.77 129.52 128.46 128.33 128.26 128.08 126.99 126.83 126.45 125.75 123.20 122.56 79.70 57.85 57.78 53.50 47.70 42.42 41.99 31.43 30.38 30.25 28.46 1.08
f1 (ppm)

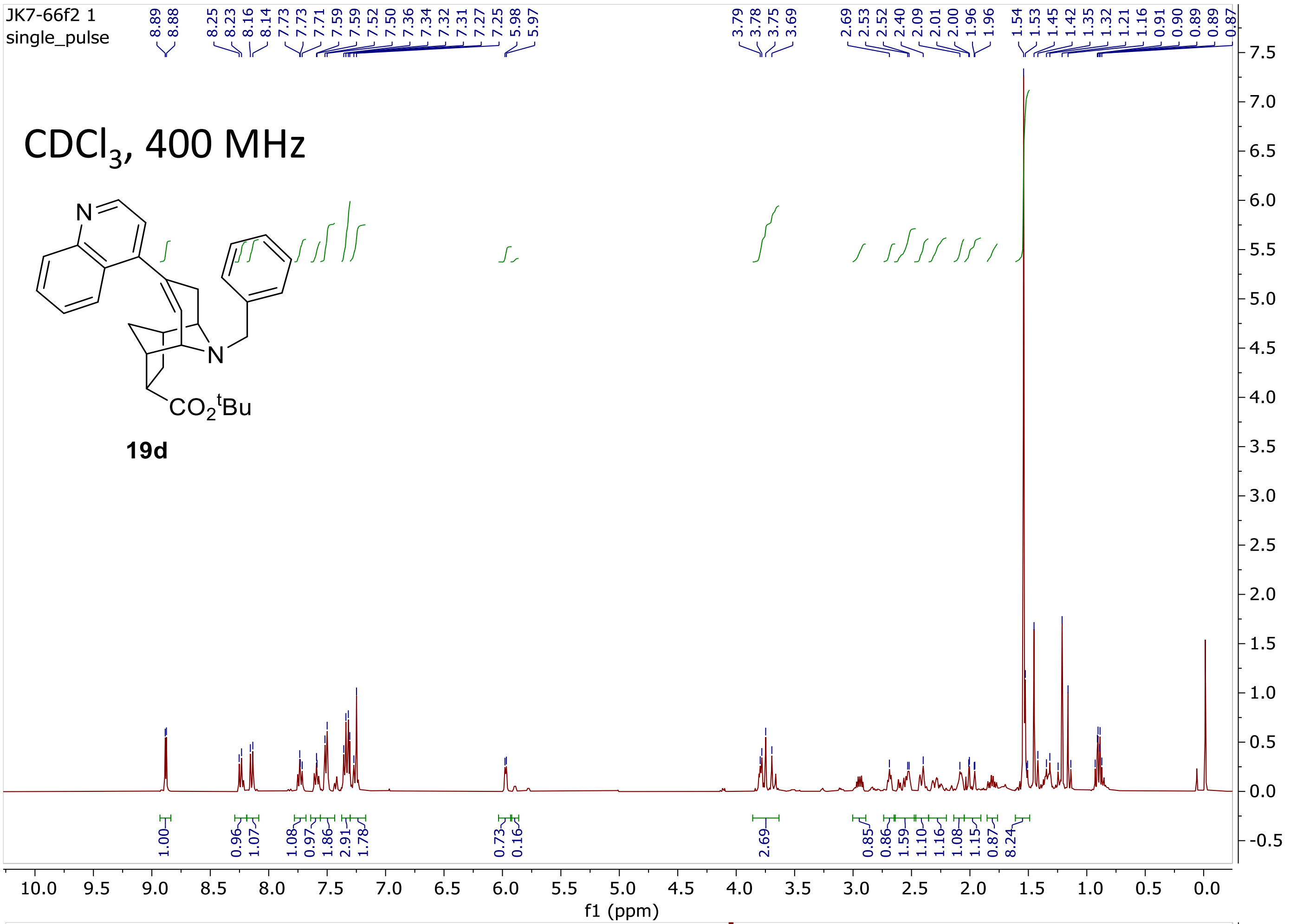
JK7-66f2 1
single_pulse
CDCl3, 400 MHz
N
N
CO2tBu
19d
8.89 8.88 8.25 8.23 8.16 8.14 7.73 7.73 7.71 7.59 7.59 7.52 7.50 7.36 7.34 7.32 7.31 7.27 7.25 5.98 5.97
3.79 3.78 3.75 3.69
2.69 2.53 2.52 2.40 2.09 2.01 2.00 1.96 1.96 1.54 1.53 1.45 1.42 1.35 1.32 1.21 1.16 0.91 0.90 0.89 0.89 0.87
1.00 0.96 1.07 1.08 0.97 1.86 2.91 1.78 0.73 0.16 2.69 0.85 0.86 1.59 1.10 1.16 1.08 1.15 0.87 8.24
f1 (ppm)

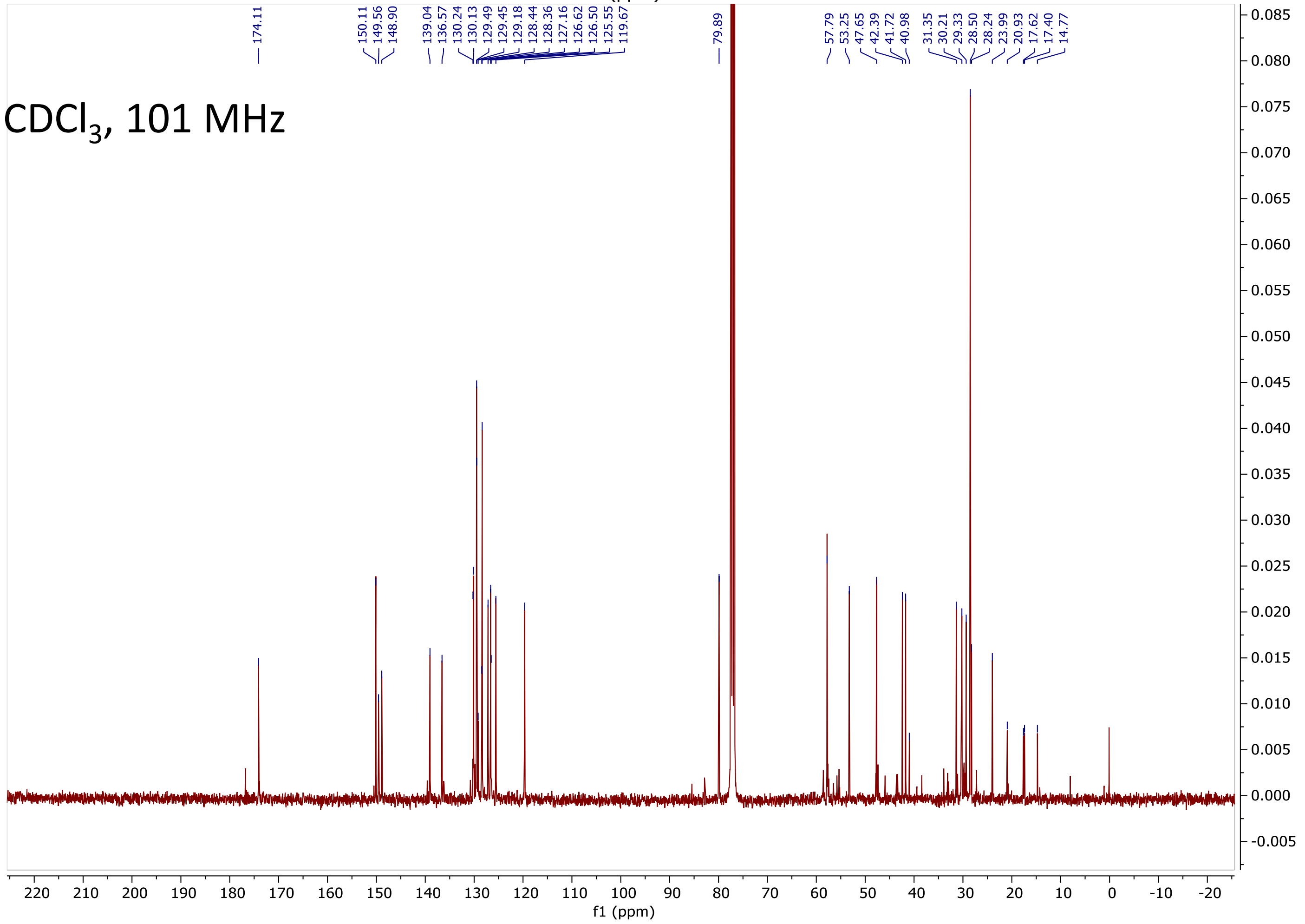
CDCl3, 101 MHz
174.11
150.11 149.56 148.90
139.04 136.57 130.24 130.13 129.49 129.45 129.18 128.44 128.36 127.16 126.62 126.50 125.55 119.67
79.89
57.79 53.25 47.65 42.39 41.72 40.98
31.35 30.21 29.33 28.50 28.24 23.99 20.93 17.62 17.40 14.77
f1 (ppm)

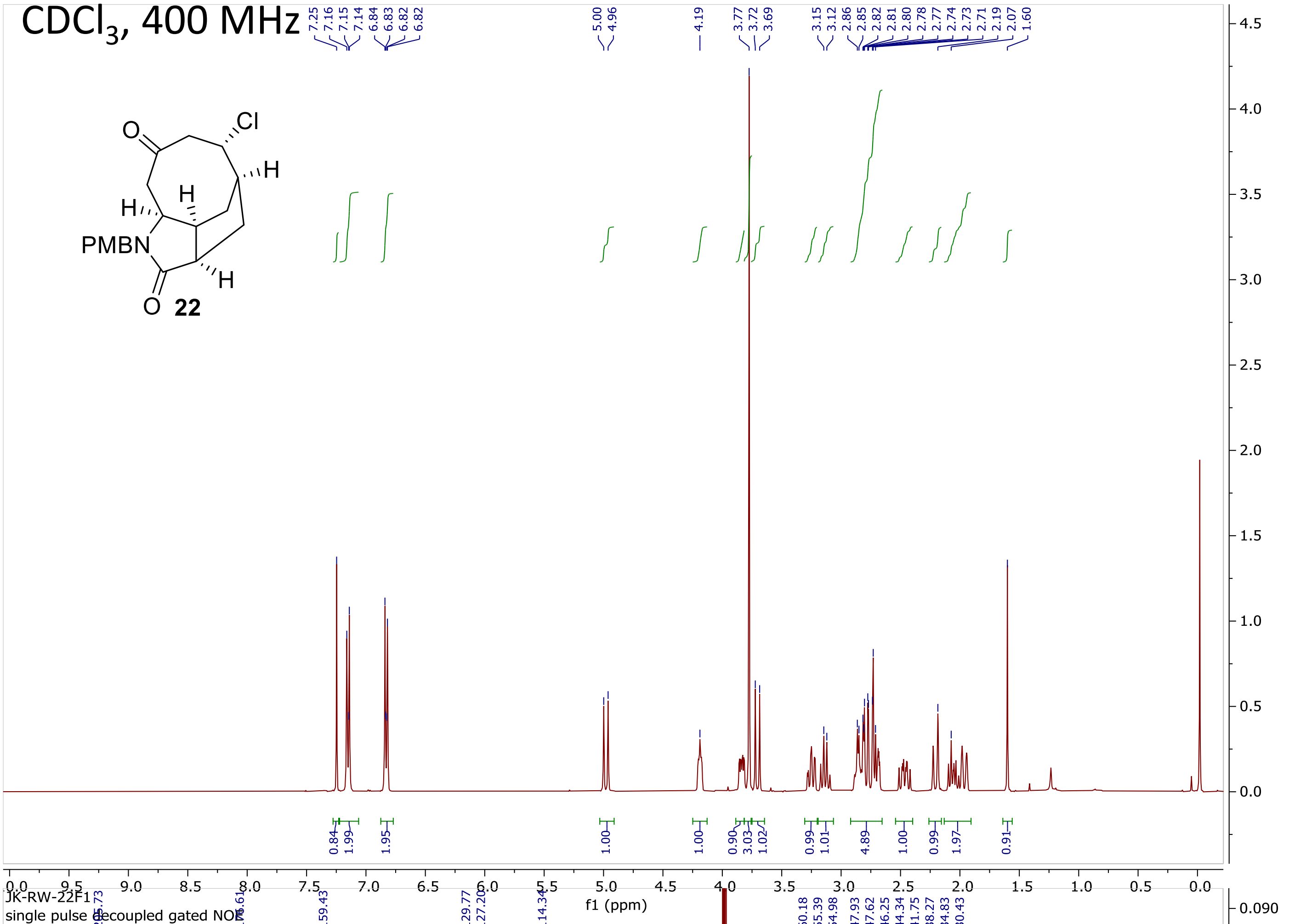

CDCl3, 400 MHz
7.25 7.16 7.15 7.14 6.84 6.83 6.82 6.82
5.00 4.96
4.19
3.77 3.72 3.69
3.15 3.12 2.86 2.85 2.82 2.81 2.80 2.78 2.77 2.74 2.73 2.71 2.19 2.07 1.60
O
Cl
H
H
H
PMBN
H
O
22
0.84 1.99 1.95 1.00 1.00 0.90 3.03 1.02 0.99 1.01 4.89 1.00 0.99 1.97 0.91
f1 (ppm)

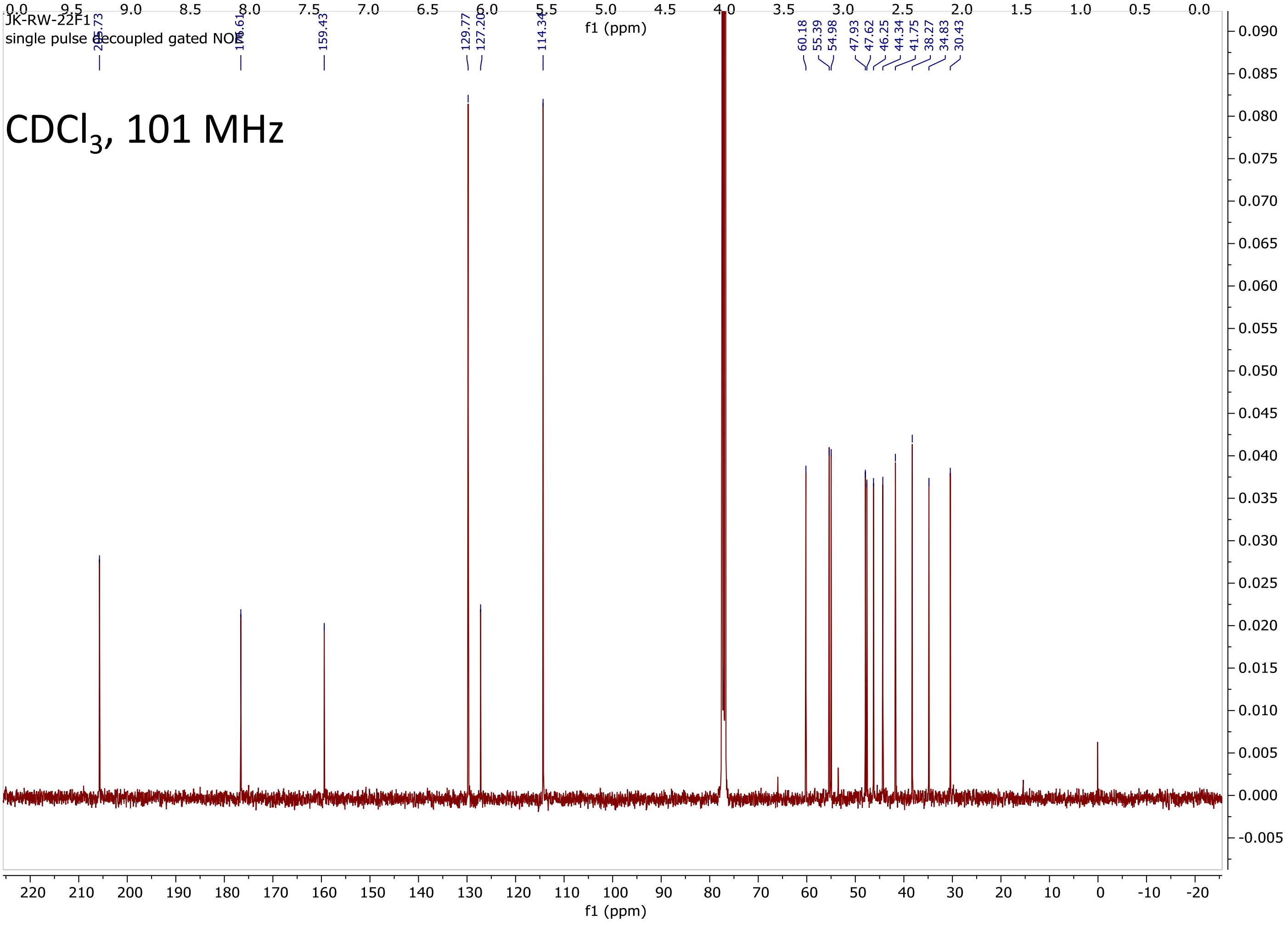

JK-RW-22F1
single pulse decoupled gated NOE
CDCl3, 101 MHz
205.73 176.61 159.43 129.77 127.20 114.34 60.18 55.39 54.98 47.93 47.62 46.25 44.34 41.75 38.27 34.83 30.43
f1 (ppm)

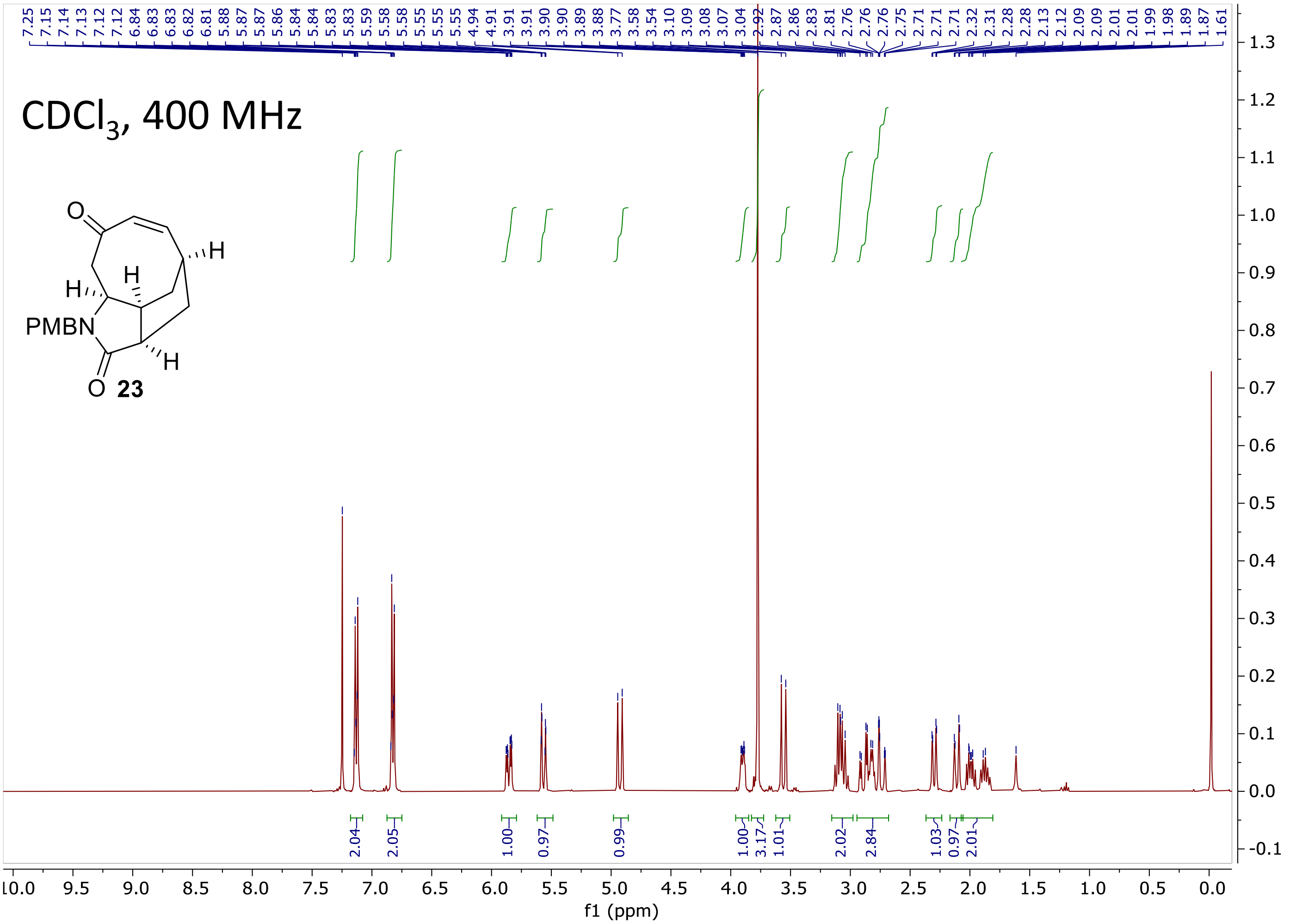
CDCl$_3$, 400 MHz
PMBN
23
f1 (ppm)

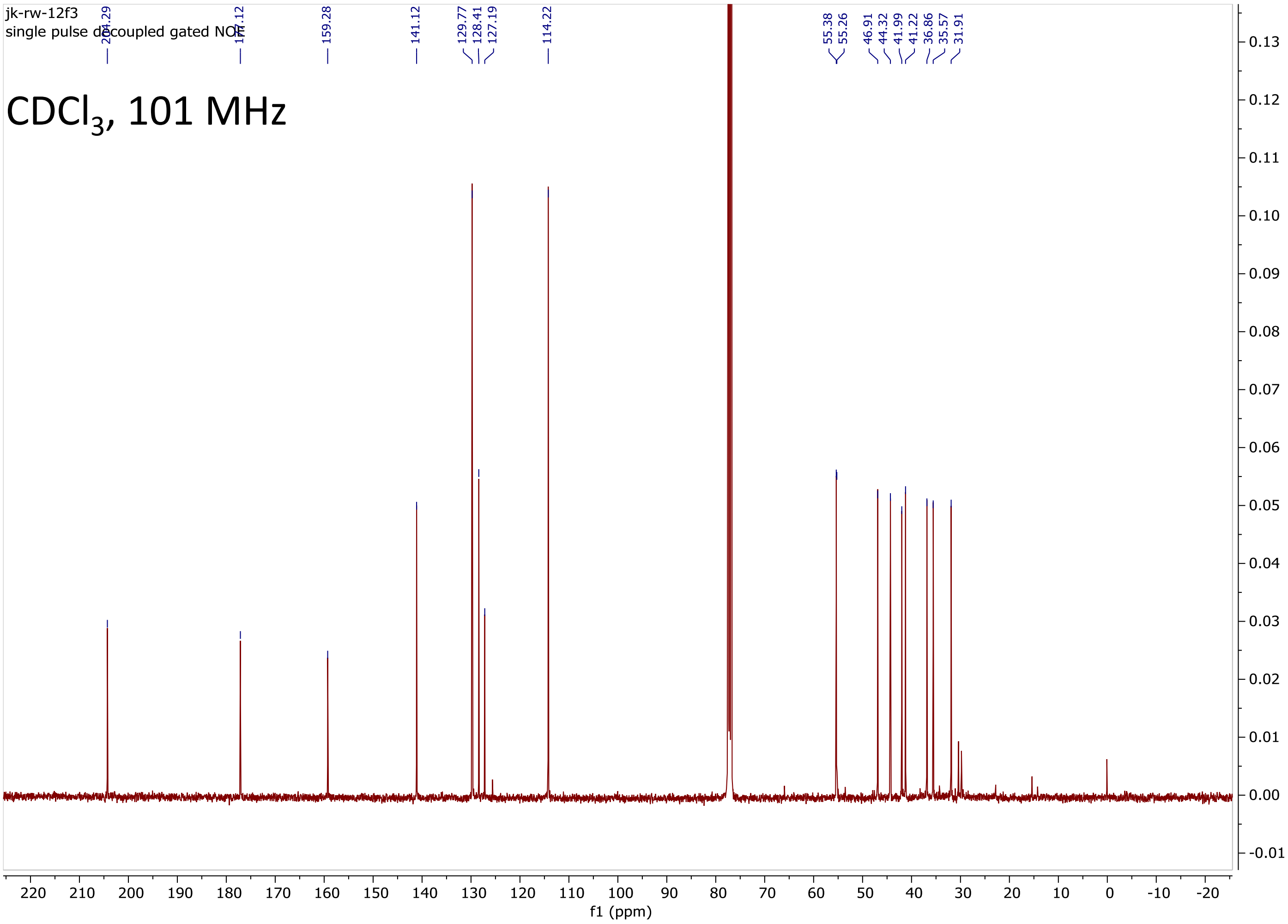
jk-rw-12f3
single pulse decoupled gated NOE
CDCl$_3$, 101 MHz
f1 (ppm)

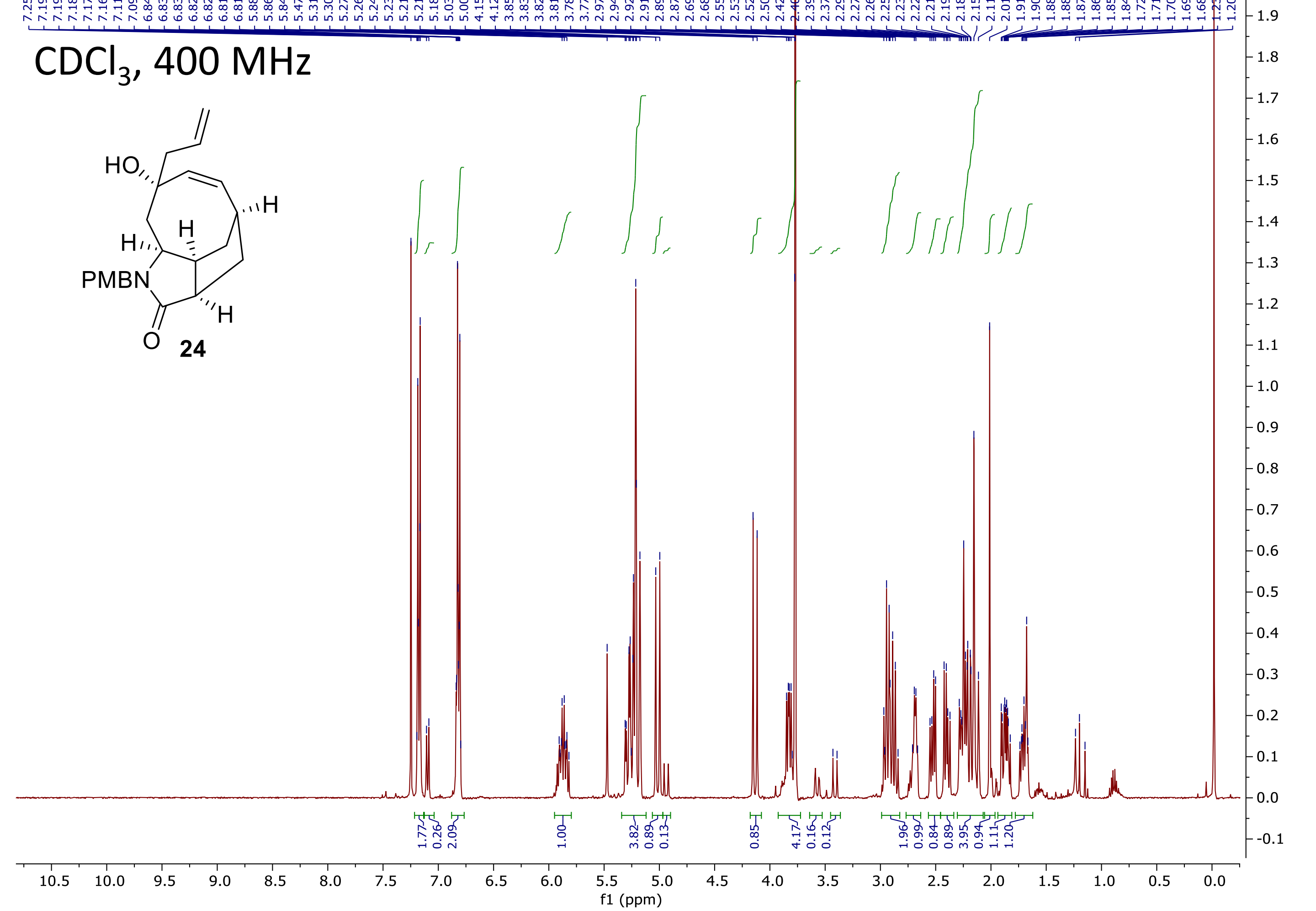
CDCl3, 400 MHz
HO
H
H
H
PMBN
O
H
24
f1 (ppm)

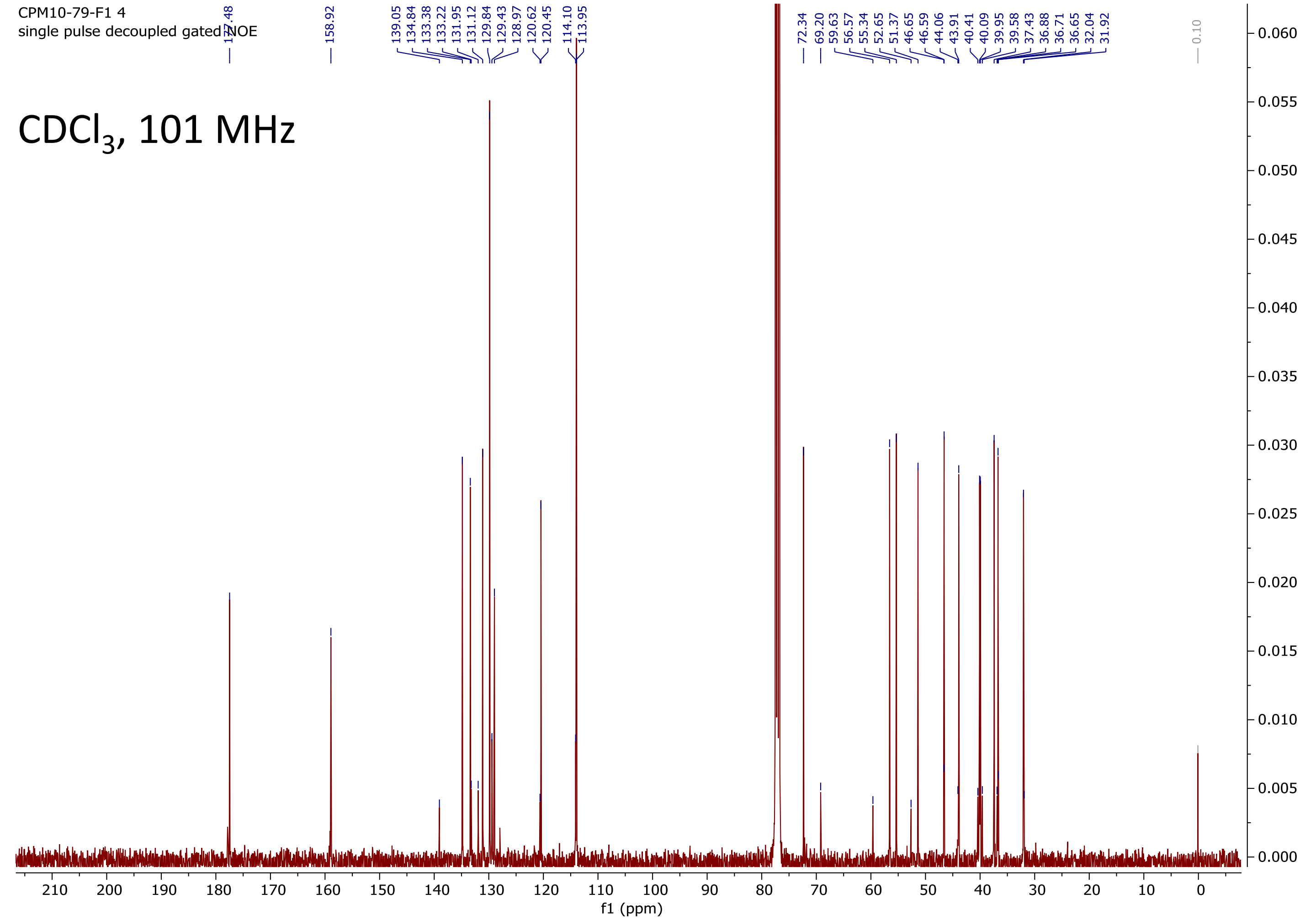
CPM10-79-F1 4
single pulse decoupled gated NOE
CDCl3, 101 MHz
177.48
158.92
139.05
134.84
133.38
133.22
131.95
131.12
129.84
129.43
128.97
120.62
120.45
114.10
113.95
72.34
69.20
59.63
56.57
55.34
52.65
51.37
46.65
46.59
44.06
43.91
40.41
40.09
39.95
39.58
37.43
36.88
36.71
36.65
32.04
31.92
0.10
f1 (ppm)

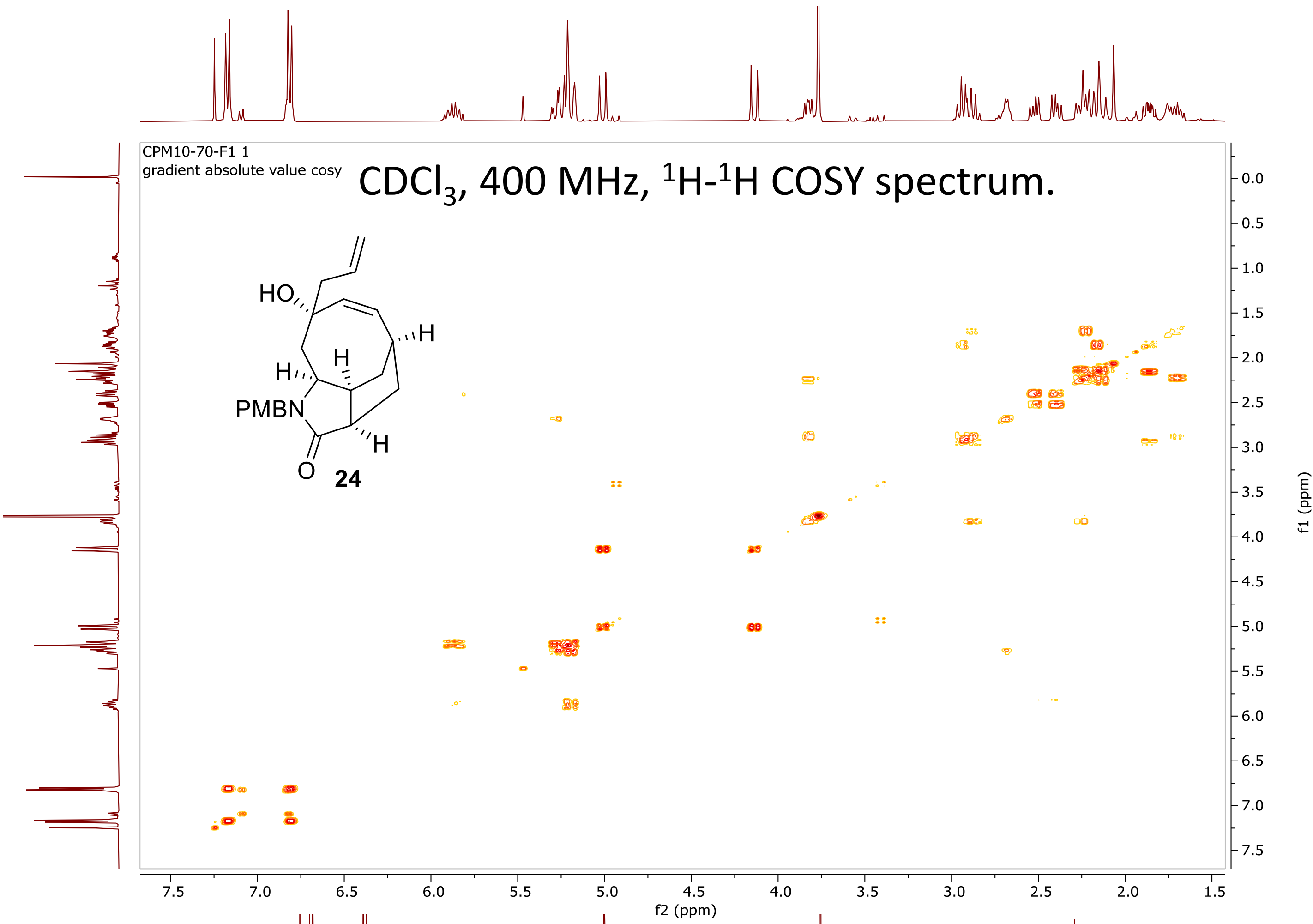
CPM10-70-F1 1
gradient absolute value cosy
CDCl3, 400 MHz, 1H-1H COSY spectrum.
HO
H
H
H
PMBN
H
O
24
f2 (ppm)
f1 (ppm)

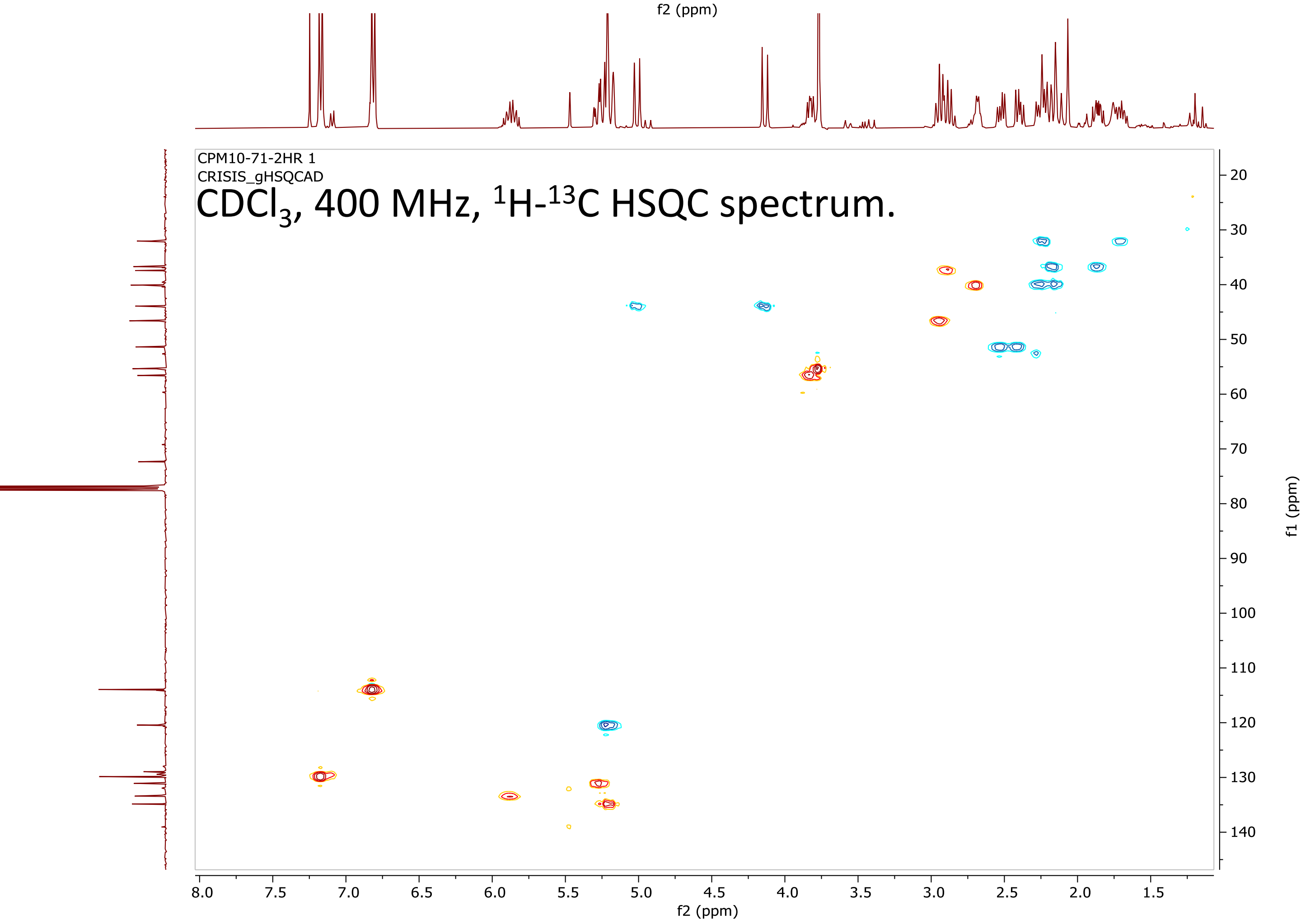
CPM10-71-2HR 1
CRISIS_gHSQCAD
CDCl3, 400 MHz, 1H-13C HSQC spectrum.
f2 (ppm)
f1 (ppm)

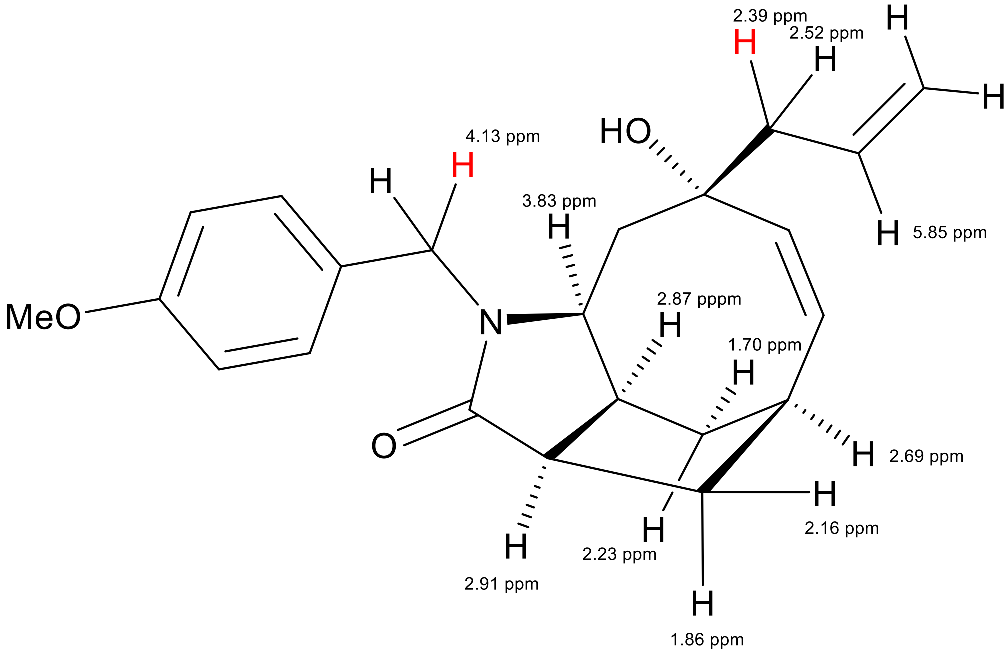
4.13 ppm
3.83 ppm
2.39 ppm
2.52 ppm
5.85 ppm
2.87 pppm
1.70 ppm
2.69 ppm
2.16 ppm
2.23 ppm
2.91 ppm
1.86 ppm
MeO
HO
N
O

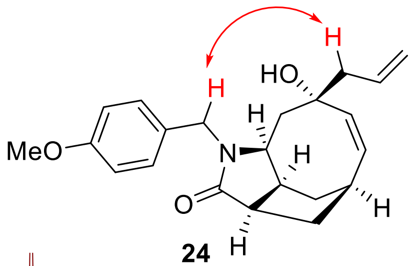
MeO
HO
N
O

24

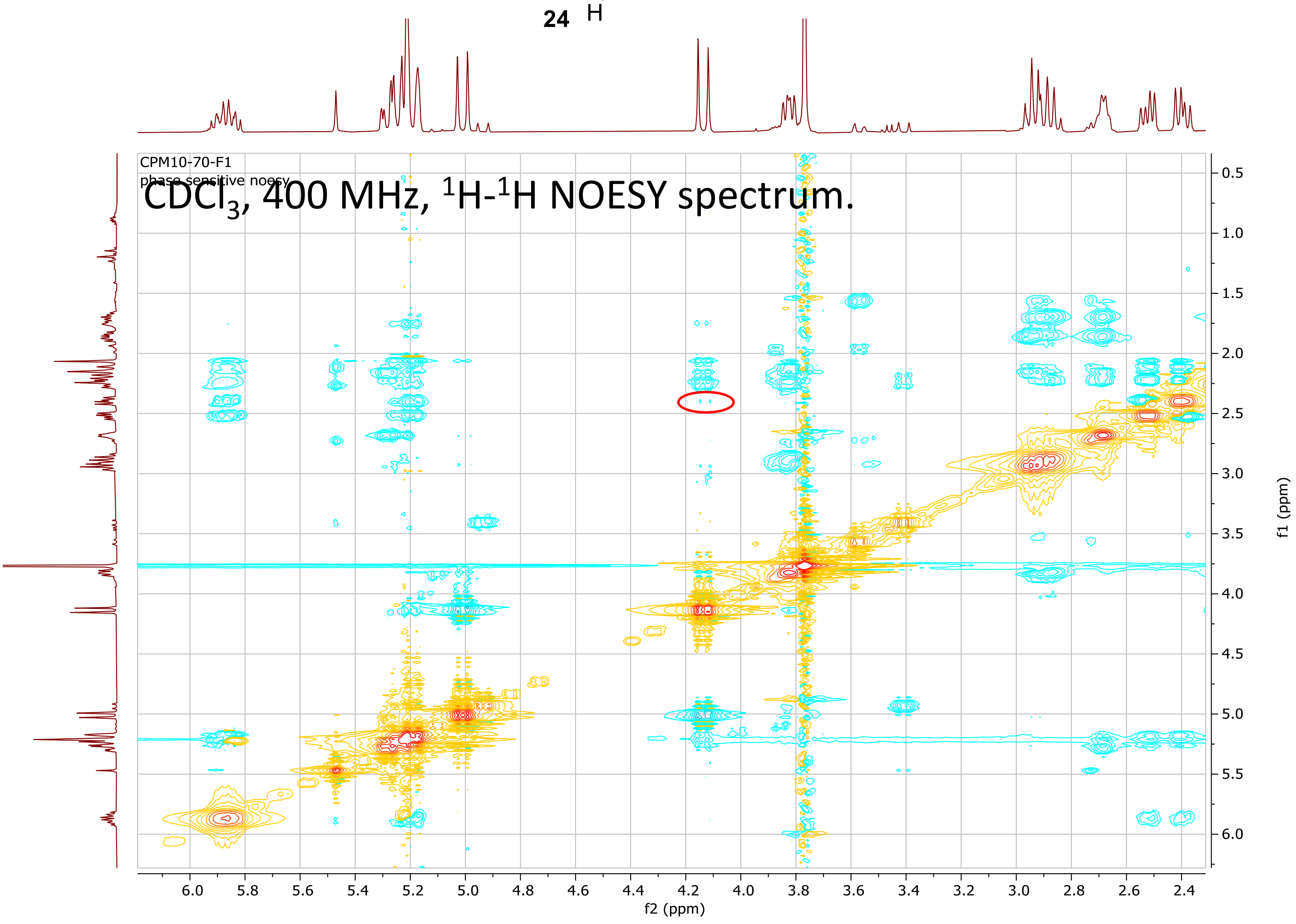
CPM10-70-F1
phase sensitive noesy
CDCl$_3$, 400 MHz, ^{1}H-^{1}H NOESY spectrum.
f2 (ppm)
f1 (ppm)

CDCl$_3$, 400 MHz

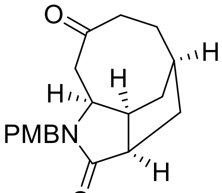

25

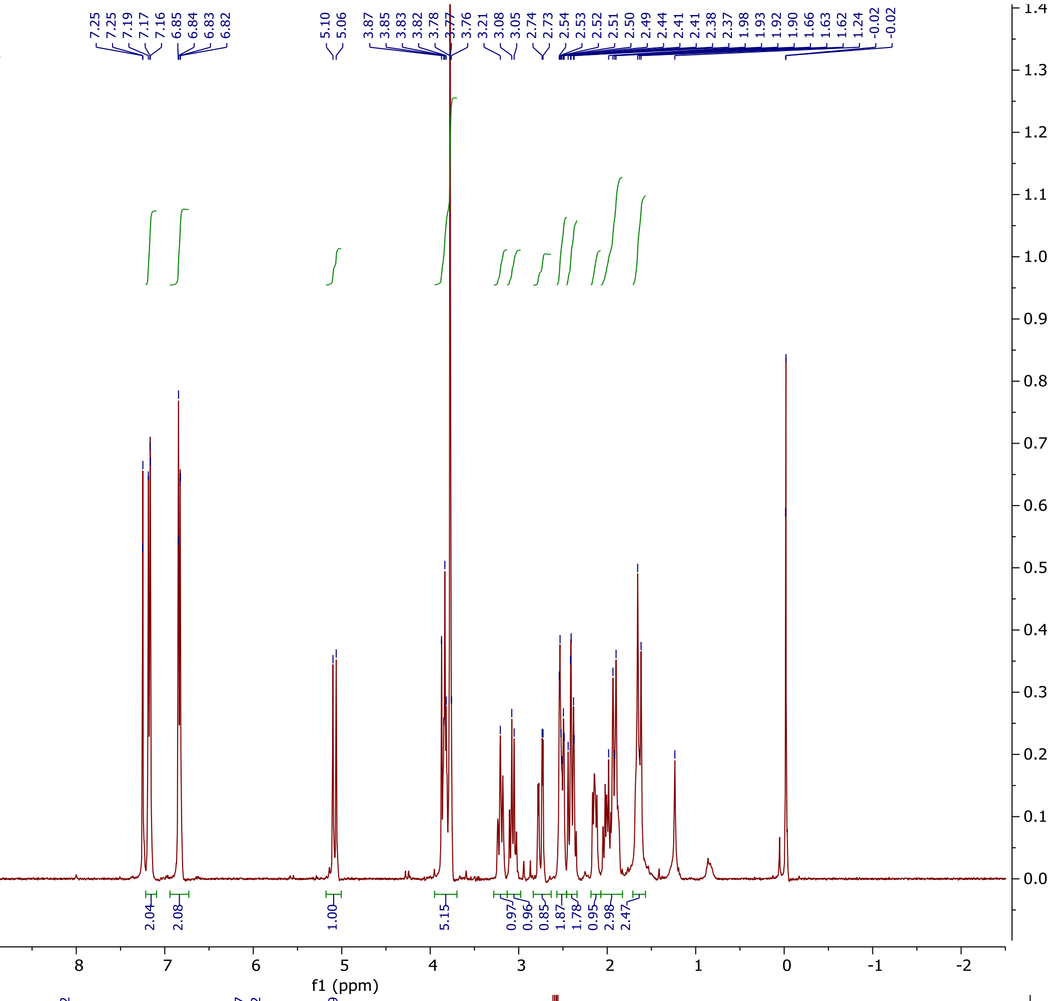

CDCl$_3$, 101 MHz

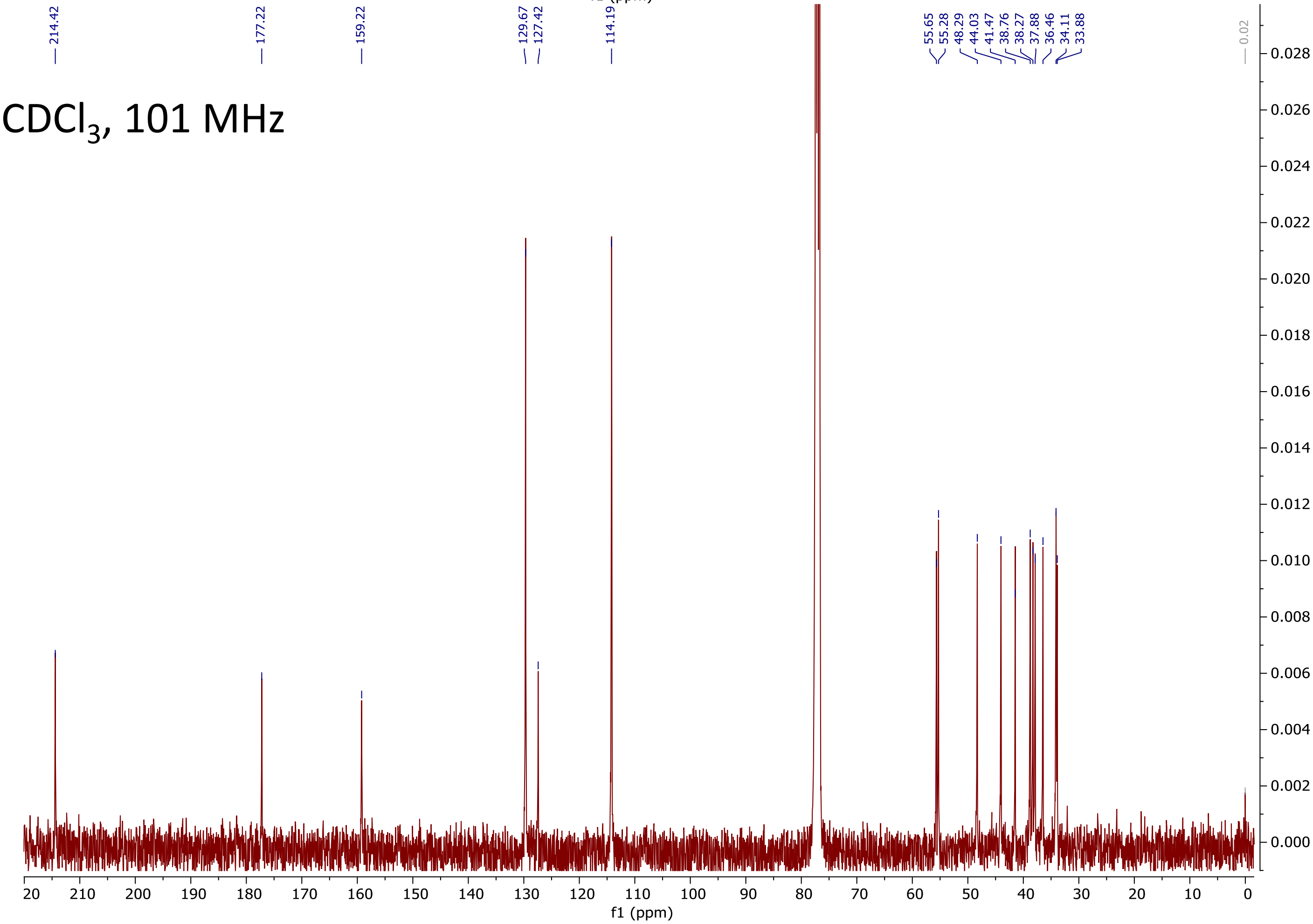

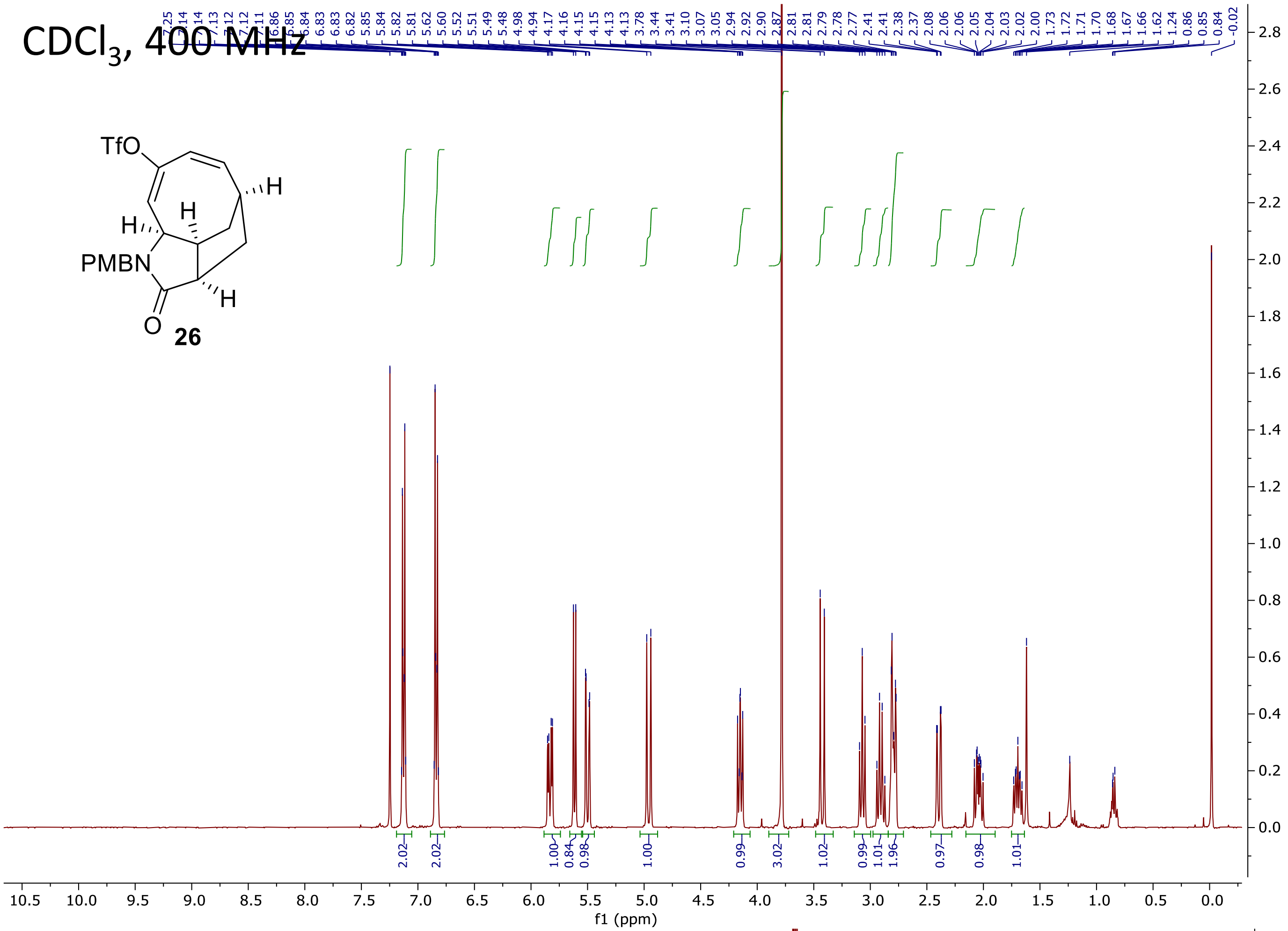
CDCl3, 400 MHz
TfO
H
H
PMBN
H
H
O
26
f1 (ppm)

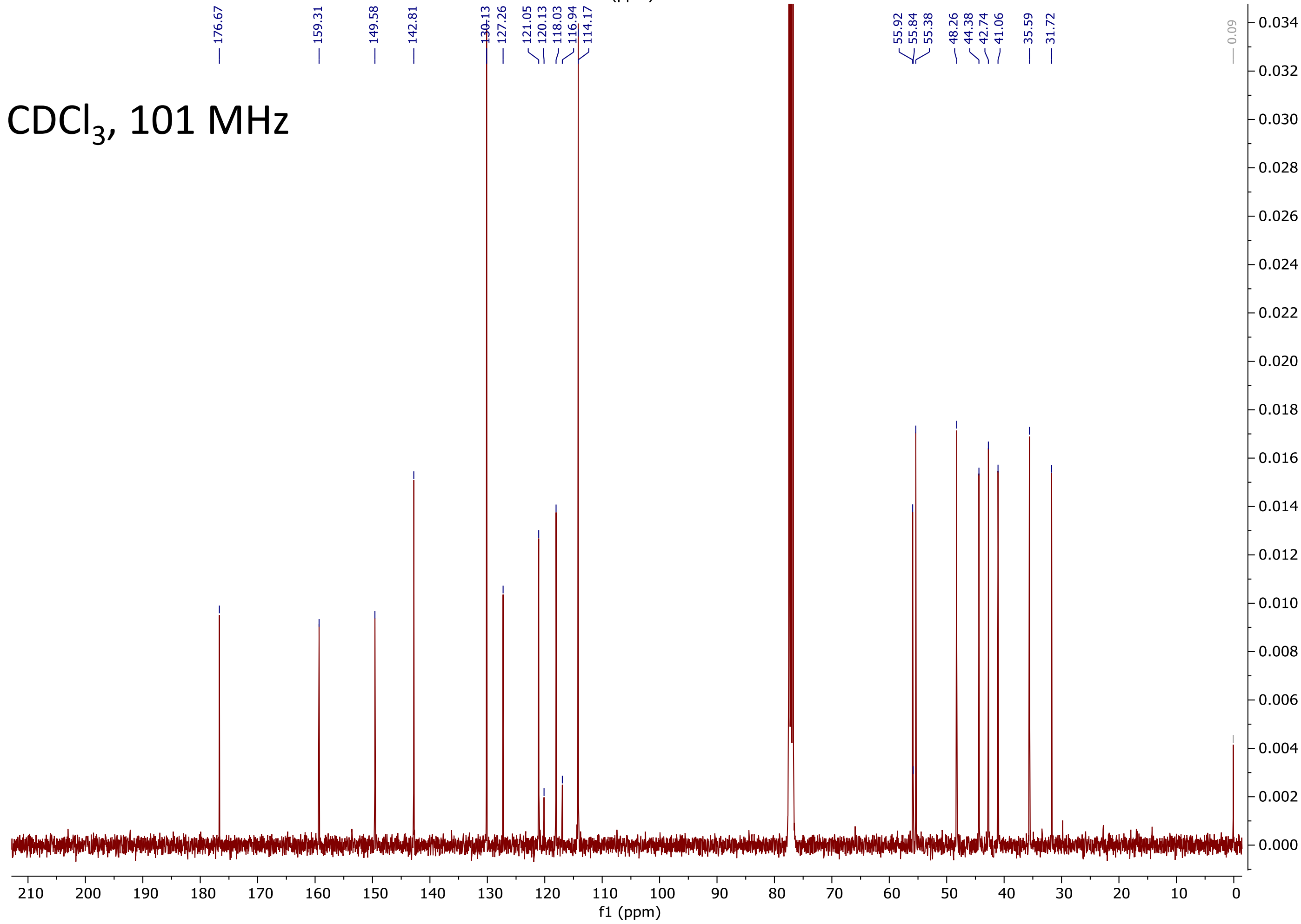
CDCl3, 101 MHz
176.67
159.31
149.58
142.81
130.13
127.26
121.05
120.13
118.03
116.94
114.17
55.92
55.84
55.38
48.26
44.38
42.74
41.06
35.59
31.72
f1 (ppm)

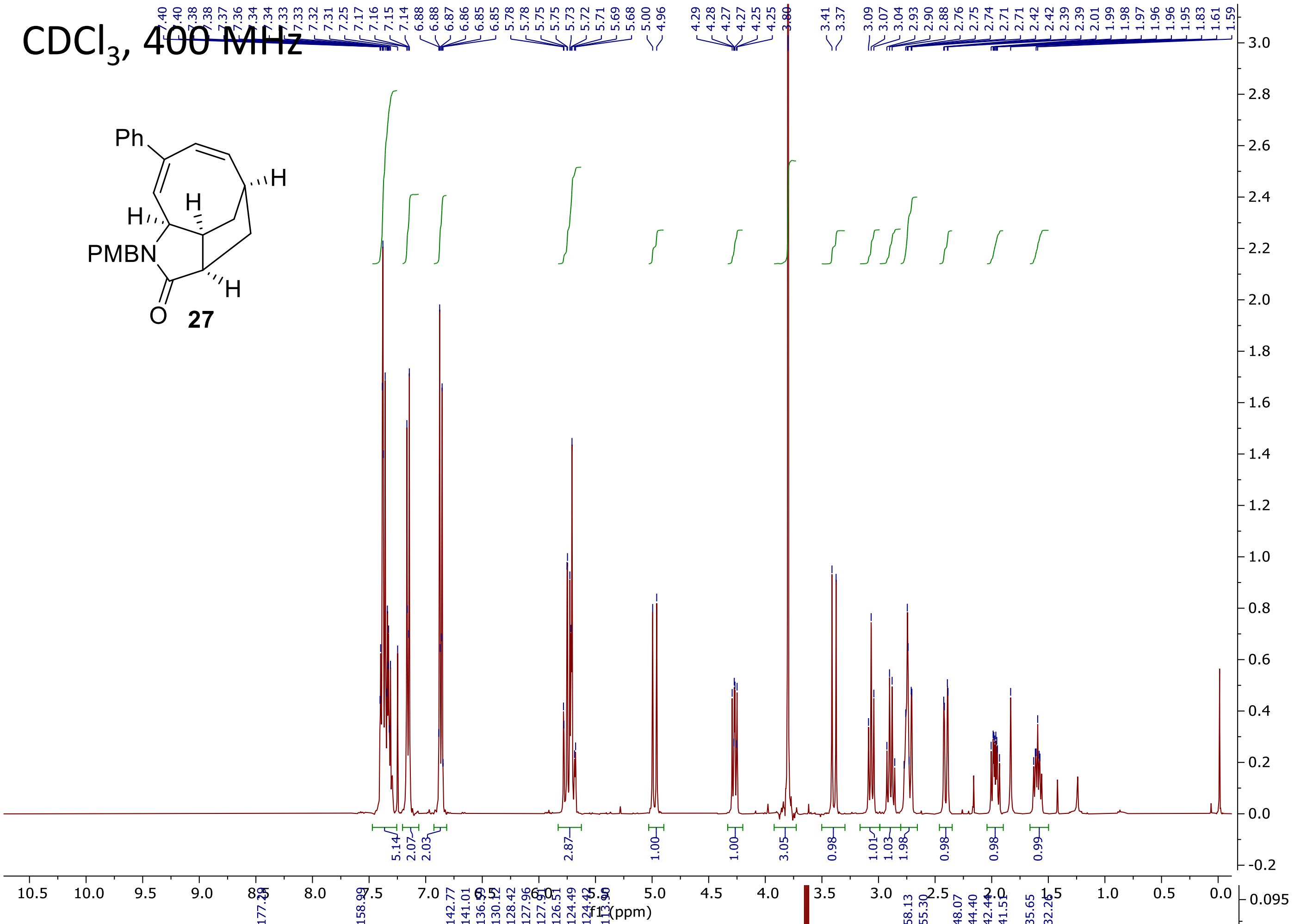
CDCl3, 400 MHz
Ph
H
H
PMBN
H
O
27
f1 (ppm)

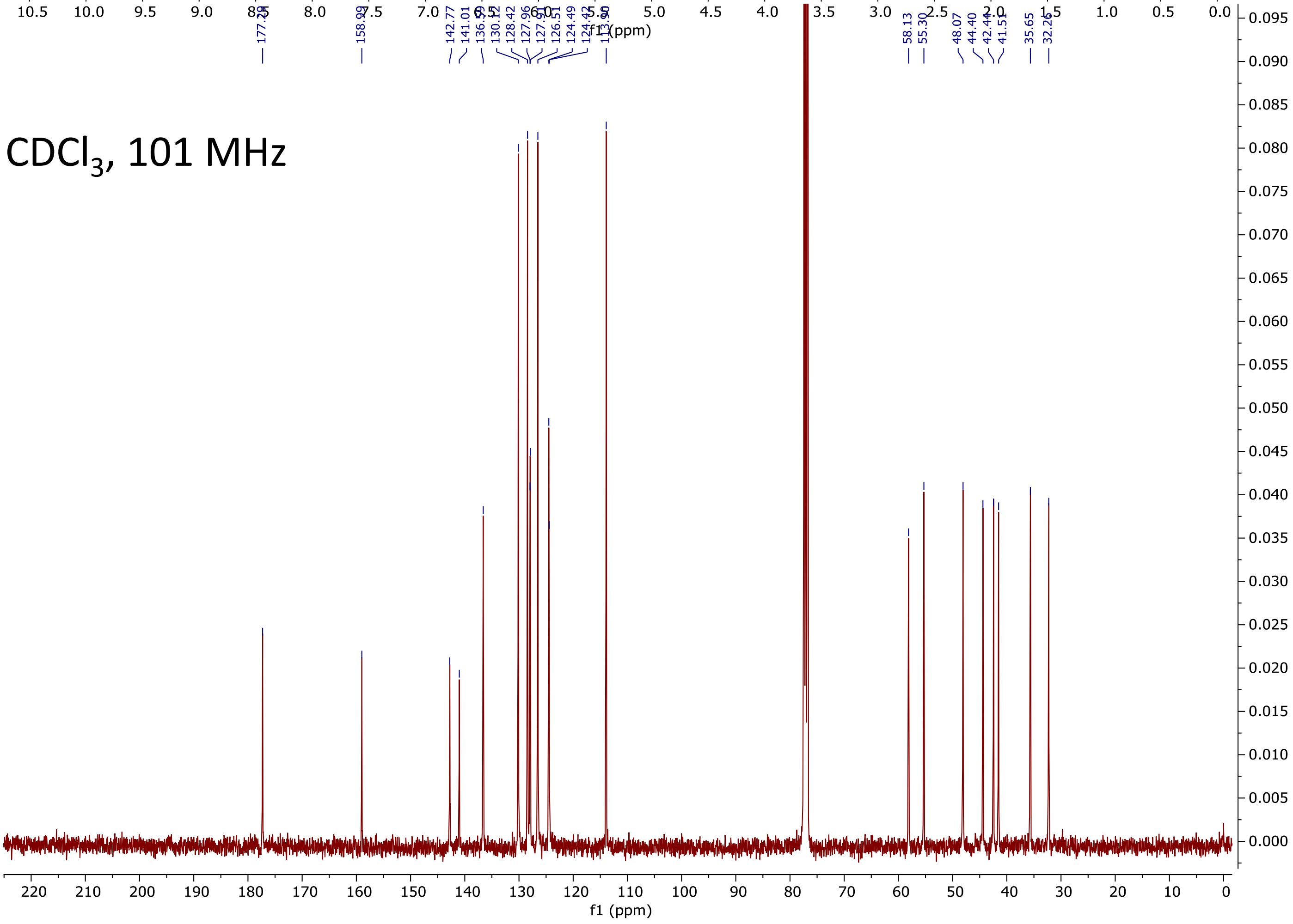
CDCl3, 101 MHz
f1 (ppm)

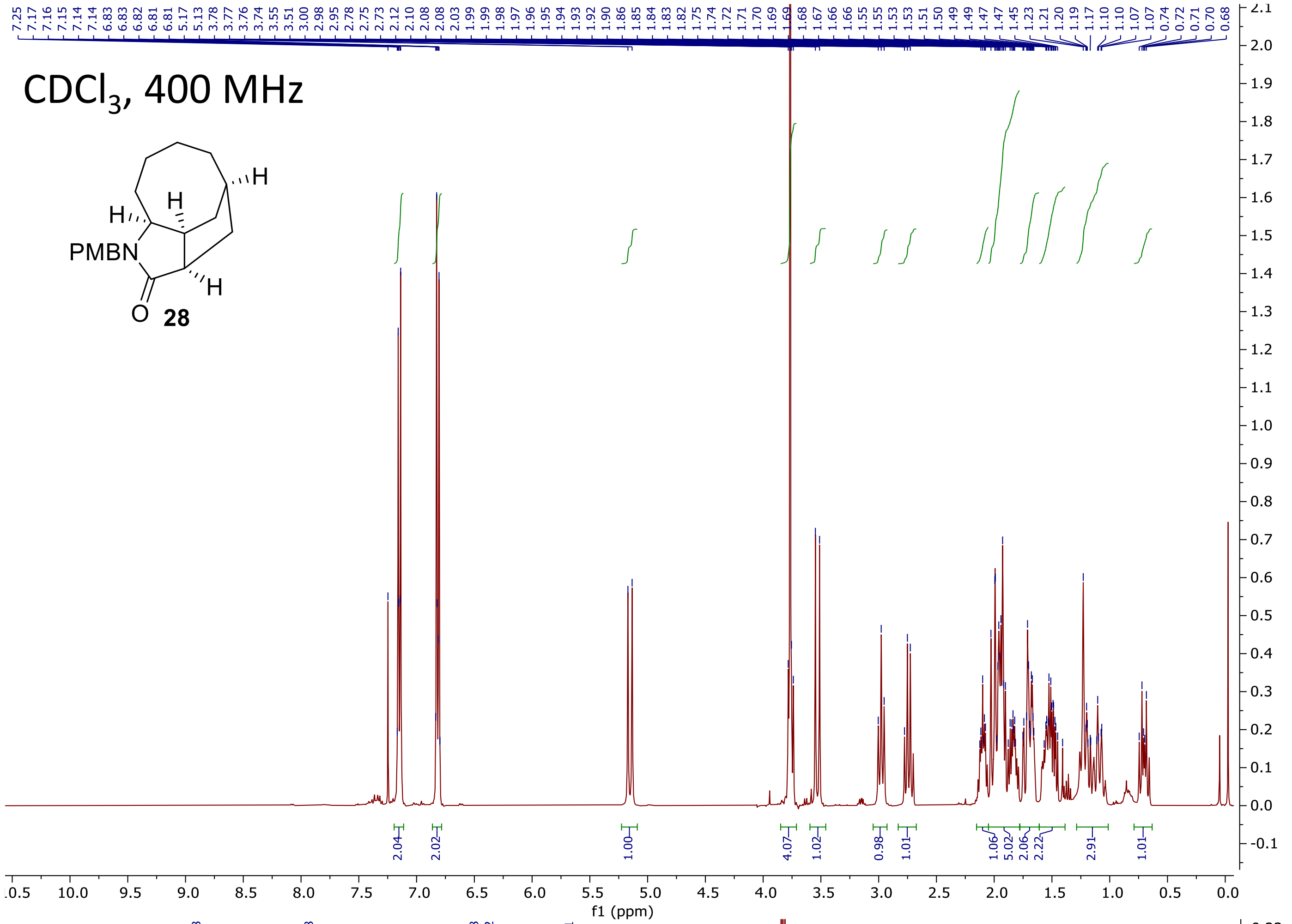
CDCl3, 400 MHz
PMBN
28
f1 (ppm)

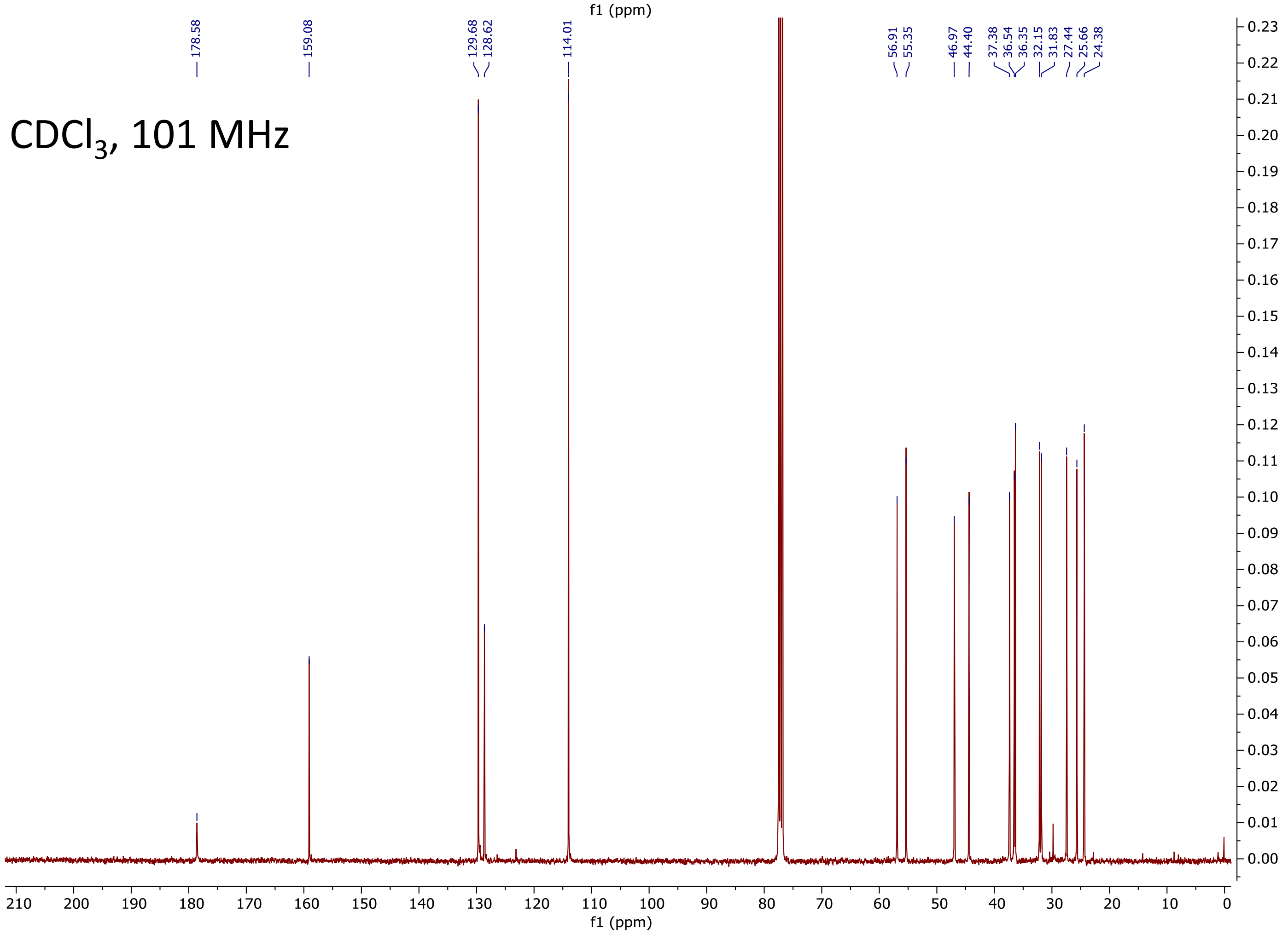
CDCl3, 101 MHz
178.58
159.08
129.68
128.62
114.01
56.91
55.35
46.97
44.40
37.38
36.54
36.35
32.15
31.83
27.44
25.66
24.38
f1 (ppm)
